# The Spalt Transcription Factors Generate the Transcriptional Landscape of the *Drosophila melanogaster* Wing Pouch Central Region

**DOI:** 10.1371/journal.pgen.1005370

**Published:** 2015-08-04

**Authors:** María F. Organista, Mercedes Martín, Jesus M. de Celis, Rosa Barrio, Ana López-Varea, Nuria Esteban, Mar Casado, Jose F. de Celis

**Affiliations:** Centro de Biología Molecular Severo Ochoa, CSIC and Universidad Autónoma de Madrid, C/Nicolás Cabrera, 1. Universidad Autónoma de Madrid, Madrid, Spain; Harvard Medical School, Howard Hughes Medical Institute, UNITED STATES

## Abstract

The Drosophila genes *spalt major (salm)* and *spalt-related (salr)* encode Zn-finger transcription factors regulated by the Decapentaplegic (Dpp) signalling pathway in the wing imaginal disc. The function of these genes is required for cell survival and proliferation in the central region of the wing disc, and also for vein patterning in the lateral regions. The identification of direct Salm and Salr target genes, and the analysis of their functions, are critical steps towards understanding the genetic control of growth and patterning of the Drosophila wing imaginal disc by the Dpp pathway. To identify candidate Salm/Salr target genes, we have compared the expression profile of *salm/salr* knockdown wing discs with control discs in microarray experiments. We studied by *in situ* hybridization the expression pattern of the genes whose mRNA levels varied significantly, and uncovered a complex transcription landscape regulated by the Spalt proteins in the wing disc. Interestingly, candidate Salm/Salr targets include genes which expression is turned off and genes which expression is positively regulated by Salm/Salr. Furthermore, loss-of-function phenotypic analysis of these genes indicates, for a fraction of them, a requirement for wing growth and patterning. The identification and analysis of candidate Salm/Salr target genes opens a new avenue to reconstruct the genetic structure of the wing, linking the activity of the Dpp pathway to the development of this epithelial tissue.

## Author Summary

How signalling pathways regulate the formation of organs with a precise size and pattern of differentiation is a fundamental question in developmental genetics. One classical example of the link between signalling and organ development is the regulation of wing disc development by the Decapentaplegic/BMP (Dpp) signalling pathway in Drosophila. A key outcome of this pathway is the transcriptional activation of the *spalt major* (*salm*) and *spalt related* (*salr*) genes, both encoding transcription factors. In this manner, the identification of Salm/Salr target genes is a critical step towards the understanding of the mode of action of these proteins and the genetic logic underlying the regulation of wing development by the Dpp signalling pathway. In order to identify these target genes, we used expression microarrays, *in situ* hybridization and phenotypic analysis. We identified an unexpected complexity in the transcriptional landscape of the wing disc that includes genes positively and negatively regulated by Salm/Salr. These findings have major implications for the reconstruction of the genetic hierarchy initiated by the Dpp pathway and leading to the formation of a wing with a correct size and pattern, because some of the genes we identified could explain particular aspects of the *sal* mutant phenotype.

## Introduction

The coordination of growth and patterning during the development of tissues and organs depends on the activity of signalling pathways acting in a context-dependent manner. For example the function of the Decapentaplegic (Dpp) signalling pathway is required to regulate cell viability and motility during dorsal closure [1], but the same pathway controls growth and patterning during imaginal disc development [2]. The developmental context is determined by the combinatory of transcription factors expressed in a given tissue, forming gene expression landscapes that influence cell behaviours and also control the response to universal signalling pathways.

The wing imaginal disc is an epithelial tissue that grows by cell proliferation during the larval development of the fly, and differentiates the wing and half of the thorax during pupal development [3]. The growth of the epithelium is accompanied by a progressive specification of spatial territories with different genetic identities. Several signalling pathways play a fundamental role during this process in part by regulating the expression of transcription factors. Among these pathways, the Dpp signalling pathway specifies the central region of the wing blade, its growth and patterning [2]. Several targets and additional components of the transcriptional regulation events triggered by Dpp signalling have being identified in Drosophila [2] including the T-box containing protein Bifid [4] and the Zn-fingers transcription factors Spalt major (Salm) and Spalt related (Salr) [5]. These proteins confer correct epithelial morphology and cell affinity to the central domain of the wing, and also regulate cell proliferation, viability and vein pattern formation [6–8].

Salm and Salr belong to a conserved family of transcriptional regulators that in vertebrates include four components (Spalt-like/Sall1-4) with important developmental roles during neural development and organogenesis [9]. In fact, two human *Sall* genes are related to the genetic diseases Townes Brocks Syndrome (SALL1) [10] and Okihiro Syndrome (SALL4) [11,12]. The Sal proteins can engage in a variety of interactions with other proteins and with DNA, and they can act as transcriptional repressors or activators [9,13–18]. Salm and Salr act as transcriptional repressors in Drosophila cultured cells, and the activity of at least Salr depends on the histone deacetylase complex NuRD [15]. However, the mechanisms by which Sal proteins regulate transcription are still not fully understood, although they include interaction with heterochromatic regions and recruitment of histone deacetylase complexes [13–15].

The *spalt* genes play a central role in mediating the effects of Dpp signalling during wing disc development [8], but the identity of Sal target genes is still unknown. Thus, only two gene complexes, the *knirps* and *Iroquois* gene complexes, have being identified as candidate downstream genes of Sal in the specification of vein territories [19,20]. However, Sal proteins are not only required for vein patterning, but also to promote cell division and survival in the central region of the wing [8], and they contribute to the maintenance of epithelial integrity in this territory [8,21]. The identity of the targets mediating these functional requirements is totally unknown. The identification of Salm/Salr target genes is of critical importance to understand the genetic hierarchy acting downstream of Dpp signalling in the wing disc.

Here we describe the global transcriptional changes that occur in *salm/salr* knockdown discs. These data, in combination with *in situ* hybridization assays and phenotypic analysis has allowed us to identify a collection of candidate genes that are regulated by the Sal proteins and might mediate their functions in the wing disc. Our work identifies an unsuspected transcriptional complexity occurring downstream of Spalt that involves repression as well as activation of gene expression.

## Results

### Genome-wide transcriptional changes observed in wing discs with reduced expression of *salm* and *salr*

**Fig 1:**
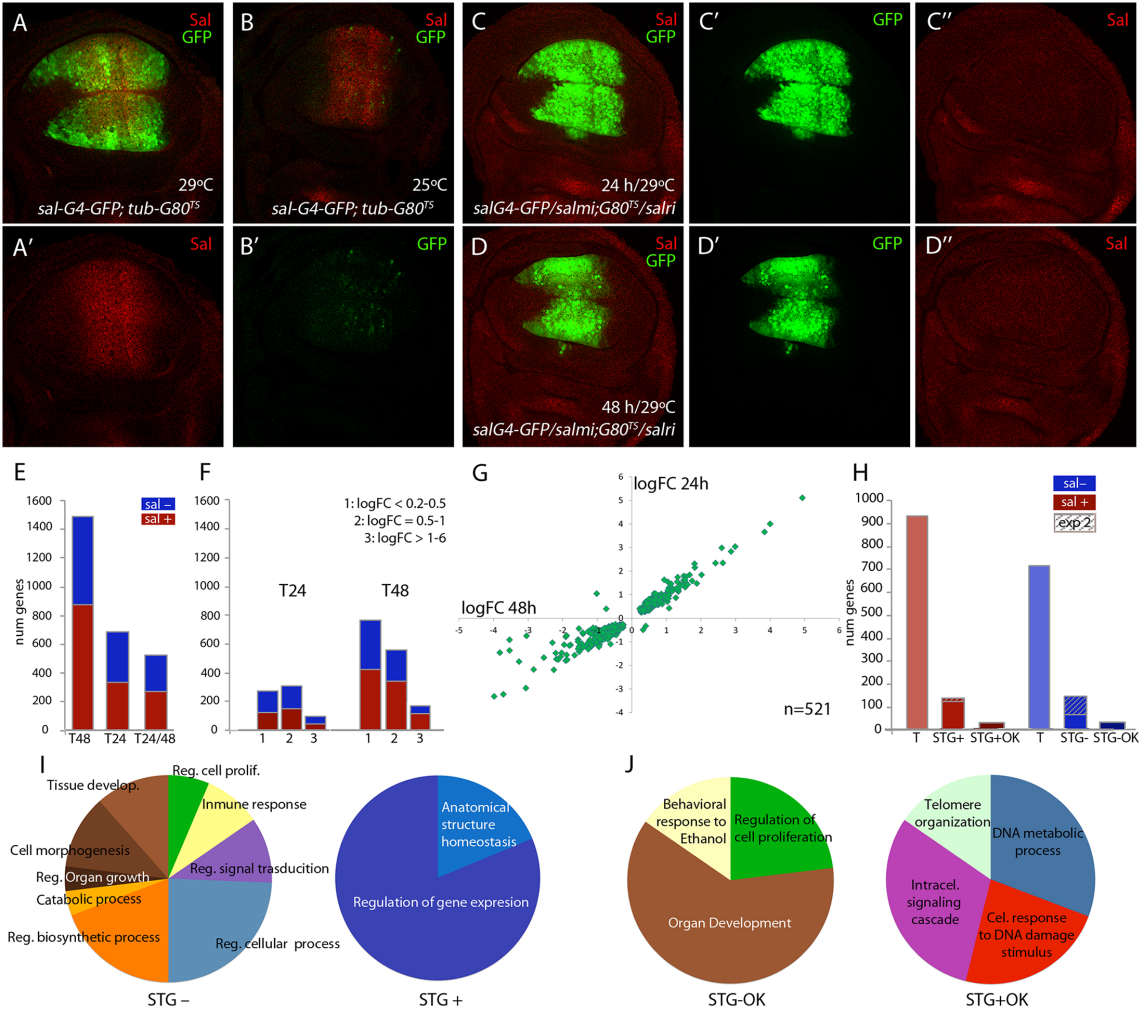
Global results of the genome-wide transcriptional changes observed in wing discs with reduced expression of *salm* and *salr*. (A-A’) Expression of Salm (red in A and A’) and *sal* ^*EPv*^ *-Gal4* (green in A) in *sal* ^*EPv*^ *-Gal4 UAS-GFP; tub-Gal80* ^*ts*^ */UAS-GFP* wing discs raised at 29°C. (B-B’) Expression of Salm (red in B) and *sal* ^*EPv*^ *-Gal4* (green in B’) in *sal* ^*EPv*^ *-Gal4 UAS-GFP; tub-Gal80* ^*ts*^ */UAS-GFP* wing discs raised at 25°C. (C-C’’) Imaginal disc of *sal* ^*EPv*^ *-Gal4 UAS-GFP /UAS-salm-i; tub-Gal80* ^*ts*^ */UAS-salr-i* genotype (*salm-i/salr-i* 24h) raised at 29°C 24–28 hours before dissection, showing the expression of GFP (green in C and C’) and Salm (red in C and C’’). (D-D’’) Imaginal disc of *sal* ^*EPv*^ *-Gal4 UAS-GFP /UAS-salm-i; tub-Gal80* ^*ts*^ */UAS-salr-i* genotype (*salm-i/salr-i* 48h) raised at 29°C 44–48 hours before dissection showing the expression of GFP (green in D and D’) and Salm (red in D and D’’). (E) Number of genes which expression level changes with an adjusted p-value lower than 0.05 that were identified in the comparisons with controls of *salm-i/salr-i* at 48h (T48); *salm-i/salr-i* at 24h (T24) and at both time intervals (T24/T48). The blue and red sections of each column correspond to genes showing reduced (sal-; blue) and increassed (sal+; red) expression in *salmi/salri* compared to their corresponding controls. (F) Number of genes identified at T24 and T48 grouped by logFoldChange values. Blue represents genes which expression is reduced and red those genes which expression is increased. 1: logFC lower than 0.5; 2: logFC between 0.5 and 1 and 3: logFC major than 1. (G) Graphical representation of the logFoldChange values for those genes that were identified both in *salm-i/salr-i* 48h and *salm-i/salr-i* 24h. (H) Total number of identified genes from experiment 1 (T) with increased (sal+; red) and reduced (sal-; blue) expression in *salm-i/salr-i* discs compared to controls. Number of selected genes coming from experiments 1 and 2 (STG) with increased (STG+) and reduced (STG-) expression. Genes selected from experiment 2 are indicated by stripped columns. Number of best candidate sal repressed (STG+OK) and activated (STG-OK) genes. (I-J) Number of genes grouped in GO categories enriched for STG- and STG+ genes (I) and for STG-OK and STG+OK genes (J).

In third instar wing discs *salm* and *salr* are expressed in a broad stripe of cells in the wing pouch (Fig 1A-1A’) that corresponds in the adult wing to a region including the vein L2 and extending to the L4/L5 intervein. This expression is directly regulated by Dpp signalling [22]. It is likely that Salm/Salr regulate the expression of a collection of target genes that in turn mediate the variety of functions assigned to them. To identify “Salm/Salr Target Genes” (STG), we compared the expression profile of wing discs in which the expression of both *salm* and *salr* genes was reduced by RNA interference with control wing discs. For these experiments, we used the *sal* ^*EPv*^ *-Gal4* driver, which reproduces the domain of *salm/salr* expression in the wing blade region of the imaginal disc [23] (Fig 1A). In addition, we took advantage of the temperature-sensitive Gal80 protein, which blocks the activity of Gal4 at 25°C but is inactive at 29°C [24]. Thus, in *sal* ^*EPv*^ *-Gal4 UAS-GFP; tub-Gal80* ^*ts*^ */UAS-GFP* discs grown at 25°C the expression of GFP was undetected in the wing blade (Fig 1B’), and the expression of Salm was unaffected (Fig 1B). This allowed us to precisely determine the time since when we started expressing the *salm* and *salr* RNA interferences. We chose to compare two time points, one 24 hours and other 48 hours after a 25°C to 29°C temperature shift, as this might allow to distinguish direct and indirect Salm/Salr transcriptional effects. We found that after 24 hours at 29°C (T24) the expression of Salm was already undetected in the wing blade (Fig 1C-1C’’ *; sal* ^*EPv*^ *-Gal4 UAS-GFP /UAS-salm-i; tub-Gal80* ^*ts*^ */ UAS-salr-i*), whereas the expression of GFP was strong in the central domain of the wing (Fig 1C-1C’). As expected, we also found that GFP expression was robust and Salm expression lost after 48 hours at 29°C (T48) (Fig 1D-1D’’). Wing discs observed at T48 differ from those analysed at T24 in that they displayed a severer phenotype of reduction in the size of the *sal* ^*EPv*^ *-Gal4* expression domain (compare Fig 1D and 1C). Older discs also showed a loss in FasIII expression (S1 Fig), indicating that the developmental defects characteristics of *salm/salr* loss develop progressively over time.

In the case of control discs (*sal* ^*EPv*^ *-Gal4 UAS-GFP; tub-Gal80* ^*ts*^ */UAS-GFP*) we could not detect any difference between the expression profiles of discs grown 24 or 48 hours at 29°C. In contrast, in *sal* ^*EPv*^ *-Gal4 UAS-GFP/UAS-salm-i; tub-Gal80* ^*ts*^ */UAS-salr-i* (*salm-i/salr-i*) discs we detected 71 genes whose expression changes comparing discs at T48 and T24 (S1 Table). Most of these cases (69) correspond to genes whose expression was increased at T48 compared to T24. We also compared *salm-i/salr-i* and control discs at T24 and T48. In the first case (T24) we detected significant changes (adjusted p-value lower than 0.05) in the expression levels of 686 genes, of which 98 displayed a fold-change higher than 2 (Fig 1E and 1F and S2 Table). The number of changes was much higher at T48, where we found changes in the expression levels of 1488 genes, including 168 with a fold-change higher than 2 (Fig 1E and 1F; S2 Table). Most of the genes whose expression level changed at T24 were also detected at T48 hours (n = 521 out of 686). In these cases, there was a good correlation between the extent of change observed in the T24 and T48 classes (Fig 1G). Genes that were only detected at T24 (165) in general had low fold-change values, and only 10 had a fold-change higher than 2 (S2 Table). These lists of genes show a low overlap with other transcriptomic and genomic analyses carried out in the wing disc. For example, we only could find 13 coincidences with the list of 146 genes enriched in the notum or wing regions of the wing disc [25], 40 coincidences with the 1649 genes associated to Brinker binding peaks [26] and 10 coincidences with the 4954 genes identified as modified comparing *sal* mutant embryos with control ones [27]. These results indicate that the collection of genes identified in our microarrays corresponds to a novel set of genes that were not selected before in other genomic studies in the wing disc.

From the total of genes that showed a significant change comparing *salm-i/salr-i* and control discs at T24 and T48, we decided to select those ones that changed with a logFC of at least 1. We distinguished between those that are present at both time points (n = 124) and those that changed only at T24 or T48 (n = 67). A total of 51 genes from these 191 selected ones (30%) were also identified in the microarrays described in experiment 2, in which we compared expression profiles from discs over-expressing *sal* (UAS-sal) and disc carrying *salm/salr* mutant clones with their respective controls (see material and methods; experiment 2). To generate a collection of candidates STG for subsequent analysis, we added a total of 95 genes that were only selected in experiment 2 with the best scores (Fig 1H). The joint list of STG (n = 286) includes 139 genes whose expression increases in *salm/salr* loss of function (STG+; Fig 1H and S3 Table) and 147 whose expression levels decreases in *salm/salr* loss of function conditions (STG-; Fig 1H and S3 Table). Based on subsequent in situ hybridization data and phenotypic analysis (see below), we further selected from the STG+ and STG- groups a collection of 32 and 34 genes, named STG+OK and STG–OK respectively, which were considered the best candidates to be regulated by Sal and to mediate the functions of Sal proteins (Fig 1H; see Table 1).

**Table 1:** Best candidates Salm/Salr Target Genes (STG OK). Best candidates Spalt-repressed genes and Best candidates Spalt-activated genes with a Spalt-related phenotype.

***Best Candidates Spalt-repressed genes***						
**Name**	**Symbol**	**Array**	**Ph**	**IS**	**Molecular**	**MC**
*CG10916*	*CG10916*	Df/UAS	S-P	U+	Zinc ion binding	D
*CG10965*	*Corp*	24/48	S	N+	Response to DNA damage	CD
*CG11086*	*Gadd45*	24/48	+	N+	JNK cascade	CS
*CG11897*	*CG11897*	24/48	+	U+	ABC transporter	Tra
*CG1303*	*agt*	Df/UAS	+	U+	DNA methylation	D
*CG14121*	*ver*	Df/UAS	+	U+	Telomere capping	D
*CG14907*	*CG14907*	24/48	+	N+	--------------	CG
*CG15784*	*CG15784*	24/48	S-P	S+	--------------	CG
*CG16928*	*mre11*	Df/UAS	+	N+	Nucelase activity	D
*CG17104*	*CG17104*	24/48	+	SS+	--------------	CG
*CG17530*	*GstE6*	24/48	+	N+	Glutathione S-transferase	RedOx
*CG17533*	*GstE8*	24/48	S	U+	Glutathione transferase	RedOx
*CG18213*	*CG18213*	Df/UAS	+	N+	Zinc finger	CGh
*CG18455*	*Optix*	24/48	S-P	SS+	Sequence-specific DNA binding	D
*CG18522*	*CG18522*	24/48	+	N+	Oxidoreductase	RedOx
*CG1925*	*mus205*	Df	Bs//S	U+	DNA binding	D
*CG2909*	*CG2909*	24/48	V+	U+	--------------	CG
*CG2999*	*unc-13*	Df/UAS	+	S+	Intracellular signal transduction	CS
*CG3008*	*CG3008*	UAS	S-P	U+	Protein kinase activity	P
*CG3074*	*Swim*	24/48	+	U+	Wnt-protein binding	CS
*CG32021*	*CG32021*	Df/UAS	V-/S	N+	--------------	CG
*CG32625*	*CG32625*	24/48	+	U+	CHHC zinc finger	CGh
*CG33048*	*Mocs1*	24/48	--------	U+	Mo-molybdopterin cofactor	M
*CG3448*	*CG3448*	24/48	+	N+	DNA repair protein XRCC4	D
*CG43144*	*CG43144*	24/48	-------	N+	Non protein coding gene	CR
*CG45050*	*CG43674*	24	+	U+	DNA binding	D
*CG5096*	*CG5096*	24/48	+	U+	Leucine-rich repeat	CGh
*CG5202*	*escl*	UAS	+	U+	Histone methyltransferase	D
*CG5247*	*Irbp*	UAS	S-P	U+	DNA binding	D
*CG6272*	*CG6272*	Df/UAS	EPL	N+	Sequence-specific DNA bind	D
*CG6658*	*Ugt86Di*	24/48	+	SS+	Glucuronosyltransferase	M
*CG7590*	*scyl*	24/48	+	U+	Response to DNA damage	CD
***Best Candidates Spalt-activated genes with a Spalt-related phenotype***						
**Name**	**Symbol**	**Array**	**Ph**	**IS**	**Molecular**	**MC**
*CG11357*	*CG11357*	Df	Bs	U-	Transferring glycosyl groups	P
*CG11607*	*H2*.*0*	UAS	sS-P	U-	DNA binding transcription factor	D
*CG11883*	*CG11883*	UAS	S-P	U-	5'-nucleotidase activity	D
*CG12182*	*CG12182*	24/48	S-P	U-	--------------	CG
*CG12287*	*pdm2*	UAS	Bs	U-	DNA binding	D
*CG13083*	*CG13083*	24/48	S-P	U-	--------------	CG
*CG14394*	*NijC*	24/48	Bs/S/V+	U-	Ninjurin	CA
*CG15739*	*CG15739*	Df/UAS	S-P	U-	Phosphatase activity	P
*CG16705*	*SPE*	Df	S-P	U-	Serine-type endopeptidase	P
*CG16756*	*CG16756*	Df	S	U-	Lysozyme activity	P
*CG1725*	*dlg1*	Df	S-P	U-	EGFR binding	CS
*CG1869*	*Cht7*	Df	S-P	SS-	Chitinase activity	Cut
*CG30069*	*CG30069*	Df	S/Bs	SS-	--------------	CG
*CG31098*	*CG10634*	Df/UAS	V+	U-	Transferase activity	P
*CG31436*	*CG31436*	24/48	S/V-	U-	CHK kinase-like	CGh
*CG32029*	*Cpr66D*	Df/UAS	S/Bs	U-	Structural constituent of cuticle	Cut
*CG32055*	*CG32055*	Df/UAS	S	SS-	Insulin-like growth factor bind	CS
*CG32261*	*Gr64a*	UAS	S	U-	Taste receptor activity	CS
*CG33197*	*mbl*	Df/UAS	Bs	U-	DNA binding	D
*CG33302*	*Cpr31A*	24/48	S/Bs	U-	Structural constituent of cuticle	Cut
*CG42614*	*scrib*	UAS	S-P	U-	Protein binding	CS
*CG4322*	*moody*	UAS	S-P	U-	Melatonin receptor activity	CS
*CG4379*	*Pka-C1*	UAS	S-P	U-	Protein binding	CS
*CG4570*	*CG4570*	24/48	S	U-	Transposase, Tc1-like	D
*CG5249*	*Blimp-1*	UAS	S-P	U-	Nucleic acid binding	D
*CG7160*	*Cpr78E*	24/48	S	U-	Cuticle	Cut
*CG7577*	*ppk20*	Df/UAS	S	U-	Sodium channel activity	Tra
*CG7734*	*shn*	UAS	S-P	U-	DNA binding transcription factor	D
*CG8084*	*ana*	UAS	S-P	SS-	Growth factor activity	CS
*CG8675*	*CG8675*	Df	S-P	U-	--------------	CG
*CG8768*	*CG8768*	24/48	S	U-	NAD epimerase/dehydratase	M
*CG8780*	*tey*	UAS	S/Bs	U-	Regulation of transcription	D
*CG9333*	*Oseg5*	24/48	S/V+	U-	WD40-repeat	CGh
*CG9355*	*dy*	UAS	S-P	U-	Structural constituent of cuticle	Cut

“Array” indicates the experiment which the genes were selected, experiment 1 (24/48) or experiment 2 (Df//UAS). “Ph” indicates the phenotype of *nub-Gal4/UAS-RNA-i* and *sal* ^*EPv*^ *-Gal4/UAS-RNA-i* separated by double dash: + (WT); S (wing size); P (vein pattern); S-P (wing size and vein pattern); V+ and V- (extra-veins and loss of veins); Bs (dorso-ventral wing surface adhesion); EPL (early pupal lethal). “IS” indicates the mRNA expression patterns in wild type and *salm-i/salr-i* discs (N: expression not detected; U: generalised expression and P: patterned expression). We indicated by “+” or “-”that the expression appears ectopic or is reduced in *salmi*/*salri* discs, respectively. “Molecular” indicates the molecular nature and simplified molecular class (MC), respectively: D (genes related with the biology of DNA), P (genes related with proteins metabolism), CG (genes without known functional domains or orthology), CS (genes encoding components of signalling pathways), CGh (genes with a known functional domain but not clear orthology relationships), M (genes encoding proteins related to the metabolism of lipids or glucids), CA (genes related with cell adhesion or the cytoskeleton), Cut (genes encoding proteins related with structural constituent of cuticle), Tra (genes related with the transport of metabolites across cellular membranes), RedOx (genes encoding proteins related with oxidation-reduction process), CD (genes related with cell death) and CR (non protein coding gene).

### Gene Ontology analysis

The STG+ and STG- (286) genes encode proteins belonging to a variety of molecular categories among which stand out those related to the biology of DNA (17%) and proteins (11%), and general metabolism of lipids or glucids (9%; S2 Fig). They also include a large collection of genes without known functional domains or orthology relationships (18%) as well as genes with some known functional domain but not clear orthology (8%) (S2 Fig). The class of genes related to the biology of DNA includes all sequence-specific transcription factors and other DNA modifying enzymes that impinge in the regulation of gene expression, and its prominence suggests that Salm/Salr proteins might regulate wing development in part by controlling the expression of other transcription factors acting as intermediaries. Some sets were differently represented in the STG+ and STG- groups, including protein metabolism (6% versus 16%), Red-Ox (9% vs 2%) and cell signalling (7% vs 14%). The biological significance of these variations is at present unknown.

To obtain information about possible enrichments of gene functions related to the known requirements of *salm/salr*, we inspected several collections of genes using the software DAVID [28,29] with the GO term “biological process” (S4 Table). For those genes whose RNA expression levels change with a adjusted p-value lower than 0.05 at 24h after *salm/salr* RNAi induction (686 genes), we only found a limited enrichment of GO terms related with the known functions of Sal (S4 Table). In contrast, the STG genes with reduced expression in *salm/salr* knockdown (STG-; 147) were enriched for the terms “regulation of cell proliferation” (5 genes), “tissue development” (9 genes), “cell morphogenesis” (9 genes), “regulation of signal transduction” (8 genes) and “regulation of biosynthetic process” (15 genes) (Fig 1I and S4 Table). The STG genes with augmented expression in *salm/salr* knockdown conditions (STG+; 139 genes) were only enriched in the descriptions “regulation of gene expression” (13 genes) and “anatomical structure homeostasis” (3 genes) Fig 1I and S4 Table).

### mRNA expression in the wing disc of selected genes with increased expression in *salm-i/salr-i* compared to wild type wing discs

**Fig 2:**
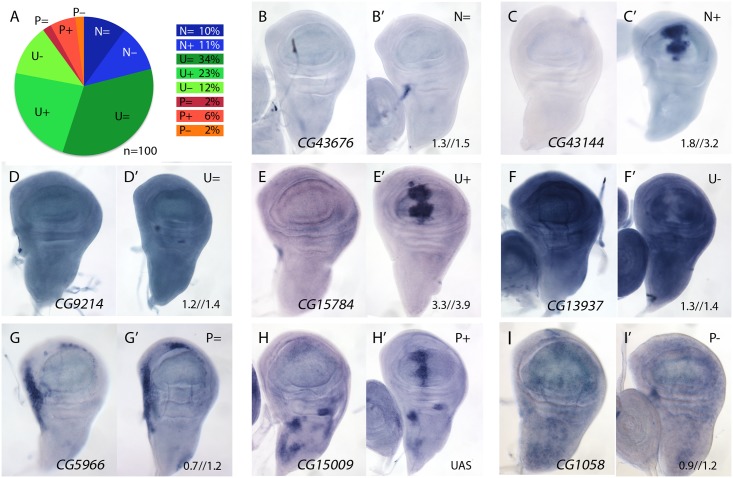
Examples of mRNA expression of selected genes (STG+) with increased expression in *salm-i/salr-i* compared to wild type wing discs. (A) Frequency of expression patterns encountered in wild type discs and observed changes in *UAS-dicer2/+; sal* ^*EPv*^ *-Gal4 UAS-GFP/UAS-salm-i; UAS-salr-i/+* wing discs. Expression not detected in the wing disc (N), generalised expression (U) and patterned expression (P). We indicated by “=“, “+” and “-”that the corresponding expression does not change, appears ectopic or is reduced in *salm-i/salr-i* wing discs, respectively. (B-I) Representative examples of *in situ* hybridizations in late third instar wing discs with probes against the genes *CG43676* (N =; B-B’), *CG43144* (N =; C-C’), *CG9214* (U =; D-D’), *CG15784* (U+; E-E’), *CG13937* (U-; F-F’), *CG5966* (P =; G-G’), *CG15009* (P+; H-H’) and *CG1058* (P-; I-I’). In each pair of panels, B-I corresponds to wild type discs and B’-I’ to UAS-*dicer2/+; sal* ^*EPv*^ *-Gal4 UAS-GFP/UAS-salm-i; UAS-salr-i/+* wing discs. The expression patterns class and logFC values at 24h and 48 h from experiment 1 or the microarray condition from experiment 2 are indicated at the top and to the bottom corner, respectively, of each panel in B’ to L’ images.

We studied the expression pattern of 100 of the 139 STG+ genes in wild type and in *salm-i/salr-i* backgrounds. Genes that were identified only as changing at T48 (39) were discarded for this analysis, as we assumed that, likely, this group included most genes whose expression changes indirectly in response to the loss of *salm/salr* function. Most of the analysed genes (n = 100) displayed a generalised pattern of expression in third instar wing disc (U, 69%; Fig 2A and examples in Fig 2D–2F) or their expression was not detected by in situ hybridization (N, 21%; Fig 2A and examples in Fig 2B and 2C). Only 10% of the genes analysed were expressed with a spatial pattern that, in general, was unrelated to the pattern of expression of *salm/salr* in the wing blade (P; Fig 2A and examples in Fig 2G–2I). The expression patterns of all genes included in these categories are shown in S3–S7 Figs, and selected representative examples are shown in Fig 2B–2I.

The expression of all these genes was also studied in *salm/salr* loss of function conditions (*UAS-dicer2/+; sal* ^*EPv*^ *-Gal4 UAS-GFP/UAS-salm-i; UAS-salr-i/+*). We found a significant change in the expression patterns detected by *in situ* hybridization in 40 cases (40%). In all these cases, and irrespectively of the normal pattern of expression of the gene, we detected a strong stripe of increased (for U and P genes) or ectopic (for N genes) expression localised in the central region of the wing disc (Fig 2C-2C’, 2E-2E’ and 2H-2H’; see also S3 and S4 Figs). For the remnant of genes (n = 60) either we could not detect any change in their spatial expression (46%; Fig 2A, 2B-2B’, 2D-2D’ and 2G-2G’) or their expressions were reduced (14%; Fig 2A, 2F-2F’ and 2I-2I’). We do not know the reasons underlying this discrepancy between the microarray data and the *in situ* hybridization analysis. However, as both techniques as subject to different sources of noise and experimental error, we considered that this discrepancy was acceptable, and focussed for any subsequent analysis in those genes that display a clear change by *in situ* hybridization in *salm-i/salr-i* knockdown discs. All cases in which we found an increase in expression in *salm/salr* knockdown discs are shown in S3 and S4 Figs, and those for which we could not detect a change in the *in situ* expression or their expressions were reduced are shown in S5–S7 Figs.

Interestingly, the genomic regions associated to the genes over-expressed in *salm/salr* knock-down discs (n = 40) were enriched in the H3K9me3 epigenetic mark (12,79% versus 3,09% in the genome, p-value 0.001), which is associated to heterochromatic regions [30]. These genes included an enriched set for the terms “DNA metabolic process”, “telomere organization” and “cellular response to DNA damage stimulus” formed by *mre11*, *mus205*, *Irbp*, *agt*, *Dif* and *scyl* (see Fig 1J and S4 Table, STG+OK). The functional relation between these genes and the Sal functions is unknown, but certainly deserves an in depth exploration.

### A fraction of genes over-expressed in *salm/salr* knockdown background are targets of the JNK pathway

**Fig 3:**
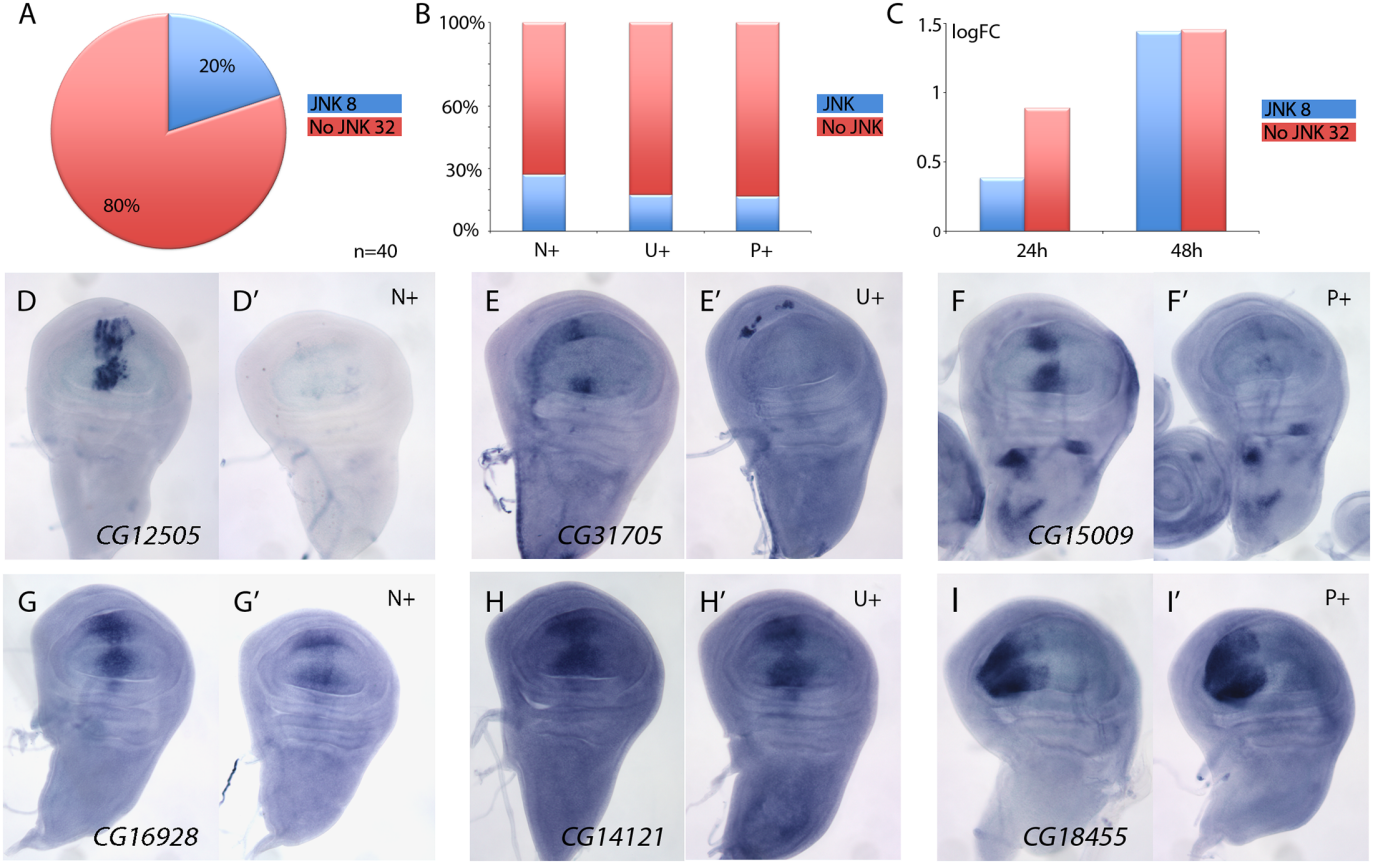
Analysis of JNK-dependent gene regulation for genes ectopically expressed in *salm/salr* knockdown discs. (A) Frequency of genes which ectopic expression in *salm-i/salr-i* discs is independent (80%, red; 32 genes) or dependent (20%, blue; 8 genes) of JNK signalling. (B) Percentage of JNK-dependent genes (blue) and JNK-independent genes (red) which are normally not expressed (N+), are expressed in a generalised manner (U+) or are expressed with a spatially restricted pattern (P+). (C) Average of logFC of JNK-dependent genes (blue columns) and JNK-independent genes (red columns) at 24h (left columns) and 48h (right columns) at 29°C. (D-F’) Representative examples of *in situ* hybridizations in late third instar wing discs with probes against the JNK-dependent genes *CG12505* (D-D’), *CG31705* (E-E’) and *CG15009* (F-F’). The panels D-F correspond to *sal* ^*EPv*^ *-Gal4 UAS-GFP/UAS-salm-i; UAS-salr-i/UAS-GFP* discs and D’-F’ to *sal* ^*EPv*^ *-Gal4 UAS-GFP/UAS-salm-i; UAS-salr-i/UAS-puc* wing discs. (G-I’) Representative examples of *in situ* hybridizations in late third instar wing discs with probes against the JNK-independent genes *CG16928* (G-G’), *CG14121* (H-H’), and *CG18455* (I-I’). The panels G-I correspond to *sal* ^*EPv*^ *-Gal4 UAS-GFP/UAS-salm-i; UAS-salr-i/UAS-GFP* discs and G’-I’ to—*sal* ^*EPv*^ *-Gal4 UAS-GFP/UAS-salm-i; UAS-salr-i/UAS-puc* wing discs.

The JNK signalling pathway is activated in the Salm/Salr domain in *salm/salr* knockdown backgrounds [8]. This pathway can regulate gene expression through the action of the JUN and FOS transcription factors [31], and therefore, it is possible that some of the genes over-expressed in the central domain of *salm/salr* knockdown wing discs correspond to targets of the JNK pathway. To distinguish between genes repressed by Salm/Salr and genes activated by JNK signalling, we studied the expression of genes over-expressed in *salm/salr* knockdown background (n = 40) when JNK signalling is suppressed. To suppress this pathway, we over-expressed the JNK phosphatase *puckered* (*puc*) in wing discs where *salm* and *salr* expression was reduced by RNA interference. We found that the suppression of JNK by *puc* over-expression cancels the ectopic expression observed in *salmi/salri* background for 8 genes (Fig 3A, 3D–3F’ and S8 Fig). These genes were more frequently either not expressed in wild type discs (N+; 3 out of 11, see Fig 3B) or expressed in a generalised manner (U+; 4 out of 23, see Fig 3B). Only the ectopic expression of one gene that was normally expressed in a spatially restricted pattern turned out to be regulated by, or dependent on, JNK signalling (P+; 1 out of 6, see Fig 3B). Interestingly, most JNK-dependent genes were either undetected or detected with low logFC values after the 24h period at 29°C (Fig 3C), which is compatible with them being regulated by Salm/Salr indirectly. For 32 out of 40 genes analysed, we found that the over-expression of *puc* did not affect the characteristic ectopic expression caused by the loss of *salm/salr* (Fig 3A and 3G–3I’ and S8 Fig). Most genes whose expression behaves as independent of JNK displayed a significant logFC at both 24 and 48 hours intervals, which suggest that they might be direct targets of Salm/Salr regulation (Fig 3C). This set of 34 genes that show JNK-independent over-expression in *salmi/salri* discs was considered the collection of the best candidate Sal target genes regulated by repression (STG+OK; Fig 1G and 1J and Table 1).

It is possible that some of the phenotypic aspects of loss of Sal function are related with the ectopic expression of genes showing a strong de-repression in the central domain of the wing pouch in *salm/salr* knockdown conditions. To identify whether this was the case, we introduced RNA interference for all 40 genes de-repressed in the *salm-i/salr-i* background. In none of these combinations (*sal* ^*EPv*^ *-Gal4 UAS-salm-i/UAS-RNA-i; UAS-salr-i/+*) we could detect a significant change of the phenotype compared with control flies (*sal* ^*EPv*^ *-Gal4 UAS-salm-i/UAS-GFP; UAS-salr-i/+*) indicating that the phenotype of *salm/salr* cannot be assigned to single genes being over-expressed.

### mRNA expression in the wing disc of selected genes with decreased expression in *salmi/salri* compared to wild type wing discs

**Fig 4:**
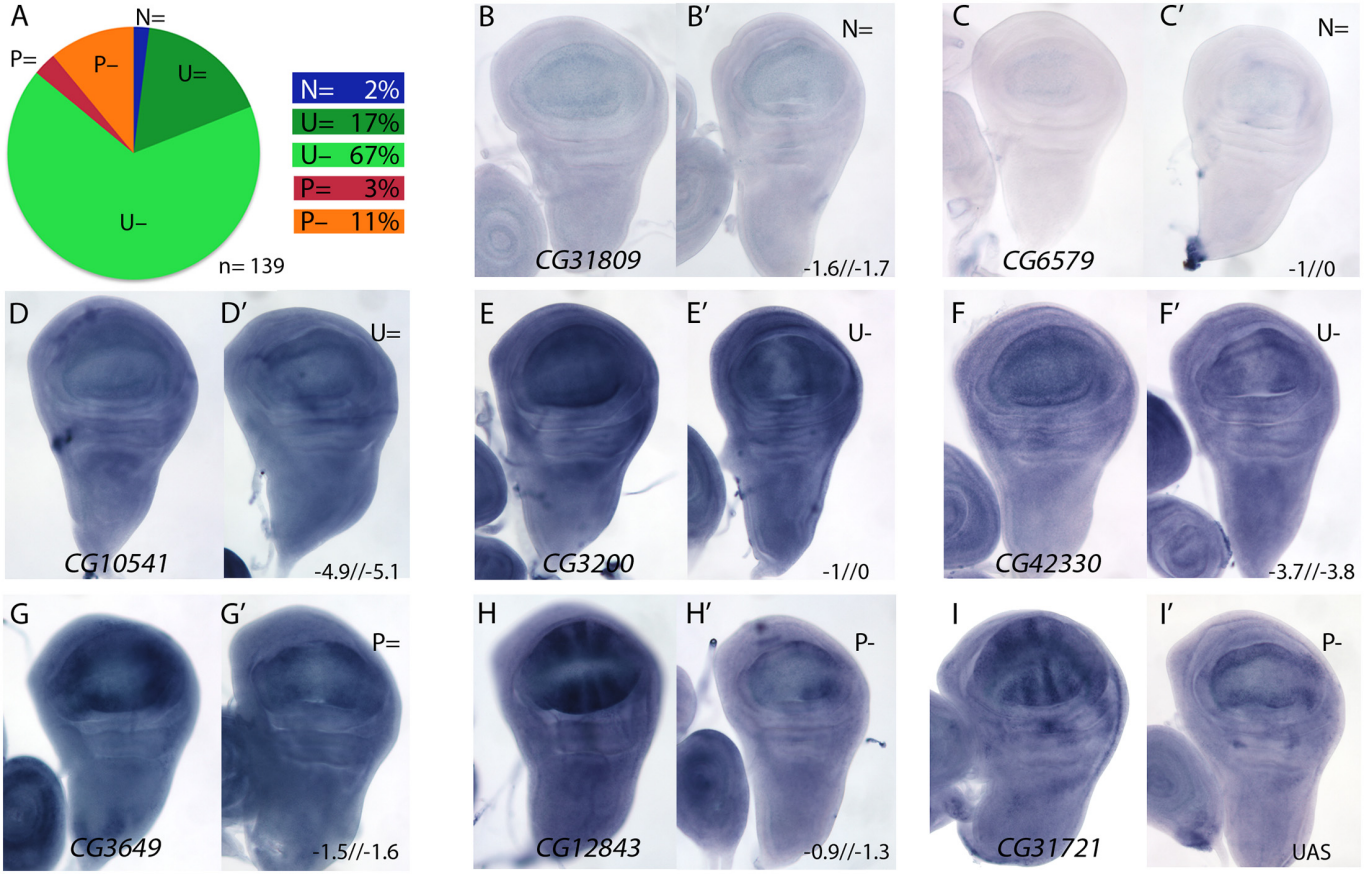
Examples of mRNA expression of selected genes with reduced expression in *salm-i/salr-i* compared to wild type wing discs. (A) Frequency of expression patterns in wild type discs and observed changes in *UAS-dicer2/+; sal* ^*EPv*^ *-Gal4 UAS-GFP/UAS-salm-i; UAS-salr-i/+* wing discs. Expression not detected in the wing disc (N), generalised expression (U) and patterned expression (P). We indicated by “=“, “+” and “-”that the expression does not change, appears ectopic or is reduced in *salmi*/*salri* discs, respectively. (B-I) Representative examples of *in situ* hybridizations in late third instar wing discs with probes against the genes *CG31809* (N =; B-B’), *CG6579* (N =; C-C’), *CG10541* (U =; D-D’), *CG3200* (U-; E-E’), CG42330 (U-; F-F’), *CG3649* (P =; G-G’), *CG12843* (P-; H-H’) and *CG31721* (P-; I-I’). In each pair of panels, B-I corresponds to wild type discs and B’-I’ to *UAS-dicer2/+; sal* ^*EPv*^ *-Gal4 UAS-GFP/UAS-salm-i; UAS-salr-i/+* wing discs. The expression patterns class and logFC values at 24h and 48 h from experiment 1 or the microarray condition from experiment 2 are indicated at the top and to the bottom corner, respectively, of each B’ to L’ panel.

We also selected 147 genes whose expression was reduced in *salm/salr* knockdown discs (STG-; Fig 1H), and studied the expression pattern of 139 of them. Most of these genes are expressed in a generalised manner in the wing disc (84%; class U, Fig 4A), and only 14% displayed restricted expression patterns that relate to the formation of veins and interveins (Fig 4A; class P). For 2% of the selected genes we could not detect expression by *in situ* hybridization in the wing disc (Fig 4A–4C’; class N). The changes observed in the expression of genes detected as reduced in *salmi/salri* background were very coherent, and consisted in a strong reduction of their expression in the central region of the wing pouch (Fig 4E–4F’, 4H–4I’, S9–S12 Figs). This change was observed for genes that are normally expressed in all wing disc cells (class U; Fig 4E–4F’ and S9, S11 and S12 Figs), and for genes displaying restricted expression patterns (class P; Fig 4H–4I’ and S9 and S10 Figs).

**Fig 5:**
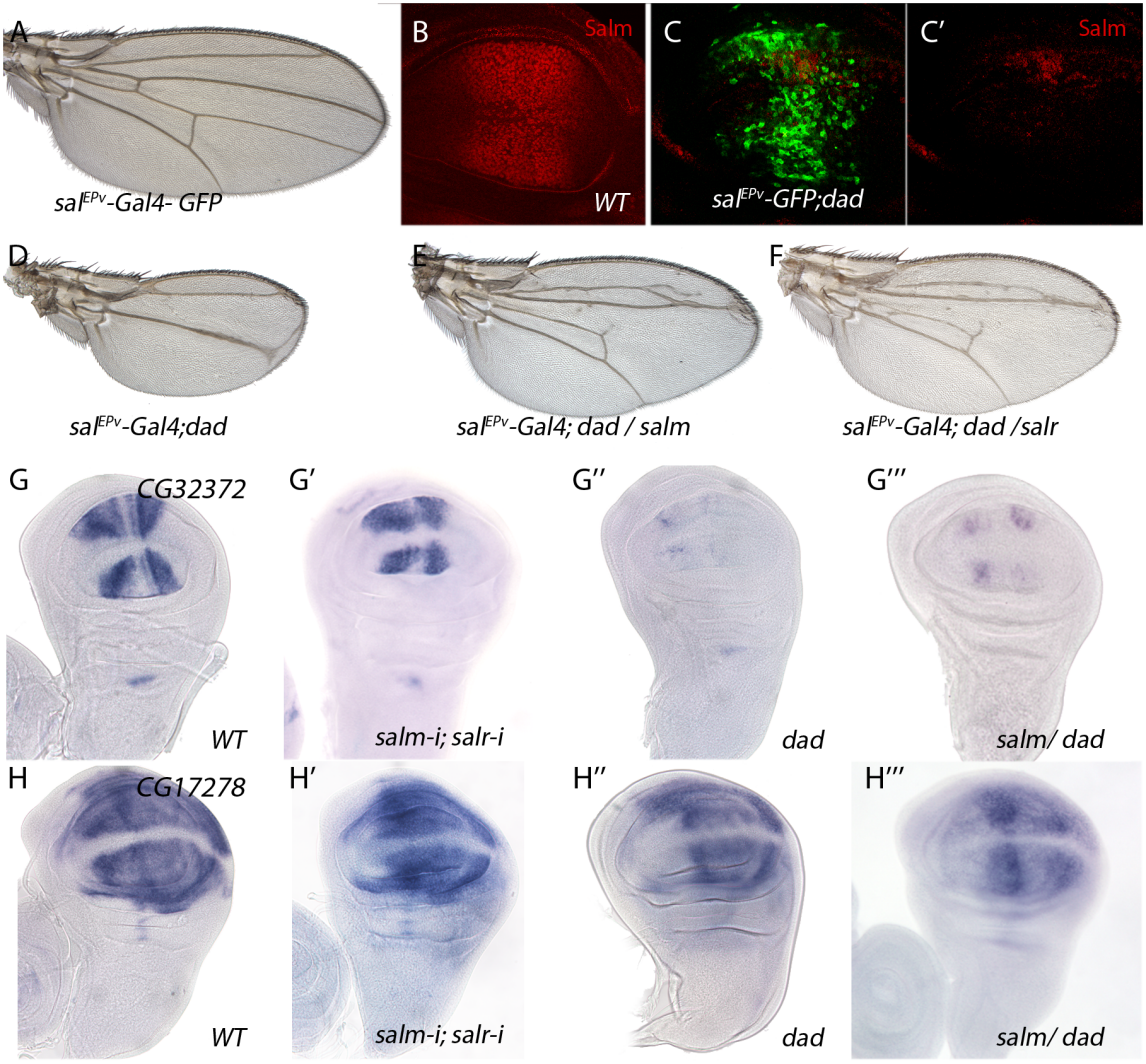
Genes regulated by Dpp signalling independently of Salm/Salr. (A) Control *sal* ^*EPv*^ *-Gal4 UAS-GFP/+* wing. (B) Control third instar wing disc showing Spalt major expression (Red). (C-C’) *sal* ^*EPv*^ *-Gal4 UAS-GFP/+; UAS-dad/+* wing disc showing Salm (red in C and C’) and GFP expression (green in C). (D) *sal* ^*EPv*^ *-Gal4 UAS-GFP/+; UAS-dad/+* wing. (E) *sal* ^*EPv*^ *-Gal4 UAS-GFP/+; UAS-dad/UAS-salm* wing. (F) *sal* ^*EPv*^ *-Gal4 UAS-GFP/+; UAS-dad/UAS-salr* wing. Forced expression of Salm (E) or Salr (F) in the central region of *sal* ^*EPv*^ *-Gal4 UAS-GFP;UAS-dad* (D) discs partially rescues the size and pattern defects caused by Dad over-expression. (G-G’’’) *CG32372 in situ* hybridization in third instar discs of *sal* ^*EPv*^ *-Gal4 UAS-GFP/+* (G), *sal* ^*EPv*^ *-Gal4 UAS-GFP/UAS-salm-i; UAS-salr-i/+* (G’), *sal* ^*EPv*^ *-Gal4 UAS-GFP/+; UAS-dad/+* (G’’) and *sal* ^*EPv*^ *-Gal4 UAS-GFP/+; UAS-dad/UAS-salm* (G’’’). (H-H’’’) *CG17278 in situ* hybridization in third instar discs of *sal* ^*EPv*^ *-Gal4 UAS-GFP/+* (H), *sal* ^*EPv*^ *-Gal4 UAS-GFP/UAS-salm-i; UAS-salr-i/+* (H’), *sal* ^*EPv*^ *-Gal4 UAS-GFP/+; UAS-dad/+* (H’’) and *sal* ^*EPv*^ *-Gal4 UAS-GFP/+; UAS-dad/UAS-salm* (H’’’). The over-expression of Salm does not rescue the loss of *CG32372* or *CG17278* expression caused by increased Dad expression.

For a 22% of the analysed cases we could not detect a change in the expression pattern comparing wild type and *salm/salr* knockdown discs (Fig 4A, Fig 4D-4D’, Fig 4G-4G’ and S13 Fig). We wondered whether some of these genes were regulated by the Dpp pathway, independently of Spalt, in the central region of the wing. To reduce Dpp activity we over-expressed Dad in this territory (*sal* ^*EPv*^ *-Gal4; UAS-dad*; Fig 5A–5D). In this background the expression of Sal was strongly reduced (Fig 5C-5C’; compared with Fig 5B) and the resulting adult wing display a moderate to strong Dpp phenotype (Fig 5D), that could be partially rescued by the co-expression of either Salm (Fig 5E) or Salr (Fig 5F). Indeed, we found that the expression of *CG32372* (Fig 5G-5G’’’) and *CG17278* (Fig 5H-5H’’’) are not strongly affected in *salm-i/salr-i* discs (Fig 5G’, 5H’ and 5I’), but are reduced or absent in *UAS-dad* discs (Fig 5G’’ and 5H’’). Because the loss of *CG32372 and CG17278* in *UAS-dad* discs cannot be rescued by the over-expression of Salm (Fig 5G’’’ and 5H’’’), we suggest that these genes are likely candidates for Dpp regulation independently of Salm/Salr. Indeed, *CG32372* corresponds to *larval translucida*, a gene which expression was already shown to be regulated by Dpp [32]. We assume that genes expressed in the central domain of the wing disc and regulated by Dpp will be picked up in our microarray experiments as Sal-, because the extent of this territory is reduced in *salm/salr* knockdown discs.

### Phenotypic analysis of genes with modified level of expression in *salm/salr* knockdown discs

**Fig 6:**
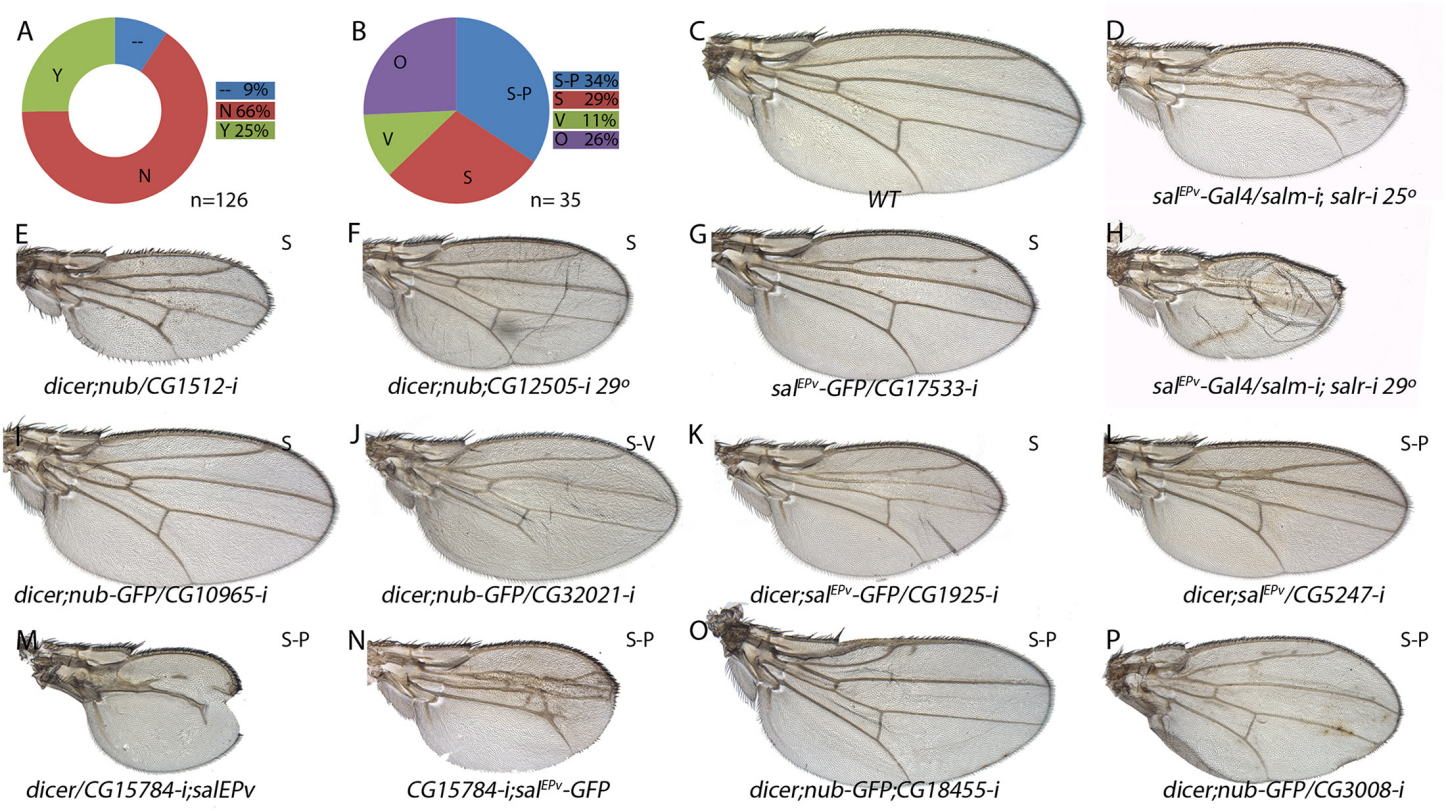
Phenotypic analysis of genes with increased expression in *sal* knockdown discs. (A) Fraction of genes showing a wing mutant phenotype (Y; green) and no phenotype (N; red) from a total of 126 *UAS-dicer2/+; nub-Gal4/ UAS-RNA-i* or *UAS-dicer2/+; sal* ^*EPv*^ *-Gal4/ UAS-RNA-i* combinations. Genes not analysed from the STG+ class are shown in blue (—) (B) Frequency of defects in both wing size and vein pattern (S-P; blue), changes in wing size (S; red), defects in vein formation with minor or no effect in wing size (V; green) and other wing morphology defects (O; purple) from a total of 35 *UAS-RNA-i/Gal4* combinations. (C) Wild type wing. (E-F) Representative examples of adult wings knockdown for genes over-expressed in *salm-i/salr-i* mutant discs but activated in response to JNK. *UAS-dicer2/+; nub-Gal4/UAS-CG1512-i* (E) and UAS-*dicer2/+; nub-Gal4/UAS-CG12505-i* (F). (G-P) Representative examples of adult wings of *UAS-RNA-i/Gal4* combinations for genes over-expressed in *salm-i/salr-i* knockdown discs independently of JNK activity: *sal* ^*EPv*^ *-Gal4 UAS-GFP/UAS-CG17533-i* (G), *UAS-dicer2/+; nub-Gal4/UAS-CG10965-i* (I), *UAS-dicer2/+; nub-Gal4/UAS-CG32021-i* (J), *UAS-dicer2/+; sal* ^*EPv*^ *-Gal4/UAS-CG1925-i* (K), */+;UAS-dicer2/+; sal* ^*EPv*^ *-Gal4/ UAS-CG5247-i* (L), *UAS-dicer2/UAS-CG15784-i; sal* ^*EPv*^ *-Gal4/+* (M), *UAS-CG15784-i; sal* ^*EPv*^ *-Gal4 UAS-GFP/+* (N), *UAS-dicer2/+; sal* ^*EPv*^ *-Gal4/UAS-CG18455-i* (O) and *UAS-dicer2/+; nub-Gal4/UAS-CG3008-i* (P). Moderate and strong *salm-i/salr-i* knockdown phenotypes are shown for comparison in (D) and (H), in wings of *sal* ^*EPv*^ *-Gal4/ UAS-salm-i; UAS-salr-i/+* raised at 25°C and 29°C, respectively.

**Fig 7:**
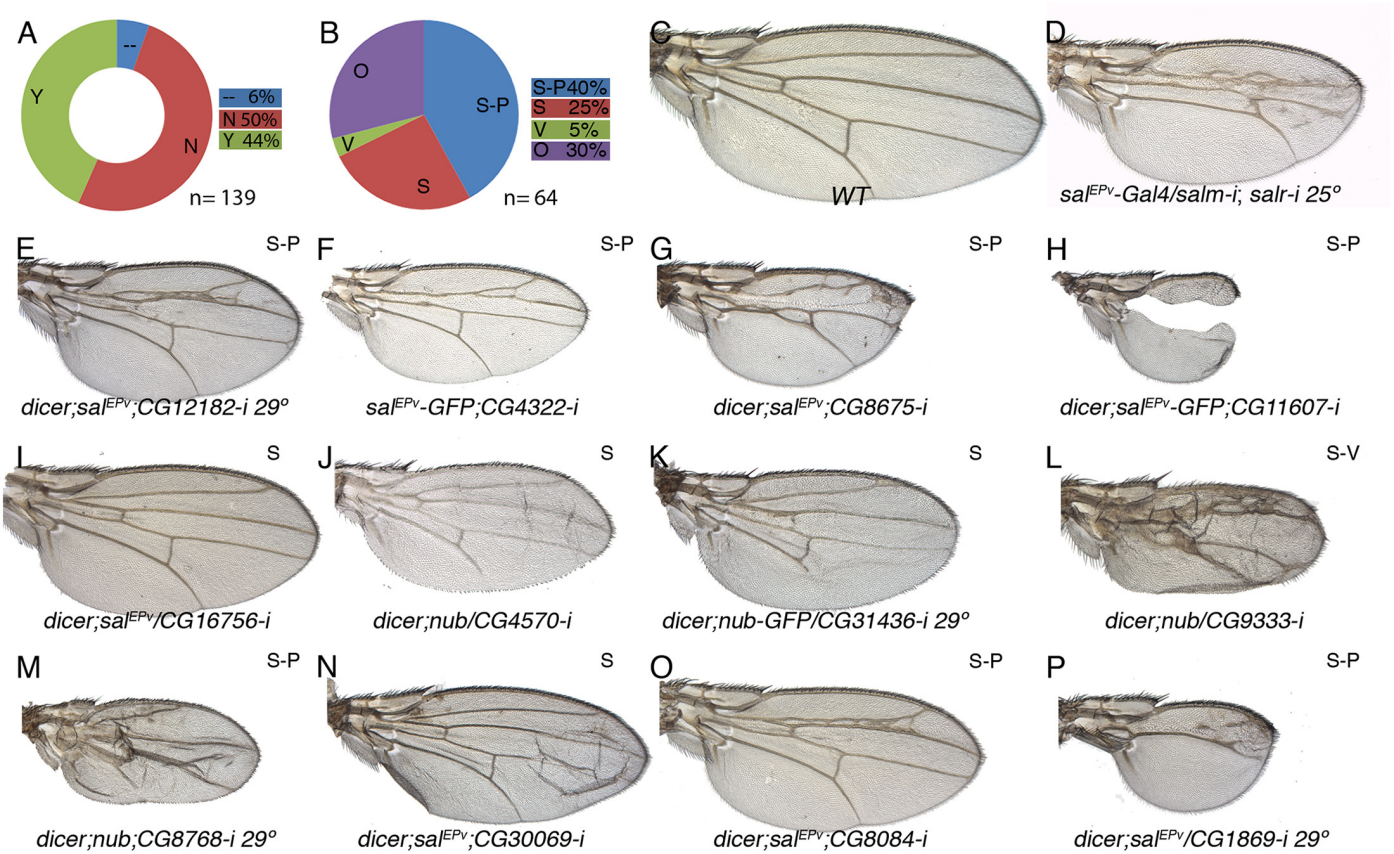
Phenotypic analysis of genes with reduced expression in *sal* knockdown discs. (A) Fraction of genes showing a wing mutant phenotype (Y; green) and no phenotype (N; red) from a total of 139 *UAS-RNA-i/Gal4* combinations. Genes not analysed of the STG- class are shown in blue (—). (B) Frequency of mutant wings showing defects in both wing size and vein pattern (blue; S-P), changes in wing size (S; red), defects in vein formation with minor or no effect in wing size (V; green) and other morphological defects in the wing (O; purple) from a total of 64 *UAS-RNA-i/Gal4* combinations. (C) Wild type wing. (D) *sal* ^*EPv*^ *-Gal4/UAS-salm-i; UAS-salr-i/+* wing. (E-P) Representative examples of adult wings knockdown for genes showing reduced expression by in situ hybridization in *salm-i/salr-i* wing discs. *UAS-dicer2/+; sal* ^*EPv*^ *-Gal4/UAS-CG12182-i* grown at 29°C (E); *sal* ^*EPv*^ *-Gal4 UAS-GFP/UAS-CG4322-i* (F), *UAS-dicer2/+; sal* ^*EPv*^ *-Gal4 / UAS-CG8675-i* (G), *UAS-dicer2/+; sal* ^*EPv*^ *-Gal4/+; UAS-CG11607-i/+* (G), *dicer2/+; sal* ^*EPv*^ *-Gal4/ UAS-CG16756-i* (I), *UAS-dicer2/+; nub-Gal4/UAS-CG4570-i* (J), *UAS-dicer2/+; nub-Gal4/UAS-CG31436-i* (K), *UAS-dicer2/+; nub-Gal4/+; UAS-CG9333-i/+* (L), *UAS-dicer2/+; nub-Gal4/UAS-CG8768-i* (M), *UAS-dicer2/+; sal* ^*EPv*^ *-Gal4/UAS-CG30069-i* (N), *UAS-dicer2/+; sal* ^*EPv*^ *-Gal4/+; UAS-CG8084-i/+* (O) and *UAS-dicer2/+; sal* ^*EPv*^ *-Gal4/ UAS-CG1869-i* at 29°C (P).

From the microarray and *in situ* hybridization data, it seems clear that the reduction of *salm/salr* function in the wing disc causes a profound alteration in the transcriptional landscape of the wing. Some of these changes might be direct, but it is likely that many others could be the consequence of inappropriate expression of Salm/Salr target genes encoding transcription factors, or, as we found for JNK target genes, the result of inappropriate activation of signalling pathways. For these reasons, it is unlikely that by interfering with the expression of each individual gene we were able to reproduce the effects of *salm/salr* loss during wing development. Despite of this, we wanted to make a preliminary identification of the functional requirements of the Salm/Salr candidate downstream genes by analysing the consequences of reducing their expression. To this end, we expressed in the wing disc (*nub-Gal4/UAS-RNA-i* and *sal* ^*EPv*^ *-Gal4/UAS-RNA-i*) RNA interference directed against most genes belonging to the STG+ (126 out of 139) and STG- (139 out of 147) classes. As expected, we found that 66% of STG+ genes and 50% of STG- genes tested in *Gal4/UAS-RNAi* combinations resulted in wings that were normal in size and vein pattern (Figs 6A and 7A; class N and S5 Table). The remnant of the *Gal4/UAS-RNA-i* combinations resulted in wings with abnormal size, altered pattern of veins, or a collapse of the entire central region of the wing (Figs 6A and 6B, 7A and 7B; class Y and S5 Table).

Some combinations between *UAS-RNAi* and *nub-Gal4*, which is expressed in the wing blade and hinge, resulted in pupal lethality (9 for STG+ and 14 for STG-; S5 Table). In these cases, and when we were able to inspect the pharate adults, the phenotype was the total absence of the wing (see for example S14B Fig and S15F Fig). A similar no-wing phenotype was also found in some combinations (8) that were able to produce adult escapers (S5 Table). The *UAS-RNA-i* for all these genes (pupal lethal and no-wing phenotype in S5 Table) were also combined with *sal* ^*EPv*^ *-Gal4*, which is only expressed in the central region of the wing blade. In all cases, the resulting adult wings displayed a variety of phenotypes ranging from severe disruptions of the central wing territory (see for example Fig 6M) to subtle defects in the size and patterning of the wing (see for example Fig 6L). In addition to wings displaying both size and pattern defects (class S-P; 34% and 40% of viable combinations with a wing phenotype in the STG+ and STG- groups; Figs 6B, 6I–6P, 7B, 7E–7H, 7O and 7P), we also found a consistent phenotype in which the size of the wing was reduced, but the vein pattern remained intact (class S; 29% in the STG+ and 25% in the STG- groups; Figs 6B, 6E–6G, 6I–6K, 7B, 7I and 7J). With lesser frequencies we also found phenotypes in which the differentiation of the veins was disrupted, including cases with loss of veins or extra-veins (Figs 6B and 6J, 7B and 7L), and other phenotypes including failures in the adhesion of the dorsal and ventral wing surfaces (Fig 7N) and loss of wing margin structures. A representative collection of at least one genetic combination with the *nub-Gal4* or *sal* ^*EPv*^ *-Gal4* drivers is shown in S14 and S15 Figs.

It is somehow surprising that the distribution of loss-of-function phenotypes is similar for genes that were identified as activated or repressed by Salm/Salr. However, the majority of genes displaying over-expression in *salmi/salri* discs independent of JNK display a wild type loss-of-function phenotype (21 out of 32), as expected if they contribute to the *sal* phenotype by over-expression. For the set of genes which expression in the central domain of the wing is activated by Salm/Salr, we focused in those showing phenotype related to Salm/Salr requirements, as they are the most attractive candidates to mediate the functions of Sal proteins during wing development (34 genes). This group was named STG-OK (see Fig 1G and Table 1), and the genes belonging to it display mostly size and pattern (16/34) and size (14/34) defects, and they are enriched for the GO terms “cell proliferation”, “organ development” and “behavioural response to ethanol” (see Fig 1J and S4 Table, STG-OK).

## Discussion

The Spalt transcription factors occupy a central position in the genetic hierarchy linking Dpp signalling with the development of the Drosophila wing. Thus, this pathway directly regulates *salm/salr* expression in the central region of the wing blade, and these genes mediate most of the developmental roles of Dpp in this territory. In this manner, a necessary step to understand how Dpp signalling regulates wing development is to identify Spalt target genes that implement the variety of cellular responses elicited by the pathway. Here we found a complex transformation in the transcriptional landscape of *salm/salr* knockdown discs, and identify a collection of genes that might fulfil the criteria to be considered bona fide Spalt target genes. From these genes it should be possible to identify which of them are directly related with the known functions of the Dpp pathway in the wing.

### The microarray experiments and the selection criteria

**Fig 8:**
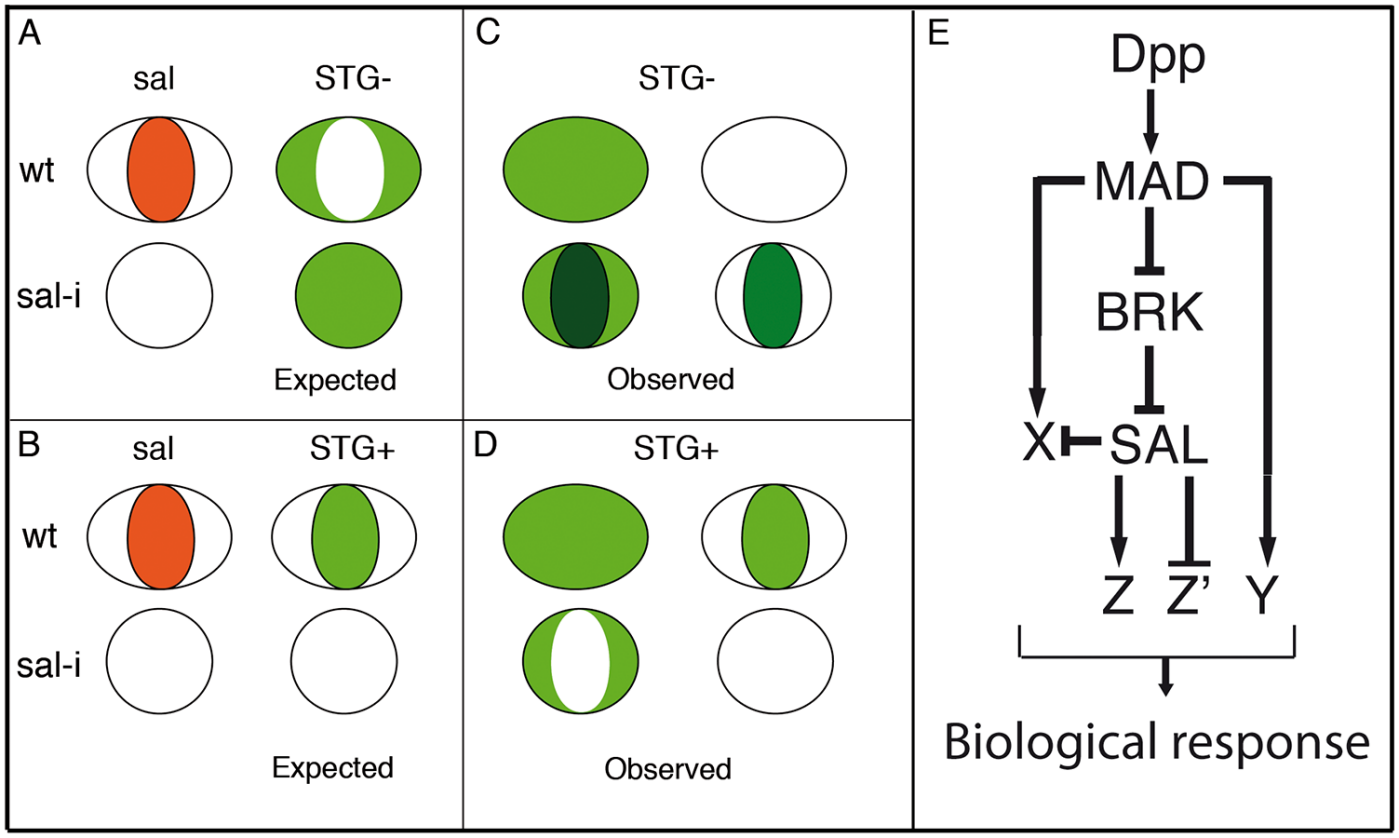
Gene regulatory network triggered by Dpp. (A-D) Schematic representation of the wing pouch (ovals and circles) showing the expected (green fill in A-B) and observed (green fill in C-D) expression in wild type discs (ovals) and in *salmi/salri* mutant discs (circles). The behaviour of genes repressed by Sal is shown in A (expected) and C (observed), and for genes activated by Sal in B (expected) and D (observed). (E) The expression of Salm/Salr (SAL) is regulated by the Dpp pathway components Brinker (BRK) and Mad (MAD). Dpp, using Mad or Mad and Brk, also activate the expression of additional genes (X and Y) which in turn can be subject to further regulation by Salm/Salr (X) or independent of them (Y). Finally, the Salm/Salr transcription factors regulate by activation (Z) or repression (Z’) the expression of their direct target genes, contributing to the set of active genes in the centre of the wing blade that confer this territory its particular characteristics.

Our identification of candidate Spalt target genes is based on microarrays experiments in which we compared genome wide gene expression levels in genetic backgrounds with altered *salm*/*salr* function. The simplest assumption is that the expression of a gene that is activated or repressed by Salm/Salr would be reduced or increased, respectively, in a *salm*/*salr* knockdown background (Fig 8A and 8B). The ability to identify such changes in the wing disc is somehow complicated, because we are only modifying *salm*/*salr* expression in the central region of the wing blade, which is a small region of a complex structure such as the wing disc. In addition, the changes we identified in expression levels could be attributed to a variety of causes unrelated to direct regulation by Salm/Salr. For example, loss of *salm*/*salr* function interferes with the growth of the disc, changing the size-proportions of the wing versus the rest of the disc. For this reason, we expect that a fraction of genes we are selecting might correspond to those that are differentially expressed in the wing blade versus the rest of the disc [25]. Furthermore, we know that some genes regulated, directly or indirectly, by Salm/Salr are expressed only in a very restricted population of cells, which most likely would difficult their identification using mRNA extracted from the entire disc for the microarray experiments. This is the case of the *Iroquois* and *knirps* gene complexes, which are expressed only in the developing veins L3/L5 and L2, respectively, in the wing blade [20] and that were not identified in the microarray experiments. In addition, and inherent to all microarray-based experiments, changes in levels of expression as a consequence of the loss or gain of *salm/salr* function do not necessarily indicate a direct regulation of the affected gene. Despite these caveats, we expect that at least some direct targets of Salm/Salr would be included within the population of genes showing a significant change in expression levels in *salm/salr* knockdown discs.

The *salm/salr* genes are expressed in a restricted region of the wing blade, and for this reason we decided to visualize directly the expression pattern of the genes identified in the microarrays by *in situ* hybridization. Since we identified a large number of genes in the microarrays (1653), we needed to restrict the expression analysis only to a fraction of this gene pool. The criteria we used to shortlist the genes for *in situ* hybridization experiments was based in the logFC values, prioritizing in some cases those genes that change early in response to the reduction in *salm/salr* expression. Setting a logFC value of 1 as threshold (2x fold change difference), we ended up with a short list of 286 candidate genes (STG), and analysed most of them by *in situ* hybridization in wild type and in *salm/salr* knockdown discs.

### Expression patterns of candidate Salm/Salr target genes

The expression patterns of the selected genes fall into three classes: first, a fraction of genes are expressed in a restricted pattern, which in some cases is related to the expression of Salm/Salr. Some of the genes expressed in the central region of the wing pouch, such as *CG1342* and *CG7201* behave as positive targets of Salm/Salr (Fig 4H and 4I’; Z in Fig 8E), whereas genes expressed in a complementary pattern, for example *CG2999* (S4O-S4O’ Fig), behave as negative targets of Salm/Salr (Z’ in Fig 8). In other cases (*CG32372* and *CG17278*) we were able to assign them to direct positive regulation by Dpp without a requirement for Salm/Salr function (Y in Fig 8E). The category of genes with restricted expression in the wing disc includes several whose expressions are related to the subdivision of the wing blade into pro-vein and inter-vein territories. The expression of these genes (*CG1273* and *CG33970* in the veins (S10A-S10A’ and S10H-S10H’ Fig) and *CG12843*, *CG18657* and *CG30069* in the interveins (Fig 4H-4H’ and S10B-S10B’ Fig and S10D-S10D’ Fig) are regulated by Salm/Salr only in the central region of the wing pouch, suggesting that they contain complex regulatory regions conferring them independent regulation in the central versus the most anterior and posterior regions of the developing wing blade.

A second class includes all the genes that are expressed in a generalised manner in the wing pouch or in the entire wing disc (Fig 8C and 8D). For many of these genes we found that Salm/Salr positively regulate their expression in the central region of the wing pouch (Fig 8D). Finally, we found a third class of genes that display a robust ectopic expression in the central region of the wing in response to the loss of *salm*/*salr* function (Fig 8C). Most of these genes are either not expressed at all in the wing disc, or are expressed in a generalised manner. All 40 genes included in this group show a consistent behaviour as negative targets of Salm/Salr, and they uncover an unexpected aspect of the genetic regulatory structure of the wing disc. Thus, it seems that one key contributions of Salm/Salr function to wing development is to prevent the deployment of a particular program of gene activation that is operative in the central region of the wing disc (X in Fig 8E). Because this territory coincides with the domain of Dpp signalling, it is likely that the pathway activates a battery of genes in the central domain of the wing disc whose expression is repressed by Salm/Salr, which are also Dpp targets (X in Fig 8E). This double regulatory mechanism consisting in direct activation and indirect repression triggered by the Dpp pathway might be very important to ensure that only a selected fraction of possible target genes are expressed in a particular tissue in response to the pathway. One important result of such dual positive/negative mode of regulation is to confer versatility to the genetic response to the pathway, refining the inventory of target genes that are activated in each developmental context.

We were able to further dissect the group of genes behaving as negative targets of Salm/Salr into two classes based on their dependence on JNK activity. Thus, we found that some of these genes are expressed in *salm-i/salr-i* background as a consequence of ectopic JNK activity, whereas the expression of the majority of them behaves as independent of JNK.

### Functional requirements of candidate Salm/Salr target genes

The double objective of our approach was to identify the genes that are regulated by Salm/Salr and to assign them biological functions that might account for the known Salm/Salr requirements during wing blade development. These requirements include cell division and viability, pattern formation and a variety of epithelial characteristics including cell affinity. Despite the broad requirements of Salm/Salr functions, it was unexpected to find such a large number of gene expression changes in *salm/salr* backgrounds. The in-depth analysis of each gene is beyond the scope of this work, but we identified candidates that likely play a direct role downstream of Salm/Salr in some of the processes affected by these proteins.

In the case of genes repressed by Salm/Salr, we found that most of them do not show a loss of function phenotype, as they are not normally expressed in the wing disc. We attempted to assign a requirement for these genes in the development of the *salm/salr* mutant phenotype using an RNAi approach, for which we knocked down individually all genes that are ectopically expressed in *salm/salr* discs, but we could not identify a significant individual contribution for any of the 37 genes analysed. Although we do not know to what extent inappropriate ectopic expression of genes that are negatively regulated by Salm/Salr contribute to the *spalt* loss of function phenotype in the wing, it is likely that it is their combined over-expression what contributes to the cell division and cell viability defects characteristics of *salm/salr* mutant cells. The molecular functions of these genes falls into a variety of categories, but it is remarkable that 11 of them are related to DNA metabolism, including putative transcription factors (*CG6794* and *CG6272*), DNA repair (*CG16928*, *CG1925*, *CG3448* and *CG5247*) and other putative DNA binding or modifying proteins (*CG1303*, *CG14121*, *CG45050*, *CG5096* and *CG5202*). Genes repressed by Salm/Salr also include 7 proteins that function in general metabolism (*CG17533*, *CG18522*, *CG17530*, *CG1851*, *CG33048*, *CG2999* and *CG6658*) and two gene pairs formed by a non-coding RNA and a coding gene (*CG1512*/*CG43144* and *CG12505*/*CG10102*). Intriguingly, these groups of genes are enriched in a chromatin mark (H3K9me3) related to the formation of heterochromatin, suggesting that Salm/Salr in flies may, as proposed for human Sall1 [13], participate in gene repression through its association with heterochromatin regions. Only one gene repressed by Salm/Salr, *CG18455* (*optix*), displayed a strong vein pattern phenotype that can be related to the function of Sal in defining the position of the vein L2, and our preliminary work confirms that the homeobox DNA-binding domain protein Optix is a component of the genetic network regulated by Salm/Salr in the positioning of the vein L2.

In the case of genes detected as activated by Salm/Salr, we further refined the initial list of 139 candidates (S5 Table) to a group of 34 genes which expression changed in *salm/salr* knockdown conditions and also displayed a loss-of-function phenotype (STG-OK; Table 1). The most frequent phenotypes include defects in the size of the wing (14 cases) and alterations in both the size and pattern of the wing (16 cases). These phenotypes are similar to the *salm/salr* loss-of-function conditions, suggesting that these genes might be functionally related to Sal functions. At this stage, it is tempting to speculate that genes affecting only the size of the wing may be related to the regulation of cell proliferation or cell viability, two process that are influenced by Salm/Salr in the central domain of the disc. Also, genes whose loss of function phenotypes resemble the loss of *salm/salr* function and affect simultaneously the size and pattern of the wing are of singular interest, and the corresponding genes are obvious candidates to implement the characteristics imposed by Salm/Salr to the central region of the wing blade. The group of best candidates Sal-activated genes includes three cases particularly interesting such as *CG14394* (*Ninjurin*, *NijC*), *CG1725 (disclarge*, *dlg1)*, *CG42614 (scribble*, *scrib)*. The corresponding proteins encode components related to epithelial architecture, a process that is severely compromised in *salm/salr* mutations, and consequently they are bona fide candidates to regulate this aspect of Sal function. Additional components regulating the cytoskeleton or cell adhesion were also identified in the microarrays, but they were not further considered because they did not show a loss-of-function phenotype, such as *CG7478* (*Actin 79B*) *and CG42330* (*Dscam4*), or because we did not detect a change in their expression by *in situ* hybridization (*CG43079* (*nrm*), *CG10541* (*Tektin C*) and *CG7607*).

To a large extent, development can be contemplated as the deployment of gene regulatory hierarchies and their implementation in particular cellular behaviours. The analysis of wing disc development has provided numerous examples of how signalling pathways are engaged in these processes, in part though the regulation of gene expression. However, we still had a poor understanding of the regulatory events triggered by signalling pathways or transcription factors. This work aimed to reconstruct the genetic cascade initiated by Dpp signalling that acting through the Spalt transcription factors controls the growth and patterning of the central region of the wing disc. Our analysis uncovered an unsuspected complexity in the transcriptional landscape regulated by Salm/Salr, consisting in batteries of activated and repressed genes (Fig 8E). The identification of Salm/Salr candidate target genes also opens the possibility of searching for the DNA regulatory regions that confer response to Salm/Salr, and to solve the mechanisms of Salm/Salr transcriptional regulation.

## Material and Methods

### Genetic strains

We used the following *Gal4* lines: *sal* ^*EPv*^ *-Gal4* [23], *nub-Gal4*, *sd-Gal4*, *756-Gal4* [5] and *tub*-*Gal80* ^*ts*^ [24], the *UAS* lines *UAS-GFP* [33], *UAS-salm-i* (ID 3029 VDRC), *UAS-salr-i* (ID 28386 VDRC), *UAS-puc* [34], *UAS-dicer2* [35], *UAS-sal* [5], *UAS-dad* and the *sal/salr* deficiency *Df(2L)32FP5* [36]. The expression of *sal* ^*EPv*^ *-Gal4* is restricted to the central region of the wing imaginal disc between the vein L2 and intervein L4–L5, and the expression of *nub-Gal4*, *sd-Gal4* and *756-Gal4* occurs in the entire wing pouch (*sd-Gal4*) and hinge (*756-Gal4* and *nub-Gal4*). The *UAS* lines used to express RNA interference for the genes selected in microarray experiments are listed in Table 2 and were obtained from Bloomington Stock Center, Vienna Drosophila RNAi Center (VDCR) and NIG-FLY RNAi. Unless otherwise stated, crosses were done at 25°C. Lines not described in the text can be found in FlyBase [37].

### Microarray experiments

We did two sets of microarray experiments. In one set (Experiment 1), we compared the expression profiles of 2 genetic conditions at different times:

Experiment 1:

1. *sal* ^*EPv*^ *-Gal4 UAS-GFP; tub-Gal80* ^*ts*^ */ UAS-GFP* (control discs).
2. *sal* ^*EPv*^ *-Gal4 UAS-GFP / UAS-salm-i; tub-Gal80* ^*ts*^ */ UAS-salr-i* (experimental discs).

In all experiments the larvae were raised at 25°C and then transferred to 29°C for a period of 24h (T24) or 48h (T48) before the dissection of the imaginal discs. At 25°C the Gal80^ts^ protein blocks Gal4 activity, whereas at 29°C Gal4 is active and drives the expression of the selected *UAS* constructs. We extracted mRNA from 4 biological replicas for each experimental genotype and temperature conditions: *sal* ^*EPv*^ *-Gal4 UAS-GFP/UAS-salm-i; tub-Gal80* ^*ts*^ */UAS-salr-i* 24h and 48h at 29°C and *sal* ^*EPv*^ *-Gal4 UAS-GFP; tub-Gal80* ^*ts*^ */UAS-GFP* 24h and 48h at 29°C. The total amount of mRNA varied in each replica from 2.4 to 5.1 μg (S6 Table). RNA was extracted following a TRIzol protocol (TRIzol Reagent Ambion) and posterior DNase treatment (DNA-*free* ^TM^ kit Ambion) from groups of 40–80 discs previously stored at -80°C in RNA stabilization solution (RNA*later* Ambion) until homogenisation and mRNA extraction. The RNA samples were sent to BIOARRAY (http://www.bioarray.es) for further processing and hybridization in Agilent arrays specifically designed for *Drosophila melanogaster* (ID 043135). In this work we describe exhaustively the result of these experiments.

Experiment 2:

In a second set of experiments, we compared the expression profiles of 4 genetic conditions:

1. *sd-Gal4/+; FRT40/ FRT40 M(2)z; UAS-FLP/+*
2. *sd-Gal4/+; Df(2L)sal* ^*FP5*^ *FRT40/ FRT40 M(2)z; UAS-FLP/+*
3. *756-Gal4*/ *UAS-sal*
4. *756-Gal4 / UAS-GFP*

We collected imaginal discs, extracted mRNA, and compared the expression profile of genotypes 1 with 2 (*Df*) and 3 with 4 (*UAS*). For these experiments we used Affymetrix arrays, and the genes which expression varied more consistently were selected.

Experiments 1 and 2 differs in several fundamental aspects; first, in experiment 1 all cells included in the domain of *sal* ^*EPv*^ *-Gal4* expression display a similar and simultaneous reduction in *salm* and *salr* expression, whereas in experiment 2 (Df) the wing is a mosaic of *Df(2L)sal* ^*FP5*^ homozygous and heterozygous cells. Furthermore, the over-expression of *salm* in the entire wing (UAS) causes a strong reduction in wing size [5], and many *756-Gal4*/*UAS-sal* wings develop only as stumps of wing tissue. Despite the differences between these experiments, we found that 10% of the genes identified in experiment 1 were also identified in experiment 2 (14% at 24h and 8% at 48h after temperature change). As this coincidence was low, we decided to apply more stringent criteria to restrict the number of genes selected from experiment 1 (see results). From the resulting genes, a fraction of 30% was also identified in experiment 2. To construct our final list of candidate genes to be further analysed, we finally added a set of genes displaying very good scores from experiment 2 that were not identified or selected in experiment 1 (see S3 Table). We reasoned that combining the results from both experiments, which are very different in many respects, and applying stringent selection criteria contributed to add some robustness to the final list of genes that we were able to analyse.

### Immunocytochemistry

We used rabbit anti-Salm and rat anti-Salm [36], mouse anti-FasIII (Hybridoma bank at Iowa University). Secondary antibodies were from Jackson Immunological Laboratories (used at 1/200 dilution). Imaginal wing discs were dissected, fixed and stained as described in [38]. Confocal images were captured using a LSM510 confocal microscope. All images were processed with the program ImageJ 1.45s (NIH, USA) and Adobe Photoshop CS6.

### *In situ* hybridization

Imaginal discs were dissected and fixed in 4% formaldehyde for 20 min at room temperature, washed in PBS-0.1% Tween (PBT), and re-fixed for 20 min at room temperature with 4% formaldehyde, 0.1% Tween. After three washes in PBT, discs were stored at -20°C in hybridization solution (SH; 50% formamide, SSC 5X, 100 μg/ml salmon sperm DNA, 50 μg/ml heparin and 0.1% Tween). Disc were pre-hybridized for 2 hours at 55°C in SH, and hybridized with digoxigenin-labelled RNA probes at 55°C. The probes were previously denaturalized at 80°C for 10 min. After hybridization, discs were washed in SH and PBT and incubated for 2 hours at room temperature in a 1:4000 dilution of anti-DIG antibody (Roche). After incubation, the discs were washed in PBT and the detection of probes was done with NBT and BCIP solution (Roche). The discs were mounted in 70% glycerol. Pictures were taken using a Spot digital camera coupled to a Zeiss Axioplam microscope using the 20X objectives. All images were processed with Adobe Photoshop CS6.

The probes were generated using cDNA from the collections of *Expression Sequence Tags* (*EST*) of Berkeley Drosophila Genome Project, or by PCR from genomic DNA (S3 and S7 Tables), using primers with RNA polymerase T7 and T3 sequences in their ends described in S7 Table. The transcription was done using the RNA polymerase T7, T3 or SP6 (Roche) during 2 hours and the probes were precipitated and suspended in H_2_O DEPC.

### Functional annotation: Gene Ontology (GO) analysis

Functional annotation of Gene Ontology terms was performed using the software DAVID [28–29]. We searched for the GO term “Biological process” (GOTERM_BP_4) in a list of FlyBase Gene ID for the collections of genes identified in the microarray experiments against the *Drosophila melanogaster* genome as background. We accepted as a significant enrichment those GO terms who have a p-value lower than 0.05.

## Supporting Information

S1 Fig (A-A’’’) Imaginal disc of *sal* ^*EPv*^ *-Gal4 UAS-GFP /UAS-salm-i; tub-Gal80* ^*ts*^ */UAS-salr-i* genotype (*salm-i/salr-i* 24h) raised at 29°C 24–28 hours before dissection, showing the expression of GFP (green), Salm (red) and FasIII (blue). Independent channels showing GFP, Salm and FasIII are shown in A’, A’’ and C’’’ respectively. (B-B’’’) Imaginal disc of *sal* ^*EPv*^ *-Gal4 UAS-GFP /UAS-salm-i; tub-Gal80* ^*ts*^ */UAS-salr-i* genotype (*salm-i/salr-i* 48h) raised at 29°C 44–48 hours before dissection showing the expression of GFP (green), Salm (red) and FasIII (blue). Independent channels showing GFP, Salm and FasIII are shown in B’, B’’ and B’’’ respectively.
(TIF)

S2 Fig Functional annotation of candidate STG genes. (A-B) Distribution of functional categories of candidate STG genes which expression levels decreases (A; STG-, n = 139; C) or increases (B; STG+, n = 147; D). D (genes related with the biology of the DNA), P (genes related with the biology of the proteins metabolism), CG (genes without known functional domains or orthology), CS (genes encoding components of signaling pathways), CGh (genes with a functional domain but not clear orthology relationships), M (genes encoding proteins related to the metabolism of lipids or glucids), CA+Cyt (genes related with cell adhesion or the cytoskeleton), Cut (genes encoding proteins related with structural constituent of cuticle), R (genes related with the biology of the RNA), Tra (genes related with the transport of metabolites across cellular membranes), RedOx (genes encoding proteins related with oxidation-reduction process), CD (genes related with cell death), Neu (genes encoding neurotransmitters), CR (non protein coding gene) and CDiv (genes related with cell division).
(TIF)

S3 Fig *In situ* hybridization in late third instar wing discs of genes which expression levels increase by microarray in the 24h and 48 h classes of experiment 1 and for which we could detect a change in their expression patterns. The name of each gene is indicated in the bottom of each left panel (A-Y), and the expression patterns class and logFC to the top and to the bottom, respectively, of each right panel (A’-Y’). In each pair of panels, A-Y corresponds to wild type discs and A’-Y’ to *UAS-dicer2/+; sal* ^*EPv*^ *-Gal4 UAS-GFP/UAS-salm-i; UAS-salr-i/+* wing discs.
(TIF)

S4 Fig *In situ* hybridization in late third instar wing discs of genes which expression levels change comparing *756-Gal4/UAS-sal* vs *756-Gal4/UAS-GFP* (UAS) or wild type vs *Df(2L)32FP5* (Df) discs (experiment 2), and for which we could detect a change in their expression patterns. The name of each gene is indicated in the bottom of each left panel (A-O), and the expression patterns class and experiment in which the gene was identified (UAS and/or Df) to the top and to the bottom, respectively, of each right panel (A’-O’). In each pair of panels, A-O corresponds to wild type discs and A’-O’ to *UAS-dicer2/+; sal* ^*EPv*^ *-Gal4 UAS-GFP/UAS-salm-i; UAS-salr-i/+* wing discs.
(TIF)

S5 Fig *In situ* hybridization in late third instar wing discs of genes which expression levels increase by microarray in the 24h and 48 h classes of experiment 1 and for which we were unable to detect a change in their expression patterns by *in situ* hybridization. The name of each gene is indicated in the bottom of each left panel (A-R), and the expression patterns class and logFC to the top and to the bottom, respectively, of each right panel (A’-R’). In each pair of panels, A-R corresponds to wild type discs and A’-R’ to *UAS-dicer2/+; sal* ^*EPv*^ *-Gal4 UAS-GFP/UAS-salm-i; UAS-salr-i/+* wing discs.
(TIF)

S6 Fig *In situ* hybridization in late third instar wing discs of genes which expression levels increase by microarray in the 24h and 48 h classes of experiment 1 and for which we were unable to detect a change in their expression patterns by *in situ* hybridization. The name of each gene is indicated in the bottom of each left panel (A-R), and the expression patterns class and logFC to the top and to the bottom, respectively, of each right panel (A’-R’). In each pair of panels, A-R corresponds to wild type discs and A’-R’ to *UAS-dicer2/+; sal* ^*EPv*^ *-Gal4 UAS-GFP/UAS-salm-i; UAS-salr-i/+* wing discs.
(TIF)

S7 Fig *In situ* hybridization in late third instar wing discs of genes which expression levels increase by microarray in the 24h and 48 h classes of experiment 1 and for which we were unable to detect a change in their expression patterns by *in situ* hybridization. The name of each gene is indicated in the bottom of each left panel (A-X), and the expression patterns class and logFC to the top and to the bottom, respectively, of each right panel (A’-X’). In each pair of panels, A-X corresponds to wild type discs and A’-X’ to *UAS-dicer2/+; sal* ^*EPv*^ *-Gal4 UAS-GFP/UAS-salm-i; UAS-salr-i/+* wing discs.
(TIF)

S8 Fig *In situ* hybridization in late third instar wing discs of genes ectopically expressed in the central domain of *salm-i/salr-i* wing discs. The name of each gene is indicated to the in the bottom of each left panel (A-N-1). The expression patterns is indicated to the top of each right panel (A’ to N-1’). Left panel in each pair correspond to wing discs of *sal* ^*EPv*^ *-Gal4 UAS-GFP/ UAS-salm-i; UAS-salr-i/UAS-GFP* genotype, and right panel in each pair correspond to wing discs of *sal* ^*EPv*^ *-Gal4/UAS-salm-i; UAS-salr-i/UAS-puc* genotype The images A-H’ correspond to genes which ectopic expression is cancelled by *puc* over-expression. The images I-N-1’ correspond to genes which ectopic expression is not cancelled by *puc* over-expression.
(TIF)

S9 Fig *In situ* hybridization in late third instar wing discs of genes which expression levels is reduced in the 24h and 48h classes of experiment 1 and for which we could detect a change in their expression patterns. The name of each gene is indicated in the bottom of each left panel (A to D-1), and the expression patterns class and logFC to the top and to the bottom, respectively, of each right panel (A’ to D-1’). In each pair of panels, A-D-1 corresponds to wild type discs and A’-D-1’ to *UAS-dicer2/+; sal* ^*EPv*^ *-Gal4 UAS-GFP/UAS-salm-i; UAS-salr-i/+* wing discs.
(TIF)

S10 Fig *In situ* hybridization in late third instar wing discs of genes which expression levels change comparing *756-Gal4/UAS-sal* vs *756-Gal4/UAS-GFP* (UAS) or wild type vs *Df(2L)32FP5* (Df) discs (experiment 2), and for which we could detect loss of expression in the central domain of the wing disc. These genes are all expressed in a restricted manner. The name of each gene is indicated in the bottom of each left panel (A-K), and the expression patterns class and experiment in which the gene was identified (UAS and/or Df) to the top and to the bottom, respectively, of each right panel (A’-K’). In each pair of panels, A-K corresponds to wild type discs and A’-K to *UAS-dicer2/+; sal* ^*EPv*^ *-Gal4 UAS-GFP/UAS-salm-i; UAS-salr-i/+* wing discs.
(TIF)

S11 Fig *In situ* hybridization in late third instar wing discs of genes which expression levels change comparing *756-Gal4/UAS-sal* vs *756-Gal4/UAS-GFP* (UAS) or wild type vs *Df(2L)32FP5* (Df) discs (experiment 2), and for which we could detect loss of expression in the central domain of the wing disc. These genes are all expressed in a generalised manner. The name of each gene is indicated in the bottom of each left panel (A to J-1), and the expression patterns class and experiment in which the gene was identified (UAS and/or Df) to the top and to the bottom, respectively, of each right panel (A’ to J-1’). In each pair of panels, A-J-1 corresponds to wild type discs and A’-J-1’ to *UAS-dicer2/+; sal* ^*EPv*^ *-Gal4 UAS-GFP/UAS-salm-i; UAS-salr-i/+* wing discs.
(TIF)

S12 Fig *In situ* hybridization in late third instar wing discs of genes which expression levels change comparing *756-Gal4/UAS-sal* vs *756-Gal4/UAS-GFP* (UAS) or wild type vs *Df(2L)32FP5* (Df) discs (experiment 2), and for which we could detect loss of expression in the central domain of the wing disc. These genes are all expressed in a generalised manner. The name of each gene is indicated in the bottom of each left panel (A to E-1), and the expression patterns class and experiment in which the gene was identified (UAS and/or Df) to the top and to the bottom, respectively, of each right panel (A’ to E-1’). In each pair of panels, A-E-1 corresponds to wild type discs and A’-E-1’ to *UAS-dicer2/+; sal* ^*EPv*^ *-Gal4 UAS-GFP/UAS-salm-i; UAS-salr-i/+* wing discs.
(TIF)

S13 Fig *In situ* hybridization in late third instar wing discs of genes which expression levels is reduced in the 24h and/or 48h classes of experiment 1 and for which we could not detect a change in their expression patterns. The name of each gene is indicated in the bottom of each left panel (A to B-1), and the expression patterns class and logFC to the top and to the bottom, respectively, of each right panel (A’ to B-1’). (C-1-D-1’) *In situ* hybridization in late third instar wing discs of genes which expression levels change comparing *756-Gal4/UAS-sal* vs *756-Gal4/UAS-GFP* (UAS) or wild type vs *Df(2L)32FP5* discs (experiment 2), and for which we could not detect loss of expression in the central domain of the wing disc. These genes are all expressed in a restricted manner. The name of each gene is indicated in the bottom of each left panel (C-1 and D-1), and the expression patterns class and experiment in which the gene was identified (UAS and/or Df) to the top and to the bottom, respectively, of each right panel (C-1’ and D-1’). In each pair of panels, A-D-1 corresponds to wild type discs and A’-D-1’ to *UAS-dicer2/+; sal* ^*EPv*^ *-Gal4 UAS-GFP/UAS-salm-i; UAS-salr-i/+* wing discs.
(TIF)

S14 Fig Representative examples of adult wings of combinations involving either *nub-Gal4* or *sal* ^*EPv*^ *-Gal4* and *UAS-RNA-i* lines targeting the genes which expression is increased in *UAS-dicer2/+; sal* ^*EPv*^ *-Gal4/UAS-salm-i; UAS-salr-i/+* wing discs. The genotype and the phenotype of each combination are indicated at the bottom and at the top of panels A-V.
(TIF)

S15 Fig Representative examples of adult wings of combinations involving either *nub-Gal4* or *sal* ^*EPv*^ *-Gal4* and *UAS RNA-i* lines targeting the genes which expression is reduced in *UASdicer2/+; sal* ^*EPv*^ *-Gal4/UAS-salm-i; UAS-salr-i/+* wing discs. The genotype and the phenotype of each combination are indicated at the bottom and at the top of panels A-R-1.
(TIF)

S1 Table Genes identified in the comparison of *sal* ^*EPv*^ *-Gal4 UAS-GFP /UAS-salm-i; tub-Gal80* ^*ts*^ */UAS-salr-i* wing discs at 24 and 48 hours after temperature shift. (XLSX)

S2 Table Genes identified in the 24 and 48 hours comparison between of *sal* ^*EPv*^ *-Gal4 UAS-GFP /UAS-salm-i; tub-Gal80* ^*ts*^ */UAS-salr-i* and *sal* ^*EPv*^ *-Gal4 UAS-GFP/+; tub-Gal80* ^*ts*^ */ UAS-GFP* control discs. (XLSX)

S3 Table Selected genes with expression levels increased (Spalt +) and decreased (Spalt-) of experiments 1 and 2 (Df//UAS). Microarray data of experiment 1 indicating logFoldChange (“logFC”) and adjusted p-value (“adj.P.Val”) at 24h and 48h at 29°C. “cDNA/PCR” indicates the generation of RNA probes using cDNA or genomic DNA (PCR). “In situ” indicates the mRNA expression patterns in wild type and *salm-i/salr-i* discs (N: expression not detected; U: generalised expression and P: patterned expression). We indicated by “=“, “+” and “-”that the expression does not change, appears ectopic or is reduced in *salmi*/*salri* discs, respectively. “Human” indicates the human orthologous.
(PDF)

S4 Table Enrichment in GO categories. Highlighted in grey scale data with a significant Bonferoni correction.
(XLSX)

S5 Table Selected genes with expression levels increased or decreased of experiments 1 (*Spalt +* and *Spalt-*, respectively) and 2 (Df//UAS). “RNAi strains” indicates the RNAi strains used in the phenotypic analysis. “Phenotype” indicates the phenotype of *nub-Gal4/UAS-RNA-i* and *sal* ^*EPv*^ *-Gal4/UAS-RNA-i* separated by double dash: + (WT); S (wing size); P (vein pattern); S-P (wing size and vein pattern); V+ and V- (extra-veins and loss of veins); nW (no wing); F (folded); Bs (dorso-ventral wing surface adhesion); N (loss of wing margin structures); L, EPL and PL (lethal, early pupal lethal and pupal lethal); CD (cell death) and Cell. Pig. (Cellular pigmentation). The phenotypes with a small letter “s” or “w” indicate whether the phenotype is strong (s) or weak (w). “Molecular” indicates the molecular nature and simplified molecular class, respectively: D (genes related with the biology of DNA), P (genes related with proteins metabolism), CG (genes without known functional domains or orthology), CS (genes encoding components of signalling pathways), CGh (genes with a known functional domain but not clear orthology relationships), M (genes encoding proteins related to the metabolism of lipids or glucids), CA+Cyt (genes related with cell adhesion or the cytoskeleton), Cut (genes encoding proteins related with structural constituent of cuticle), R (genes related with the biology of the RNA), Tra (genes related with the transport of metabolites across cellular membranes), RedOx (genes encoding proteins related with oxidation-reduction process), CD (genes related with cell death), Neu (genes encoding neurotransmitters), CR (non protein coding gene) and CDiv (genes related with cell division).
(PDF)

S6 Table RNA concentration in the different biological replicates of Experiment 1. (PDF)

S7 Table Oligonucleotides used to generate probes for *in situ* hybridization experiments using the RNA polymerase T3 and T7. Each oligonucleotide contains the sequence of the corresponding RNA polymerase (T3: ATTAACCCTCACTAAAGGGA; T7: TAATACGACTCACTATAGGG).
(PDF)
