# Supplementary figures and images for: The Spalt Transcription Factors Generate the Transcriptional Landscape of the Drosophila melanogaster Wing Pouch Central Region
Source: PLoS Genet. 2015 Aug 4;11(8):e1005370. doi: 10.1371/journal.pgen.1005370 (PMC4524721; doi:10.1371/journal.pgen.1005370)

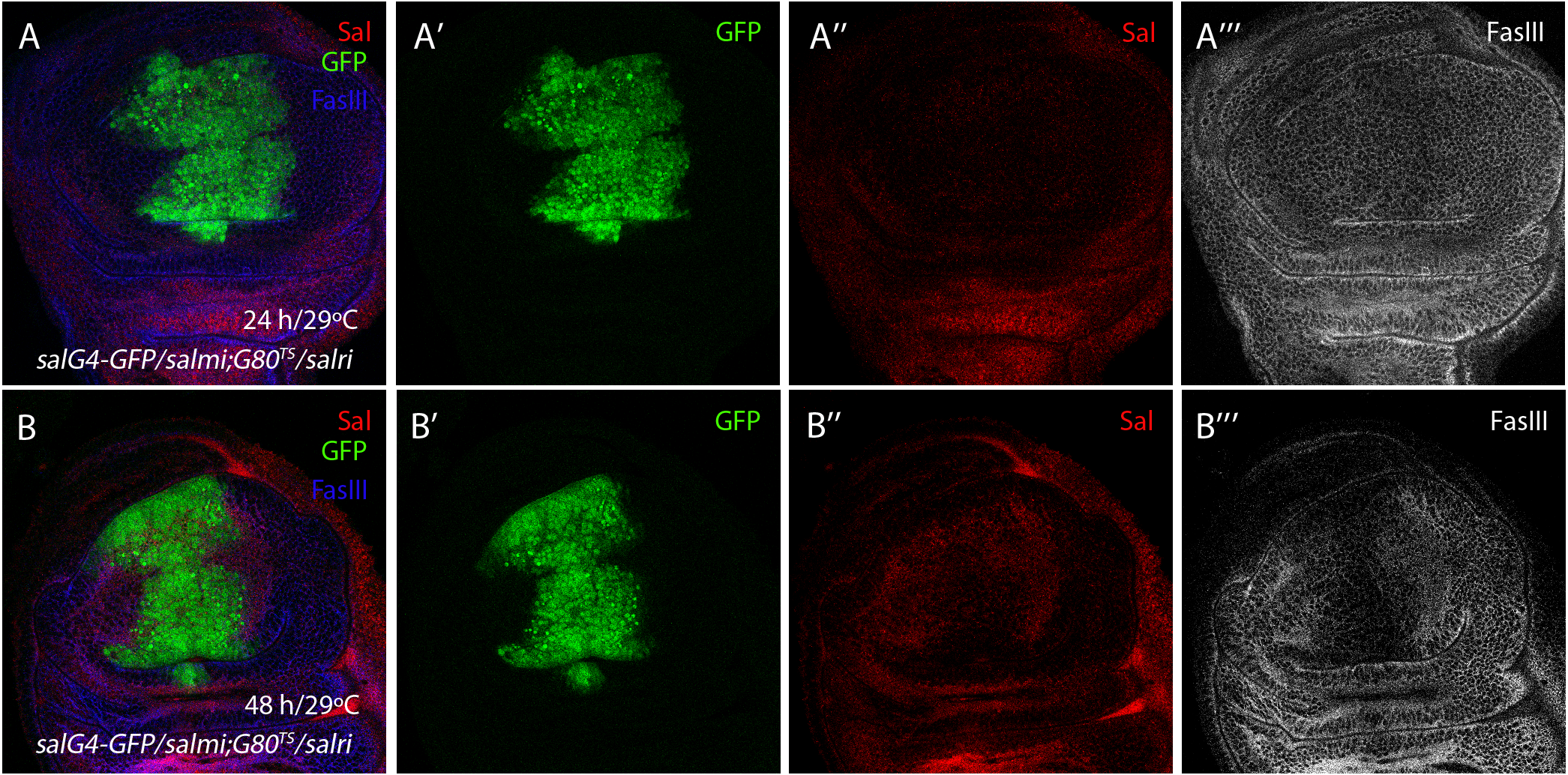

Supplement: S1 Fig — (A-A’’’) Imaginal disc of sal EPv -Gal4 UAS-GFP /UAS-salm-i; tub-Gal80 ts /UAS-salr-i genotype (salm-i/salr-i 24h) raised at 29°C 24–28 hours before dissection, showing the expression of GFP (green), Salm (red) and FasIII (blue). Independent channels showing GFP, Salm and FasIII are shown in A’, A’’ and C’’’ respectively. (B-B’’’) Imaginal disc of sal EPv -Gal4 UAS-GFP /UAS-salm-i; tub-Gal80 ts /UAS-salr-i genotype (salm-i/salr-i 48h) raised at 29°C 44–48 hours before dissection showing the expression of GFP (green), Salm (red) and FasIII (blue). Independent channels showing GFP, Salm and FasIII are shown in B’, B’’ and B’’’ respectively. (TIF) [file pgen.1005370.s001.tif]

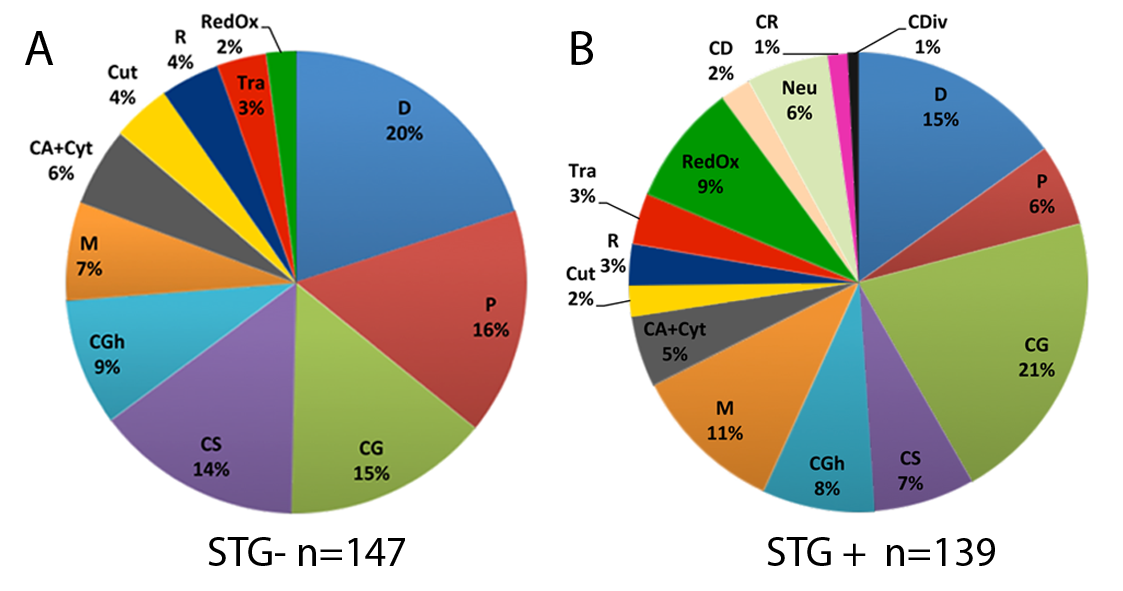

Supplement: S2 Fig — (A-B) Distribution of functional categories of candidate STG genes which expression levels decreases (A; STG-, n = 139; C) or increases (B; STG+, n = 147; D). D (genes related with the biology of the DNA), P (genes related with the biology of the proteins metabolism), CG (genes without known functional domains or orthology), CS (genes encoding components of signaling pathways), CGh (genes with a functional domain but not clear orthology relationships), M (genes encoding proteins related to the metabolism of lipids or glucids), CA+Cyt (genes related with cell adhesion or the cytoskeleton), Cut (genes encoding proteins related with structural constituent of cuticle), R (genes related with the biology of the RNA), Tra (genes related with the transport of metabolites across cellular membranes), RedOx (genes encoding proteins related with oxidation-reduction process), CD (genes related with cell death), Neu (genes encoding neurotransmitters), CR (non protein coding gene) and CDiv (genes related with cell division). (TIF) [file pgen.1005370.s002.tif]

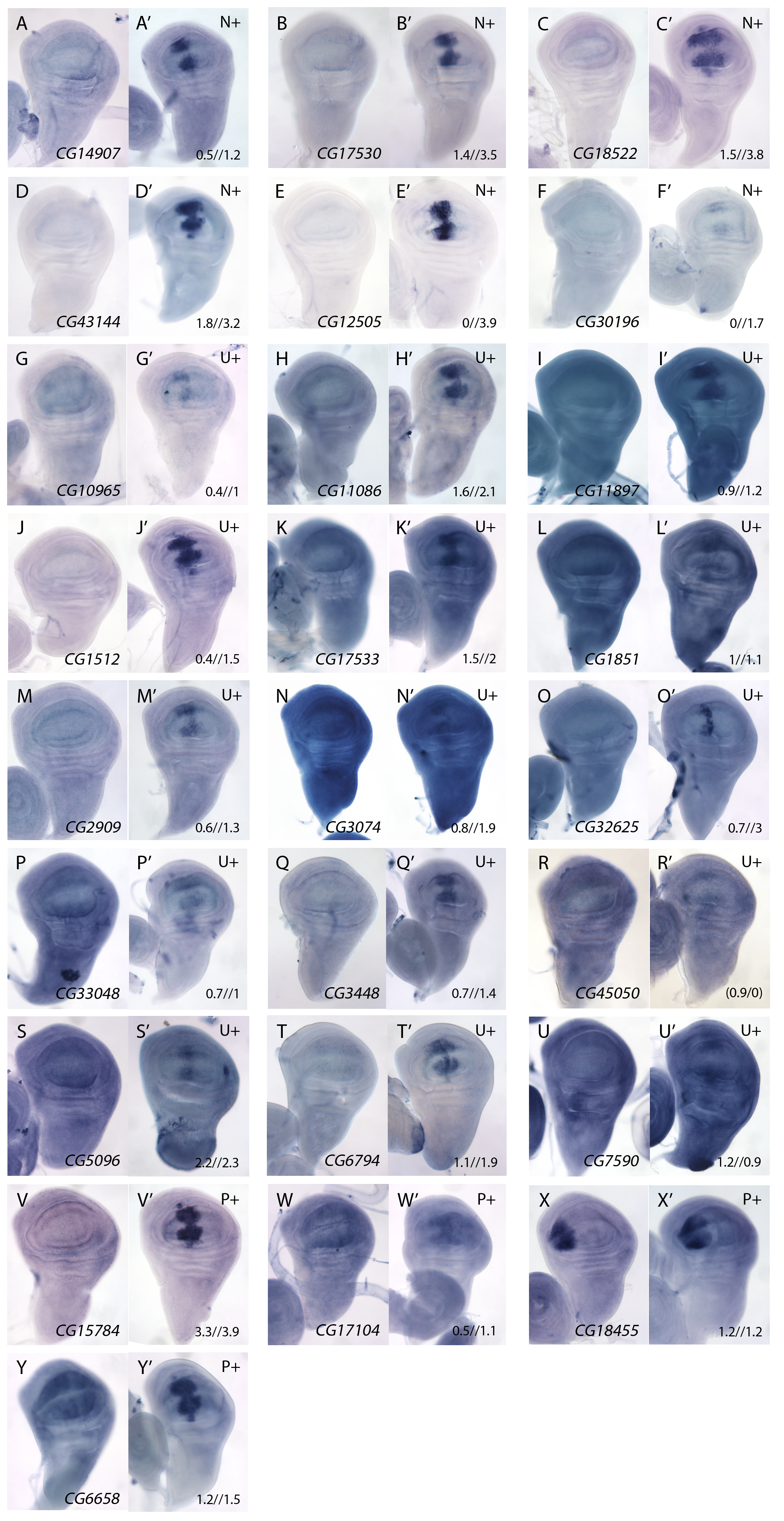

Supplement: S3 Fig — The name of each gene is indicated in the bottom of each left panel (A-Y), and the expression patterns class and logFC to the top and to the bottom, respectively, of each right panel (A’-Y’). In each pair of panels, A-Y corresponds to wild type discs and A’-Y’ to UAS-dicer2/+; sal EPv -Gal4 UAS-GFP/UAS-salm-i; UAS-salr-i/+ wing discs. (TIF) [file pgen.1005370.s003.tif]

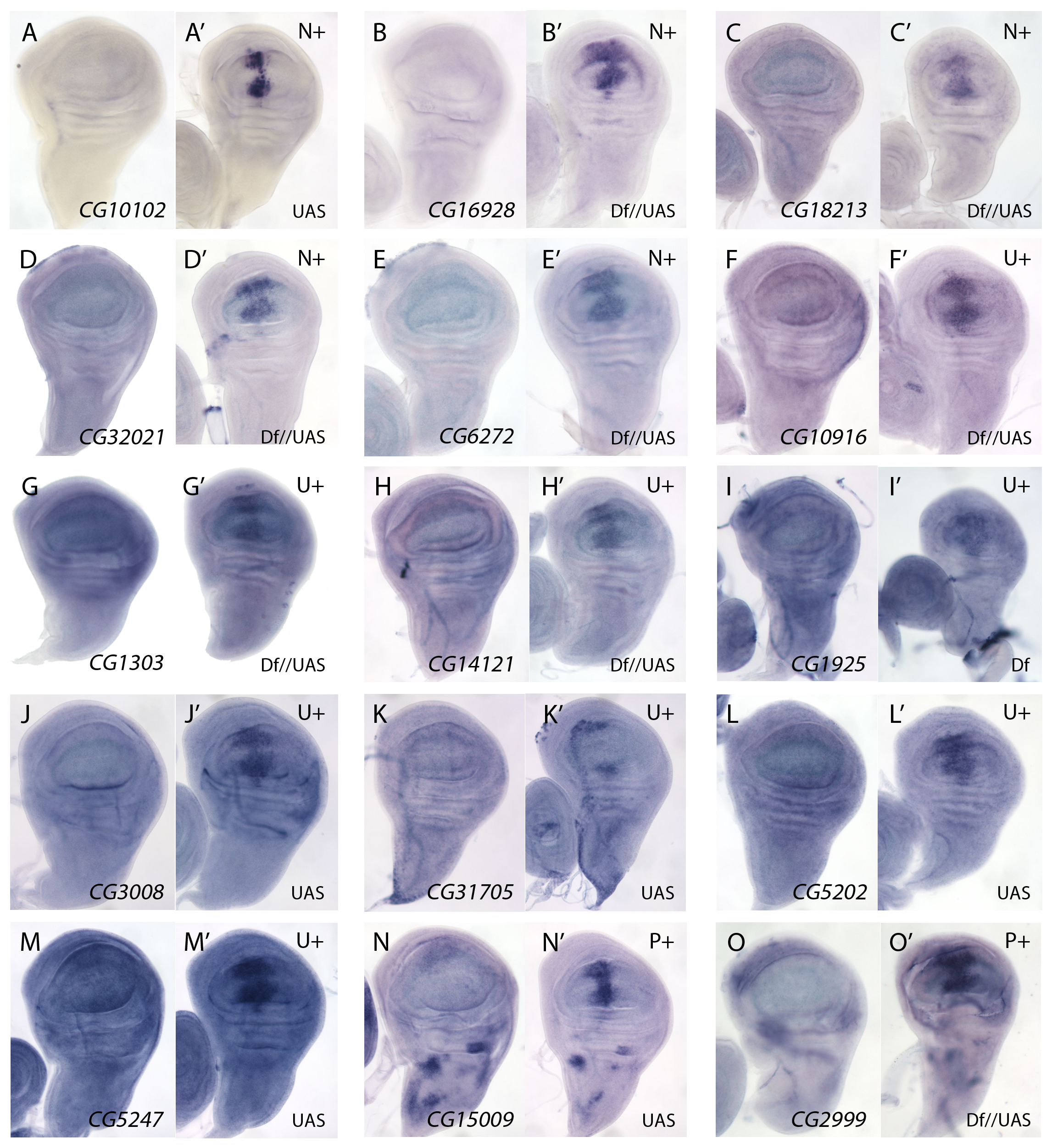

Supplement: S4 Fig — The name of each gene is indicated in the bottom of each left panel (A-O), and the expression patterns class and experiment in which the gene was identified (UAS and/or Df) to the top and to the bottom, respectively, of each right panel (A’-O’). In each pair of panels, A-O corresponds to wild type discs and A’-O’ to UAS-dicer2/+; sal EPv -Gal4 UAS-GFP/UAS-salm-i; UAS-salr-i/+ wing discs. (TIF) [file pgen.1005370.s004.tif]

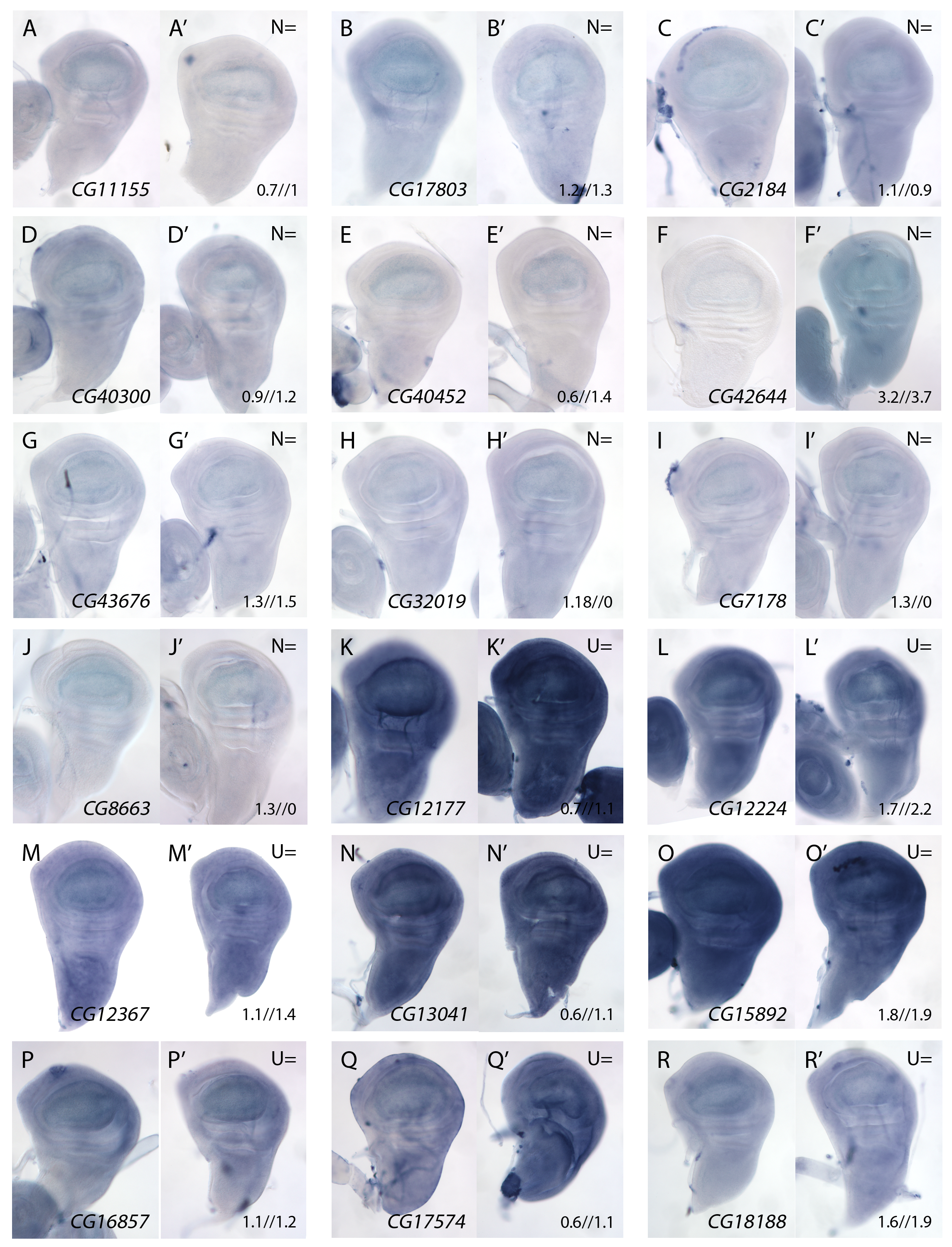

Supplement: S5 Fig — The name of each gene is indicated in the bottom of each left panel (A-R), and the expression patterns class and logFC to the top and to the bottom, respectively, of each right panel (A’-R’). In each pair of panels, A-R corresponds to wild type discs and A’-R’ to UAS-dicer2/+; sal EPv -Gal4 UAS-GFP/UAS-salm-i; UAS-salr-i/+ wing discs. (TIF) [file pgen.1005370.s005.tif]

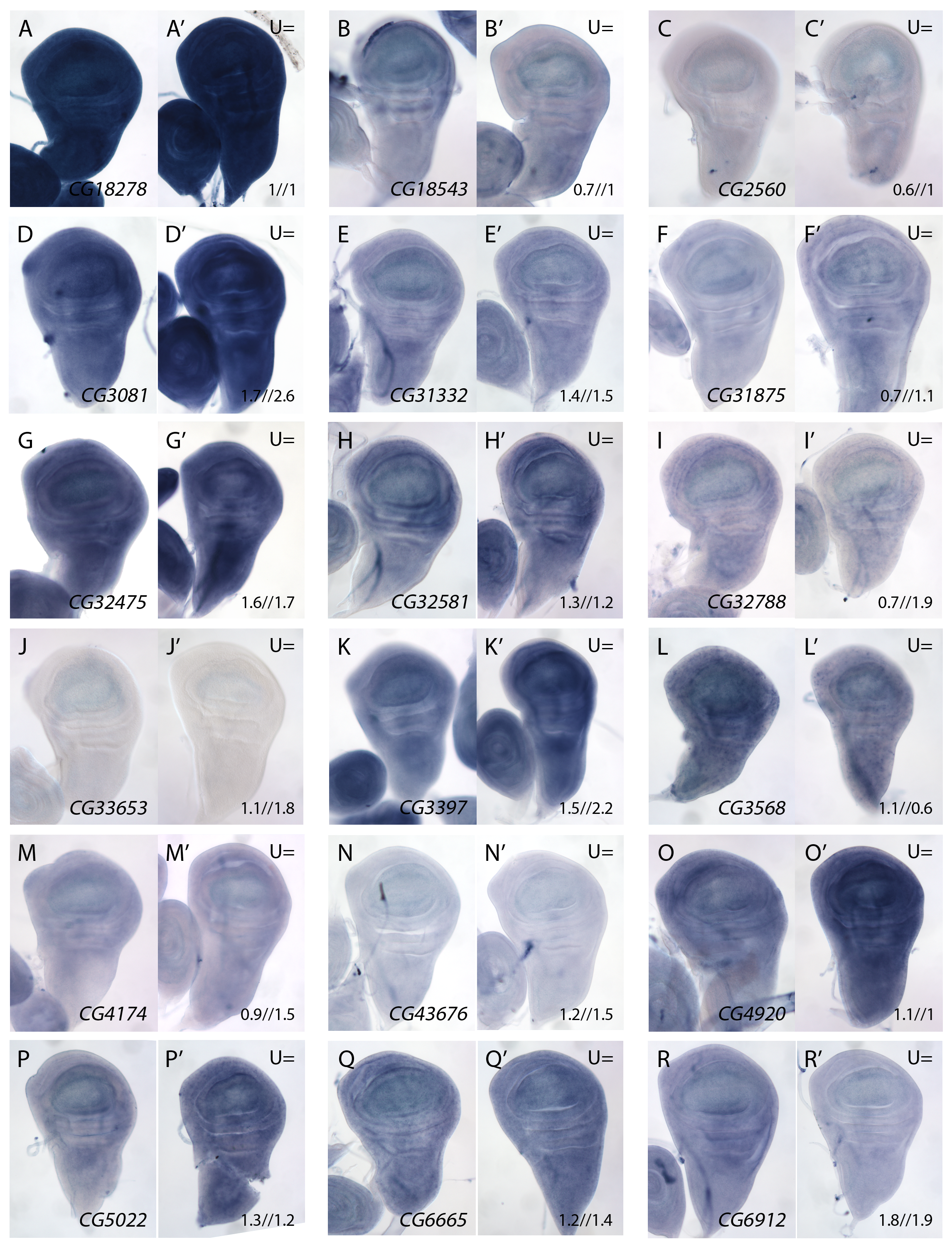

Supplement: S6 Fig — The name of each gene is indicated in the bottom of each left panel (A-R), and the expression patterns class and logFC to the top and to the bottom, respectively, of each right panel (A’-R’). In each pair of panels, A-R corresponds to wild type discs and A’-R’ to UAS-dicer2/+; sal EPv -Gal4 UAS-GFP/UAS-salm-i; UAS-salr-i/+ wing discs. (TIF) [file pgen.1005370.s006.tif]

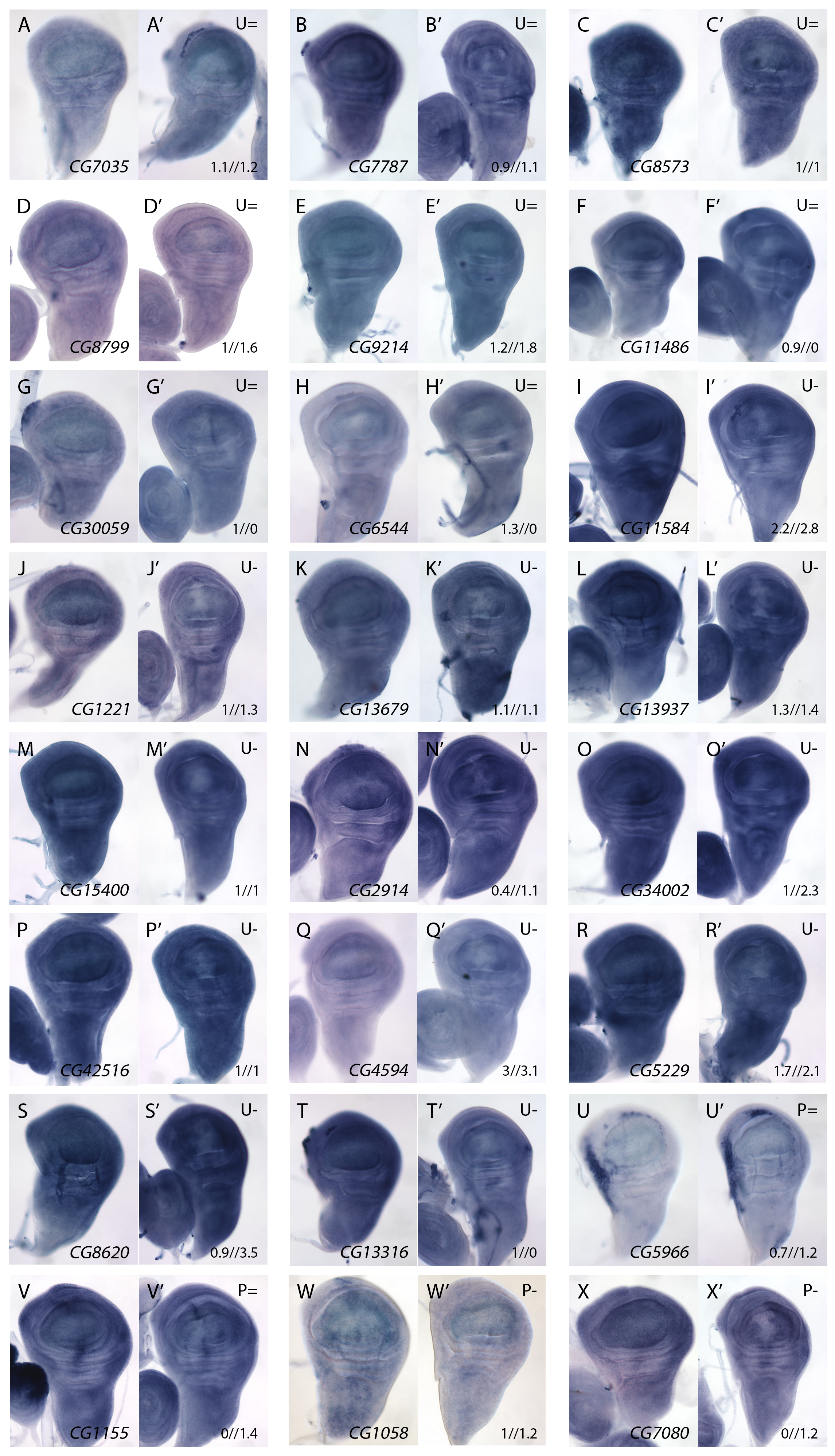

Supplement: S7 Fig — The name of each gene is indicated in the bottom of each left panel (A-X), and the expression patterns class and logFC to the top and to the bottom, respectively, of each right panel (A’-X’). In each pair of panels, A-X corresponds to wild type discs and A’-X’ to UAS-dicer2/+; sal EPv -Gal4 UAS-GFP/UAS-salm-i; UAS-salr-i/+ wing discs. (TIF) [file pgen.1005370.s007.tif]

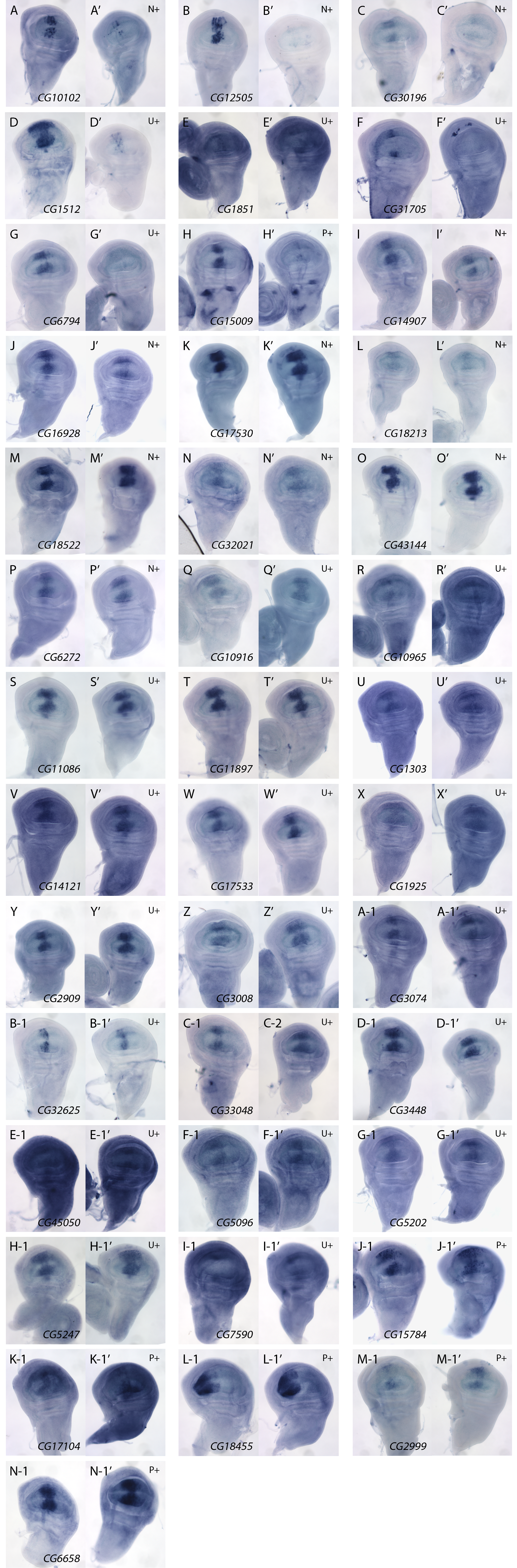

Supplement: S8 Fig — The name of each gene is indicated to the in the bottom of each left panel (A-N-1). The expression patterns is indicated to the top of each right panel (A’ to N-1’). Left panel in each pair correspond to wing discs of sal EPv -Gal4 UAS-GFP/ UAS-salm-i; UAS-salr-i/UAS-GFP genotype, and right panel in each pair correspond to wing discs of sal EPv -Gal4/UAS-salm-i; UAS-salr-i/UAS-puc genotype The images A-H’ correspond to genes which ectopic expression is cancelled by puc over-expression. The images I-N-1’ correspond to genes which ectopic expression is not cancelled by puc over-expression. (TIF) [file pgen.1005370.s008.tif]

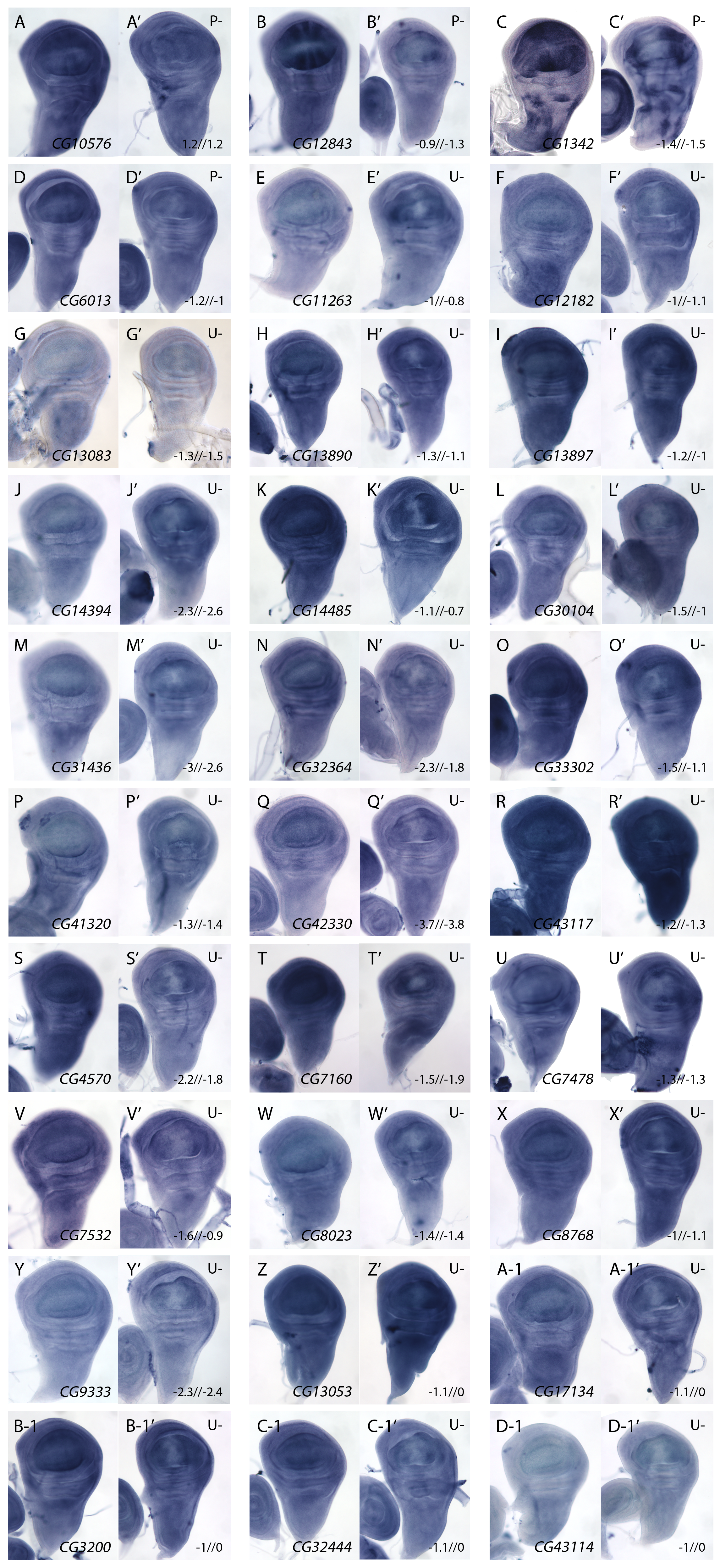

Supplement: S9 Fig — The name of each gene is indicated in the bottom of each left panel (A to D-1), and the expression patterns class and logFC to the top and to the bottom, respectively, of each right panel (A’ to D-1’). In each pair of panels, A-D-1 corresponds to wild type discs and A’-D-1’ to UAS-dicer2/+; sal EPv -Gal4 UAS-GFP/UAS-salm-i; UAS-salr-i/+ wing discs. (TIF) [file pgen.1005370.s009.tif]

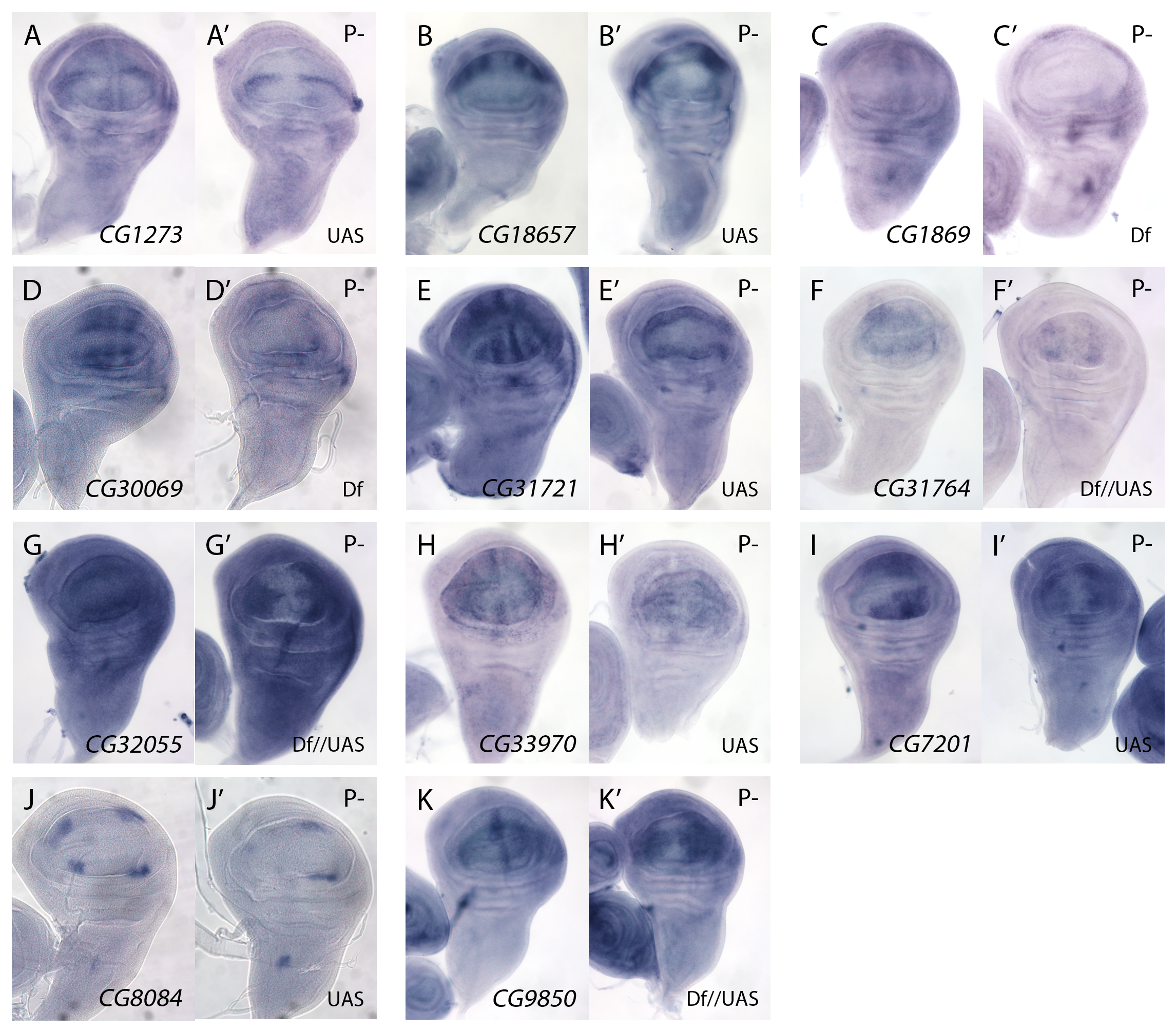

Supplement: S10 Fig — These genes are all expressed in a restricted manner. The name of each gene is indicated in the bottom of each left panel (A-K), and the expression patterns class and experiment in which the gene was identified (UAS and/or Df) to the top and to the bottom, respectively, of each right panel (A’-K’). In each pair of panels, A-K corresponds to wild type discs and A’-K to UAS-dicer2/+; sal EPv -Gal4 UAS-GFP/UAS-salm-i; UAS-salr-i/+ wing discs. (TIF) [file pgen.1005370.s010.tif]

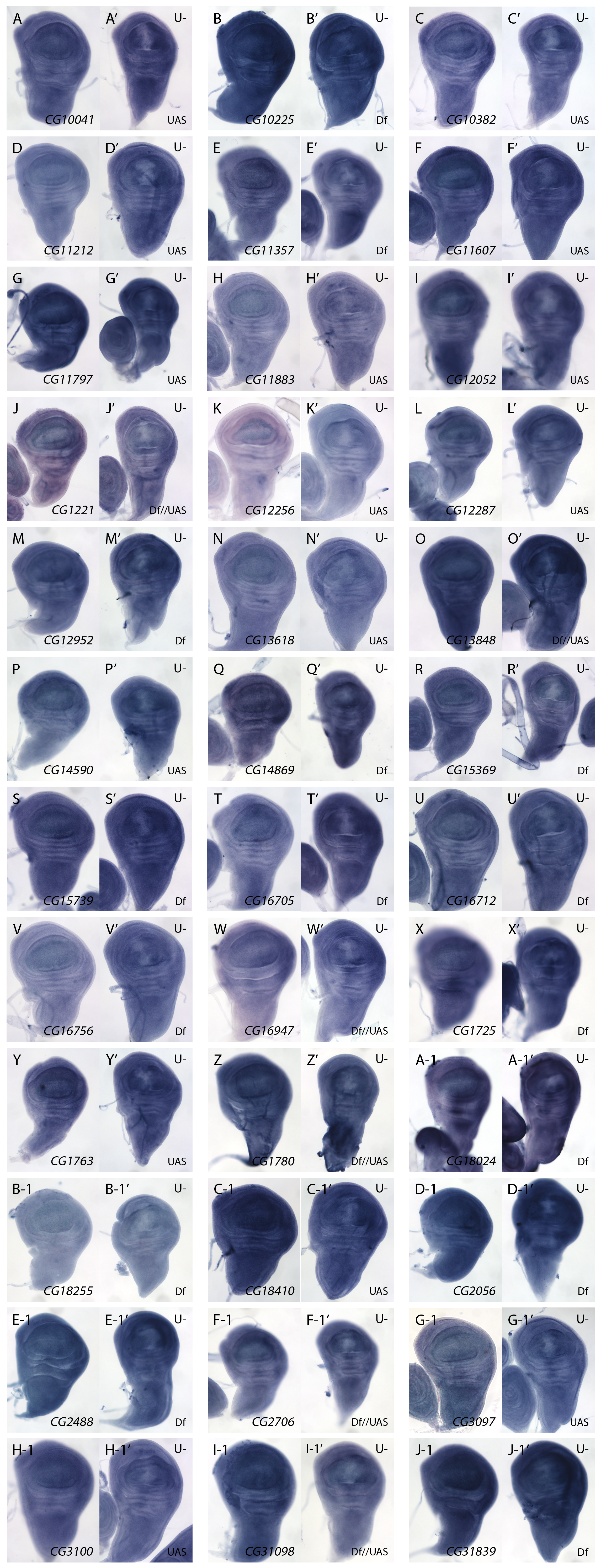

Supplement: S11 Fig — These genes are all expressed in a generalised manner. The name of each gene is indicated in the bottom of each left panel (A to J-1), and the expression patterns class and experiment in which the gene was identified (UAS and/or Df) to the top and to the bottom, respectively, of each right panel (A’ to J-1’). In each pair of panels, A-J-1 corresponds to wild type discs and A’-J-1’ to UAS-dicer2/+; sal EPv -Gal4 UAS-GFP/UAS-salm-i; UAS-salr-i/+ wing discs. (TIF) [file pgen.1005370.s011.tif]

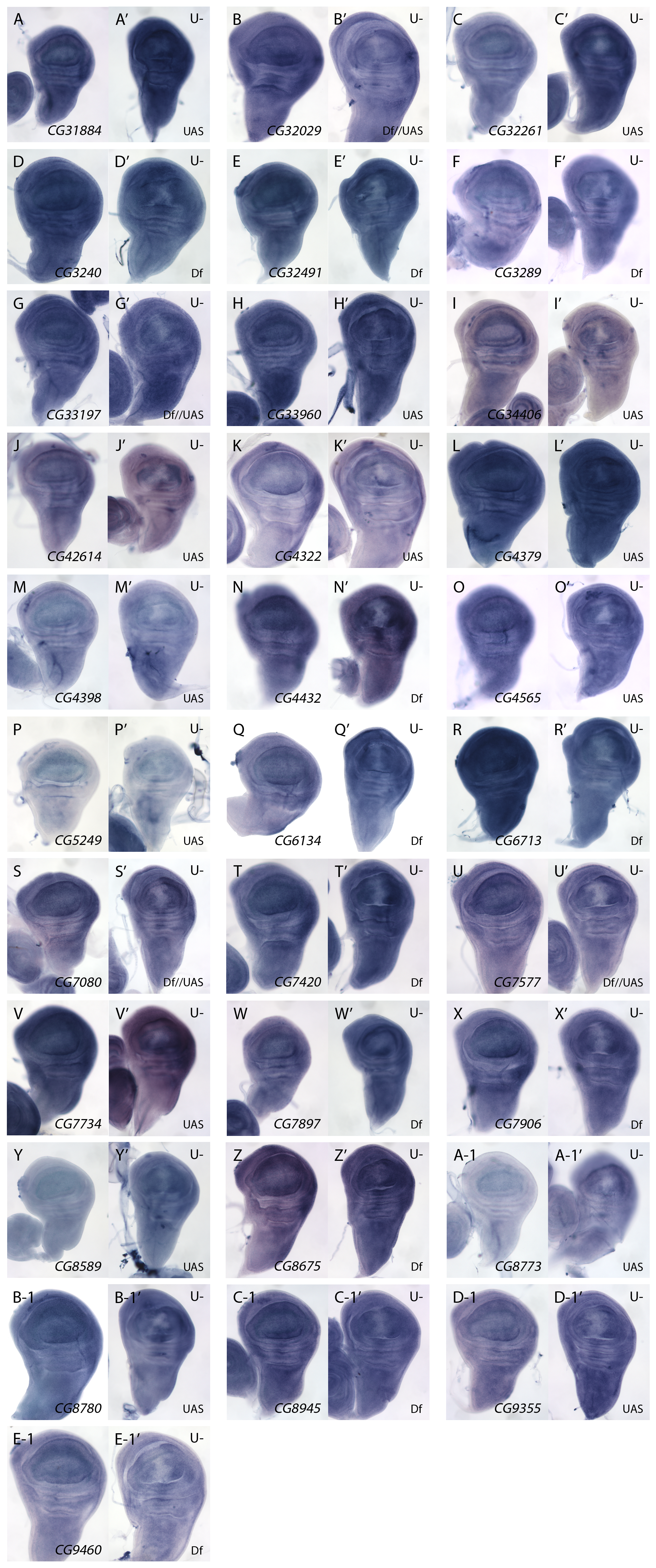

Supplement: S12 Fig — These genes are all expressed in a generalised manner. The name of each gene is indicated in the bottom of each left panel (A to E-1), and the expression patterns class and experiment in which the gene was identified (UAS and/or Df) to the top and to the bottom, respectively, of each right panel (A’ to E-1’). In each pair of panels, A-E-1 corresponds to wild type discs and A’-E-1’ to UAS-dicer2/+; sal EPv -Gal4 UAS-GFP/UAS-salm-i; UAS-salr-i/+ wing discs. (TIF) [file pgen.1005370.s012.tif]

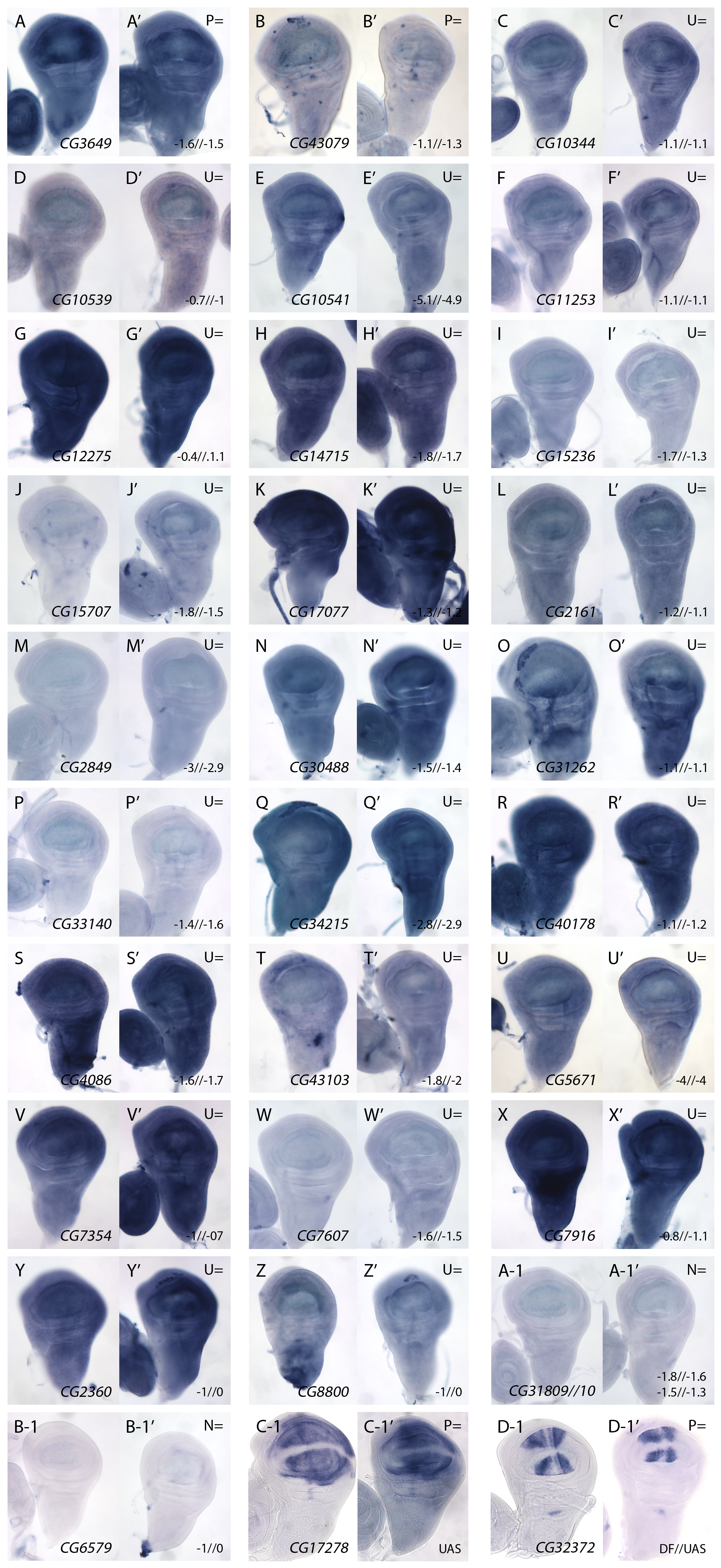

Supplement: S13 Fig — The name of each gene is indicated in the bottom of each left panel (A to B-1), and the expression patterns class and logFC to the top and to the bottom, respectively, of each right panel (A’ to B-1’). (C-1-D-1’) In situ hybridization in late third instar wing discs of genes which expression levels change comparing 756-Gal4/UAS-sal vs 756-Gal4/UAS-GFP (UAS) or wild type vs Df(2L)32FP5 discs (experiment 2), and for which we could not detect loss of expression in the central domain of the wing disc. These genes are all expressed in a restricted manner. The name of each gene is indicated in the bottom of each left panel (C-1 and D-1), and the expression patterns class and experiment in which the gene was identified (UAS and/or Df) to the top and to the bottom, respectively, of each right panel (C-1’ and D-1’). In each pair of panels, A-D-1 corresponds to wild type discs and A’-D-1’ to UAS-dicer2/+; sal EPv -Gal4 UAS-GFP/UAS-salm-i; UAS-salr-i/+ wing discs. (TIF) [file pgen.1005370.s013.tif]

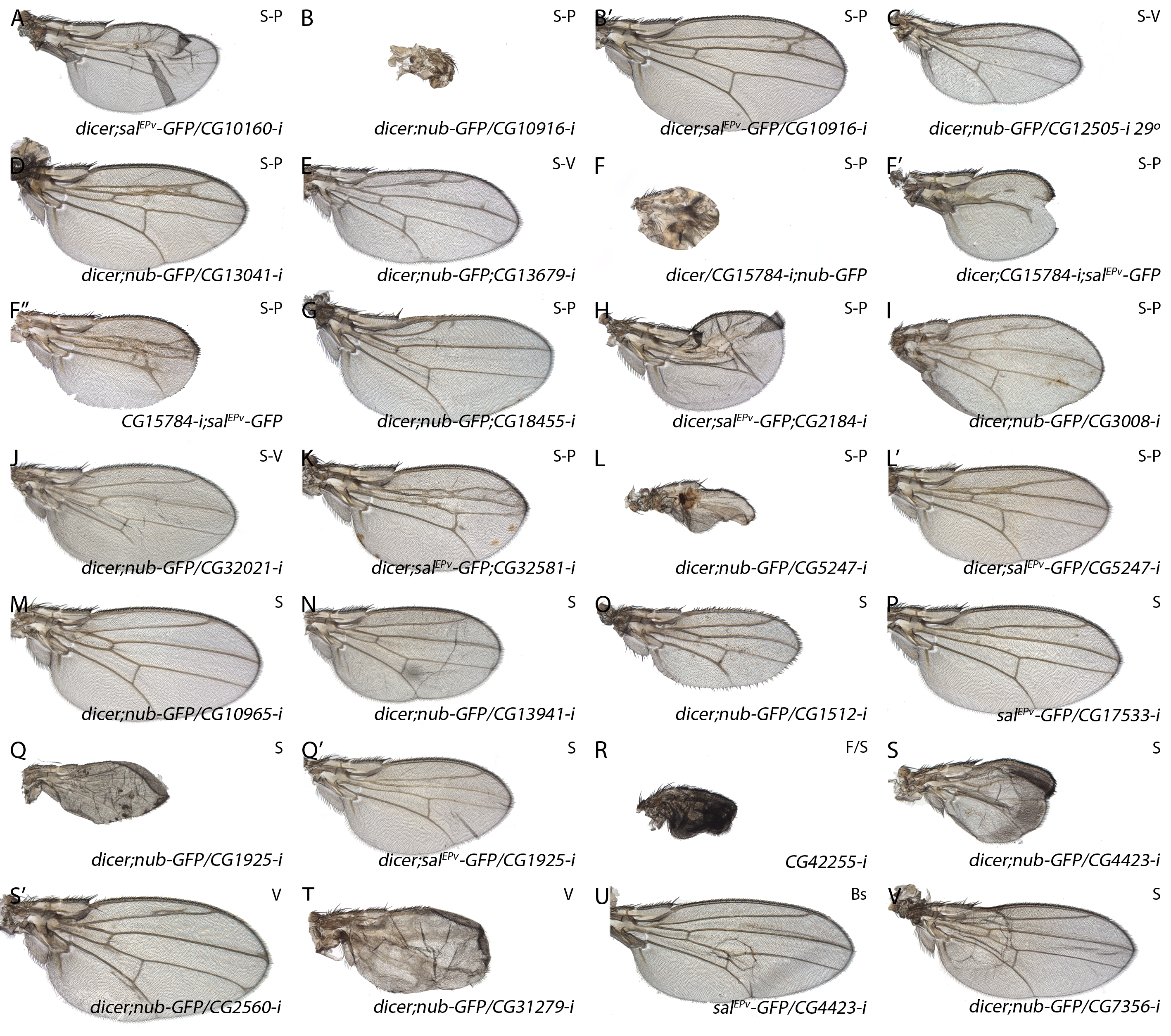

Supplement: S14 Fig — The genotype and the phenotype of each combination are indicated at the bottom and at the top of panels A-V. (TIF) [file pgen.1005370.s014.tif]

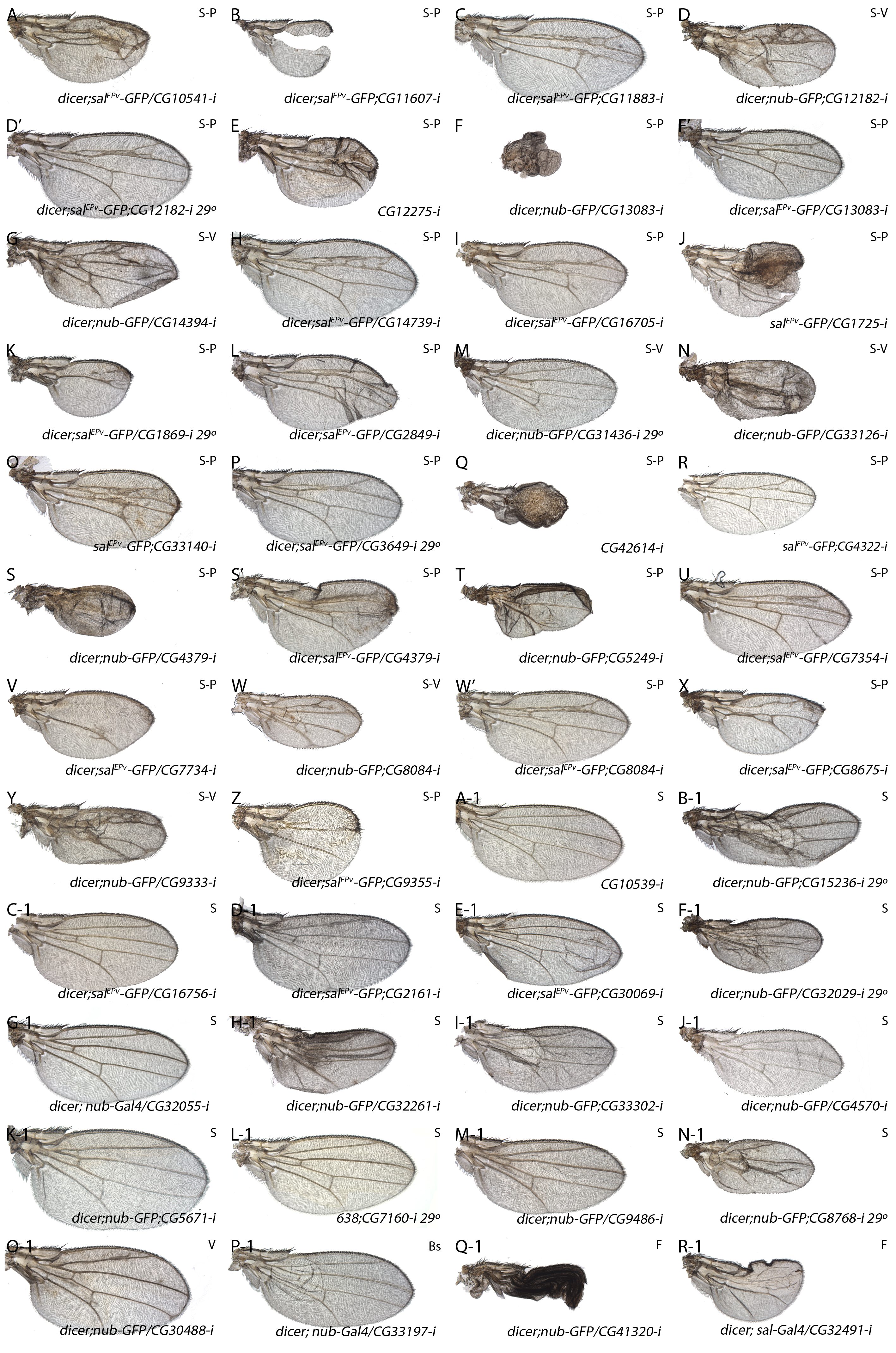

Supplement: S15 Fig — The genotype and the phenotype of each combination are indicated at the bottom and at the top of panels A-R-1. (TIF) [file pgen.1005370.s015.tif]
