# Supplementary material for: The Spalt Transcription Factors Generate the Transcriptional Landscape of the Drosophila melanogaster Wing Pouch Central Region
Source: PLoS Genet. 2015 Aug 4;11(8):e1005370. doi: 10.1371/journal.pgen.1005370 (PMC4524721; doi:10.1371/journal.pgen.1005370)
Supplement: S3 Table — Microarray data of experiment 1 indicating logFoldChange (“logFC”) and adjusted p-value (“adj.P.Val”) at 24h and 48h at 29°C. “cDNA/PCR” indicates the generation of RNA probes using cDNA or genomic DNA (PCR). “In situ” indicates the mRNA expression patterns in wild type and salm-i/salr-i discs (N: expression not detected; U: generalised expression and P: patterned expression). We indicated by “=“, “+” and “-”that the expression does not change, appears ectopic or is reduced in salmi/salri discs, respectively. “Human” indicates the human orthologous. (PDF) [file pgen.1005370.s018.pdf]

Spalt +								
**Name**	**Symbol**	**logFC (48)**	**adj.P.Val (48)**	**logFC (24)**	**adj.P.Val (24)**	**cDNA/PCR**	**In situ**	**Human**
**Age 24h/48h**								
CG1058	*rpk*	-1,20	2,50E-05	0,99	6,11E-04	PCR	P-	ASIC5
CG10965	*Corp*	-1,04	1,29E-08	0,43	5,12E-03	GH26991	U+	
CG11086	*Gadd45*	-2,06	5,86E-11	1,63	6,99E-09	RH70774	U+	GADD45A,B,G
CG11155	*CG11155*	-1,01	1,33E-05	0,71	2,11E-03	PCR	N=	GRIK1-5
CG11584	*CG11584*	-2,84	1,79E-13	2,18	3,37E-11	PCR	U-	POR2AF1
CG11897	*CG11897*	-1,22	1,37E-06	0,95	5,08E-03	PCR	U+	ABCC1-3,5,6,8-12
CG12177	*CG12177*	-1,09	5,14E-05	0,72	9,37E-03	RE18811	U=	
CG1221	*miple*	-1,26	1,51E-10	1,04	4,18E-08	RH10518	U-	
CG12224	*CG12224*	-2,18	7,11E-09	1,73	7,21E-07	PCR	U=	
CG12367	*Hen1*	-1,45	7,16E-09	1,07	1,12E-06	PCR	U=	HENMT1
CG13041	*CG13041*	-1,10	1,17E-05	0,57	2,70E-02	RE40431	U=	
CG13679	*CG13679*	-1,08	2,43E-03	1,08	4,20E-03	RE17113	U-	
CG13937	*CG13937*	-1,44	1,96E-06	1,28	2,45E-05	PCR	U-	CHST8-14
CG14907	*CG14907*	-1,22	9,08E-08	0,52	1,09E-02	LD37858	N+	
CG1512	*Cul-2*	-1,53	6,05E-12	0,44	2,87E-03	LD36177	U+	CUL2
CG15400	*CG15400*	-1,04	1,59E-05	0,98	6,31E-05	PCR	U-	G6PC3/G6PC
CG15784	*CG15784*	-3,98	2,06E-11	3,31	9,87E-10	PCR	P+	
CG15892	*CG15892*	-1,91	8,40E-13	1,80	6,31E-12	PCR	U=	
CG16857	*CG16857*	-1,17	7,89E-04	1,09	2,80E-03	GH01205	U=	
CG17104	*CG17104*	-1,09	1,63E-07	0,47	1,42E-02	PCR	P+	
CG17530	*GstE6*	-3,53	3,03E-12	1,37	5,03E-05	RE21095	N+	GSTT2-2B
CG17533	*GstE8*	-2,03	3,34E-09	1,47	1,51E-06	PCR	U+	GSTT2-2B
CG17574	*CG17574*	-1,09	3,81E-06	0,55	2,30E-04	RH03863	U=	
CG17803	*CG17803*	-1,34	7,23E-06	1,19	7,46E-05	PCR	N=	ZBTB39/ZNF462
CG18188	*Damm*	-1,89	5,28E-09	1,56	1,61E-07	PCR	U=	CASP1,3-7
CG18278	*CG18278*	-0,99	4,96E-06	1,00	9,89E-06	PCR	U=	GNS

Spalt +								
**Name**	**Symbol**	**logFC (48)**	**adj.P.Val (48)**	**logFC (24)**	**adj.P.Val (24)**	**cDNA/PCR**	**In situ**	**Human**
**Age 24h/48h**								
CG18455	*Optix*	-1,17	5,33E-07	1,20	7,58E-07	LD05472	P+	SIX3,6
CG1851	*Ady43A*	-1,14	5,28E-09	0,97	1,67E-07	PCR	U+	ADK
CG18522	*CG18522*	-3,82	5,97E-11	1,46	5,23E-04	LD37006	N+	XDH
CG18543	*mtrm*	-1,03	9,80E-08	0,67	1,39E-04	LD47919	U=	
CG2184	*Mlc2*	-0,93	1,83E-02	1,12	7,83E-03	PCR	N=	MYL 2,5,7,9,10,12A,12B
CG2560	*Cpr11A*	-1,05	8,10E-05	0,56	4,27E-02	RE57452	U=	
CG2909	*CG2909*	-1,27	3,74E-07	0,57	1,55E-02	RE54994	U+	
CG2914	*Ets21C*	-1,12	7,01E-09	0,41	1,23E-02	PCR	U-	ERG/ERF
CG3074	*Swim*	-1,91	1,74E-06	0,79	4,96E-02	PCR	U+	TINAG/TINAGL1
CG3081	*CG3081*	-2,55	2,59E-09	1,67	5,68E-06	PCR	U=	
CG31332	*Unc-115b*	-1,52	1,29E-02	1,42	3,52E-02	SD03267	U=	ABLIM1-3
CG31875	*CG31875*	-1,09	3,10E-06	0,75	8,65E-04	PCR	U=	RCOR1-3
CG32475	*mthl8*	-1,65	7,80E-09	1,57	3,93E-08	LP02895	U=	ELTD1/EMR1-3
CG32581	*CG32581*	-1,22	7,79E-05	1,25	1,05E-04	GH14055	U=	RNF185
CG32625	*CG32625*	-3,04	4,12E-11	0,75	2,61E-02	PCR	U+	GTSF1/GTSF1L
CG32788	*Crg-1*	-1,89	5,97E-11	0,74	1,35E-02	PCR	U=	
CG33048	*Mocs1*	-1,02	6,44E-06	0,70	1,58E-03	PCR	U+	MOCS1
CG33653	*Caps*	-1,77	7,01E-09	1,13	1,65E-04	GH07283	U=	CADPS/CADPS2
CG3397	*CG3397*	-2,20	5,85E-10	1,48	1,06E-06	PCR	U=	
CG34002	*CG34002*	-2,30	1,46E-06	1,04	2,68E-02	PCR	U-	
CG3448	*CG3448*	-1,41	4,36E-09	0,66	7,87E-04	RE59279	U+	
CG3568	*CG3568*	-0,62	2,08E-02	1,10	1,33E-04	AT21585	U=	
CG40300	*ago-03*	-1,21	4,23E-06	0,92	3,94E-04	PCR	N=	PIWIL2
CG40452	*Snap25*	-1,42	3,45E-06	0,61	4,98E-02	PCR	N=	SNAP23,25
CG4174	*CG4174*	-1,55	2,82E-08	0,91	1,40E-05	PCR	U=	P4HA1-3
CG42516	*CG42516*	-1,01	1,76E-06	0,97	1,74E-04	PCR	U-	GTF3C6

Spalt +								
**Name**	**Symbol**	**logFC (48)**	**adj.P.Val (48)**	**logFC (24)**	**adj.P.Val (24)**	**cDNA/PCR**	**In situ**	**Human**
**Age 24h/48h**								
CG42644	*CG42644*	-3,71	2,78E-16	3,23	6,77E-15	AT14183	N=	NEGR1/LSAMP
CG43144	*CG43144*	-3,26	8,47E-15	1,84	3,45E-10	PCR	N+	
CG43646	*CG43646*	-1,50	3,51E-07	1,22	1,79E-05	PCR	U=	
CG43676	*CG43676*	-1,55	2,57E-09	1,31	8,51E-08	PCR	N=	
CG4594	*CG4594*	-3,06	2,21E-11	2,96	7,33E-11	PCR	U-	ECI1
CG4920	*ea*	-1,01	1,54E-04	1,07	1,28E-04	RE55124	U=	TPSG1/TPSAB1
CG5022	*CG5022*	-1,25	2,32E-05	1,32	2,29E-05	RE05264	U=	FRMD3,5
CG5096	*CG5096*	-2,26	7,04E-09	2,19	2,21E-08	RE59088	U+	SLITRK1,3-5
CG5229	*chm*	-2,12	1,15E-07	1,75	5,38E-06	PCR	U-	KAT7
CG5966	*CG5966*	-1,19	1,17E-05	0,66	1,60E-02	LD47264	P=	
CG6658	*Ugt86Di*	-1,53	1,14E-09	1,21	1,48E-07	PCR	P+	UGT1A10
CG6665	*CG6665*	-1,40	1,55E-09	1,23	2,87E-08	SD03555	U=	C1orf43
CG6794	*Dif*	-1,96	4,74E-08	1,12	4,00E-04	RE18723	U+	REL/RELA,B
CG6912	*CG6912*	-1,87	1,63E-07	1,76	1,06E-06	LD45181	U=	
CG7035	*Cbp80*	-1,17	1,28E-07	1,13	4,70E-07	LD31211	U=	NCBP1
CG7590	*scyl*	-0,95	3,61E-02	1,22	1,17E-02	PCR	U+	DDIT4/DDIT4L
CG7787	*CG7787*	-1,05	5,06E-05	0,86	1,05E-03	RE45155	U=	RABIF
CG8573	*su(Hw)*	-1,02	9,02E-09	1,03	1,58E-08	PCR	U=	ZNF443/ZNF678
CG8620	*CG8620*	-3,54	2,50E-13	0,92	9,84E-04	RH55750	U-	
CG8799	*l(2)03659*	-1,56	1,28E-07	0,99	2,43E-04	PCR	U=	ABCC1-3,5,6,8-12
CG9214	*Tob*	-1,81	4,20E-05	1,16	9,79E-03	PCR	U=	TOB1-2
**Age 48h**								
CG10160	*ImpL3*	-1,18	4,61E-03	--------------	--------------	--------------	----------	LDHA-C/LDHAL6A,B
CG10245	*Cyp6a20*	-1,90	3,06E-10	--------------	--------------	--------------	----------	
CG1155	*Osi14*	-1,44	5,40E-05	--------------	--------------	LD24139	P=	
CG11592	*Amnionless*	-1,10	9,16E-03	--------------	--------------	--------------	----------	AMN

Spalt+								
**Name**	**Symbol**	**logFC (48)**	**adj.P.Val (48)**	**logFC (24)**	**adj.P.Val (24)**	**cDNA/PCR**	**In situ**	**Human**
				**Age 48h**				
CG12242	*GstD5*	-4,15	9,59E-14	--------------	--------------	--------------	----------	
CG12505	*Arc1*	-3,91	4,31E-09	--------------	--------------	LD41905	N+	
CG12846	*Tsp42Ed*	-1,95	2,82E-08	--------------	--------------	--------------	----------	TSPAN7/CD63
CG12908	*Ndg*	-1,26	1,68E-07	--------------	--------------	--------------	----------	
CG1299	*CG1299*	-1,07	2,60E-06	--------------	--------------	--------------	----------	TPSG1/TPSAB1
CG13060	*CG13060*	-1,25	7,94E-05	--------------	--------------	--------------	----------	
CG13941	*Arc2*	-1,55	2,61E-06	--------------	--------------	--------------	----------	
CG14059	*Ilp8*	-4,55	3,55E-08	--------------	--------------	--------------	----------	
CG14196	*CG14196*	-1,30	9,96E-04	--------------	--------------	--------------	----------	SLC16A1,3,5,7,8,12
CG14304	*CG14304*	-1,09	1,54E-04	--------------	--------------	--------------	----------	
CG14545	*CG14545*	-1,85	2,21E-10	--------------	--------------	--------------	----------	
CG14695	*CG14695*	-1,46	6,54E-08	--------------	--------------	--------------	----------	
CG15209	*CG15209*	-1,08	1,61E-03	--------------	--------------	--------------	----------	
CG15347	*CG15347*	-2,15	1,29E-04	--------------	--------------	--------------	----------	
CG15623	*c-cup*	-1,25	1,19E-05	--------------	--------------	--------------	----------	CYB5D2
CG18859	*Or19a*	-2,14	5,80E-08	--------------	--------------	--------------	----------	
CG2065	*CG2065*	-1,35	3,41E-04	--------------	--------------	--------------	----------	RDH11
CG2885	*RabX2*	-2,13	6,54E-10	--------------	--------------	--------------	----------	RAB10,12
CG30196	*CG30196*	-1,73	1,53E-05	--------------	--------------	PCR	N+	
CG31062	*side*	-1,19	4,27E-07	--------------	--------------	--------------	----------	
CG31279	*CG31279*	-2,50	5,95E-12	--------------	--------------	--------------	----------	
CG32825	*Or19b*	-1,76	1,58E-07	--------------	--------------	--------------	----------	
CG34031	*CG34031*	-1,08	1,93E-02	--------------	--------------	--------------	----------	
CG34165	*CG34165*	-1,23	3,45E-09	--------------	--------------	--------------	----------	
CG3767	*JhI-26*	-2,23	3,06E-10	--------------	--------------	--------------	----------	
CG4181	*GstD2*	-2,46	1,55E-08	--------------	--------------	--------------	----------	

Spalt +								
**Name**	**Symbol**	**logFC (48)**	**adj.P.Val (48)**	**logFC (24)**	**adj.P.Val (24)**	**cDNA/PCR**	**In situ**	**Human**
				**Age 48h**				
CG42255	*CG42255*	-1,50	2,89E-02	--------------	--------------	--------------	----------	CUBN
CG42352	*Ir40a*	-2,02	1,01E-10	--------------	--------------	--------------	----------	GRIK1-4/GRID1,2
CG42363	*CG42363*	-1,25	4,75E-03	--------------	--------------	--------------	----------	
CG4373	*Cyp6d2*	-1,81	4,33E-04	--------------	--------------	RE31155	U	
CG4423	*GstD6*	-1,85	3,45E-09	--------------	--------------	--------------	----------	
CG5955	*CG5955*	-2,70	8,47E-15	--------------	--------------	--------------	----------	
CG6042	*Cyp12a4*	-1,17	9,84E-04	--------------	--------------	--------------	----------	
CG6142	*CG6142*	-1,14	1,81E-02	--------------	--------------	--------------	----------	CHDH
CG7080	*CG7080*	-1,16	1,19E-04	--------------	--------------	PCR	P-	
CG7356	*Tg*	-1,27	1,41E-06	--------------	--------------	--------------	----------	TGM1-7
CG8927	*CG8927*	-2,11	1,29E-08	--------------	--------------	--------------	----------	
CG9521	*CG9521*	-1,01	2,49E-04	--------------	--------------	--------------	----------	CHDH
CG9877	*CG9877*	-1,33	1,11E-02	--------------	--------------	--------------	----------	
				**Age 24h**				
CG11486	*CG11486*	--------------	--------------	0,90	3,38E-02	PCR	U=	PAN3
CG13316	*Mnt*	--------------	--------------	0,90	3,86E-02	GH28809	U-	MNT
CG30059	*CG30059*	--------------	--------------	1,01	2,35E-05	SD03412	U=	POLR3A
CG32019	*bt*	--------------	--------------	1,18	5,27E-03	GH07636	N=	PALLD/IGFN1
CG45050	*CG43674*	--------------	--------------	0,95	2,15E-02	PCR	U+	
CG6544	*fau*	--------------	--------------	1,27	4,17E-02	HL07933	U=	
CG7178	*wupA*	--------------	--------------	1,31	2,16E-02	PCR	N=	TNNI1-3
CG8663	*nrv3*	--------------	--------------	1,26	2,13E-03	RH24769	N=	ATP1B1-4

Spalt +

Df//UAS

Name	Symbol	Genotype				cDNA/PCR	In situ	Human
CG10102	*CR10102/Arc3*	UAS	--------------	--------------	--------------	PCR	N+	
CG10916	*CG10916*	Df//UAS	--------------	--------------	--------------	LD46221	U+	TRAIP
CG1303	*agt*	Df//UAS	--------------	--------------	--------------	PCR	U+	
CG14121	*l(3)S147910/ve*	Df//UAS	--------------	--------------	--------------	PCR	U+	
CG15009	*ImpL2*	UAS	--------------	--------------	--------------	SD07266	P+	IGSF9-B
CG16928	*mre11*	Df//UAS	--------------	--------------	--------------	PCR	N+	MRE11A
CG18213	*CG18213*	Df//UAS	--------------	--------------	--------------	LD11102	N+	SMYD1-4
CG1925	*mus205*	Df	--------------	--------------	--------------	PCR	U+	POLD1/KIAA2022/POLA1/REV3L
CG2999	*unc-13*	Df//UAS	--------------	--------------	--------------	PCR	P+	UNC13A-C
CG3008	*CG3008*	UAS	--------------	--------------	--------------	RE33807	U+	RIOK1-3
CG31705	*CG31705*	UAS	--------------	--------------	--------------	GH07269	U+	
CG32021	*CG32021*	Df//UAS	--------------	--------------	--------------	SD15619	N+	
CG5202	*escl*	UAS	--------------	--------------	--------------	SD02661	U+	EED
CG5247	*Irbp*	UAS	--------------	--------------	--------------	RE28533	U+	XRCC6
CG6272	*CG6272*	Df//UAS	--------------	--------------	--------------	GH10915	N+	CEBPA-B-D-E-G

Spalt -

Name	Symbol	logFC (48)	adj.P.Val (48)	logFC (24)	adj.P.Val (24)	cDNA/PCR	In situ	Human
				**Age 24h/48h**				
CG10344	*CG10344*	1,10	2,79E-04	-1,12	4,32E-04	AT16443	U=	ZDHHC24
CG10539	*S6k*	1,03	1,95E-05	-0,70	3,63E-03	RE65159	U=	RPS6KB1
CG10541	*Tektin-C*	4,93	1,49E-13	-5,10	1,95E-13	AT25102	U=	TEKT3-5
CG10576	*CG10576*	1,16	5,83E-07	-1,22	5,02E-07	PCR	P-	PA2G4

Spalt -								
**Name**	**Symbol**	**logFC (48)**	**adj.P.Val (48)**	**logFC (24)**	**adj.P.Val (24)**	**cDNA/PCR**	**In situ**	**Human**
				**Age 24h/48h**				
CG11253	*CG11253*	1,05	1,02E-04	-1,11	4,86E-05	AT27448	U=	ZMYND10
CG11263	*CG11263*	0,85	1,43E-06	-1,03	1,42E-07	PCR	U-	EXD1
CG12182	*CG12182*	1,07	8,01E-10	-0,96	1,24E-08	GH02340	U-	
CG12275	*RpS10a*	1,08	4,35E-05	-0,43	3,28E-02	LD32148	U=	RSP10
CG12843	*Tsp42Ei*	1,27	1,76E-06	-0,87	6,92E-04	RE08220	P-	TSPAN18/CD53
CG13083	*CG13083*	1,54	6,09E-08	-1,31	1,84E-06	PCR	U-	
CG1342	*Spn100A*	1,46	1,48E-03	-1,44	2,83E-03	LP03106	P-	SERPINA-I
CG13890	*CG13890*	1,07	1,42E-08	-1,28	1,21E-09	PCR	U-	CYD1-2B
CG13897	*CG13897*	1,05	2,23E-05	-1,17	9,97E-06	RE38876	U-	
CG14394	*NijC*	2,63	4,94E-13	-2,34	8,12E-12	PCR	U-	NINJ1-2
CG14485	*swi2*	0,65	4,96E-02	-1,05	2,49E-03	PCR	U-	
CG14715	*CG14715*	1,67	5,97E-11	-1,84	2,37E-11	RH50927	U=	FKBP2,7,9,14
CG15236	*CG15236*	1,33	3,49E-07	-1,74	7,22E-09	RE34115	U=	
CG15707	*krimp*	1,50	9,07E-05	-1,79	1,51E-05	LD30829	U=	TDRD7,1,6,15
CG17077	*pnt*	1,18	9,37E-07	-1,26	6,35E-07	PCR	U=	ETS1,2
CG2161	*Rga*	1,07	5,42E-06	-1,19	1,96E-06	GM14102	U=	CNOT2
CG2849	*Rala*	2,99	1,49E-13	-3,04	1,95E-13	LD21679	U=	RALA/RALB
CG30104	*CG30104*	1,04	2,89E-02	-1,54	2,16E-03	LP01187	U-	NT5E
CG30488	*CG30488*	1,37	4,75E-10	-1,47	3,00E-10	PCR	U=	
CG31262	*CG31262*	1,05	5,17E-04	-1,12	2,61E-03	PCR	U=	SLC5A5,6,8,12
CG31436	*CG31436*	2,60	5,86E-11	-2,99	8,50E-12	PCR	U-	
CG31809	*CG31809*	1,57	2,57E-10	-1,76	6,96E-11	RE06583	N=	HSDL1-17B
CG31810	*CG31810*	1,31	7,16E-09	-1,54	8,12E-10	RE06583	N=	HSDL1-17B
CG32364	*CG32364*	1,81	8,14E-09	-2,31	2,36E-10	PCR	U-	
CG33140	*CG33140*	1,61	1,63E-08	-1,43	2,93E-07	PCR	U=	
CG33302	*Cpr31A*	1,14	1,60E-05	-1,46	6,35E-07	PCR	U-	

Spalt -								
**Name**	**Symbol**	**logFC (48)**	**adj.P.Val (48)**	**logFC (24)**	**adj.P.Val (24)**	**cDNA/PCR**	**In situ**	**Human**
**Age 24h/48h**								
CG34215	*CG34215*	2,87	3,04E-09	-2,83	5,81E-09	PCR	U=	
CG3649	*CG3649*	1,50	1,43E-06	-1,61	9,25E-07	PCR	P=	SLC17A1-5/SLC37A1-2
CG40178	*CG40178*	1,17	4,59E-08	-1,14	1,53E-07	PCR	U=	DNAJC16
CG4086	*Su(P)*	1,74	1,04E-07	-1,58	1,11E-06	RH17614	U=	PTGES2
CG41320	*CG41320*	1,35	9,32E-07	-1,28	4,37E-06	PCR	U-	DNAJC16
CG42330	*Dscam4*	3,84	8,40E-13	-3,66	5,59E-12	PCR	U-	MANY
CG43079	*nrm*	1,34	2,22E-06	-1,13	1,32E-05	PCR	P=	HL05969
CG43103	*CG43103*	2,02	3,22E-06	-1,84	2,54E-05	PCR	U=	
CG43117	*CG43117*	1,34	3,67E-07	-1,19	4,98E-05	PCR	U-	
CG4570	*CG4570*	1,82	7,09E-08	-2,15	8,91E-09	PCR	U-	SLC40A1
CG5671	*Pten*	4,00	4,75E-12	-4,00	8,50E-12	PCR	U=	PTEN
CG6013	*CG6013*	1,04	1,86E-06	-1,18	5,02E-07	GH10002	P-	CCDC124
CG7160	*Cpr78E*	1,88	8,30E-03	-1,47	4,06E-02	LP06027	U-	
CG7354	*mRpS26*	0,69	1,28E-02	-1,04	4,34E-04	RE31184	U=	MRPS26
CG7478	*Act79B*	1,29	5,54E-08	-1,26	1,59E-07	GH04529	U-	ACT A1-C1
CG7532	*l(2)34Fc*	0,94	2,07E-03	-1,63	2,91E-06	RE60882	U-	
CG7607	*CG7607*	1,54	2,84E-07	-1,61	2,98E-07	RH68451	U=	CDON/BOC/TMIGD1
CG7916	*CG7916*	1,06	5,15E-04	-0,83	1,00E-02	RE38722	U=	
CG8023	*eIF4E-3*	1,39	3,09E-07	-1,35	1,10E-06	PCR	U-	EIF4E
CG8768	*CG8768*	1,07	3,10E-06	-0,98	2,20E-05	PCR	U-	SDR39U1
CG9333	*Oseg5*	2,42	1,80E-09	-2,34	4,67E-10	PCR	U-	IFT80
**Age 48h**								
CG11051	*Nplp2*	1,31	4,82E-02	--------------	--------------	--------------	----------	
CG14332	*CG14332*	2,07	4,57E-02	--------------	--------------	--------------	----------	
CG17876	*Amy-d*	1,26	4,42E-02	--------------	--------------	--------------	----------	AMY1B-2B
CG18363	*dic-04*	1,19	2,52E-02	--------------	--------------	--------------	----------	SLC25A10

Spalt -

Name	Symbol	logFC (48)	adj.P.Val (48)	logFC (24)	adj.P.Val (24)	cDNA/PCR	In situ	Human
				**Age 48h**				
CG33126	*NLaz*	1,27	1,08E-02	--------------	--------------	--------------	----------	APOD
CG42798	*CG42798*	1,91	2,12E-02	--------------	--------------	--------------	----------	
CG42834	*CG42834*	2,27	1,70E-02	--------------	--------------	--------------	----------	
CG9486	*CG9486*	1,50	1,45E-02	--------------	--------------	--------------	----------	
				**Age 24h**				
CG13053	*CG13053*	--------------	--------------	-1,07	2,40E-03	LP04080	U-	
CG17134	*CG17134*	--------------	--------------	-1,10	1,12E-02	PCR	U-	REN
CG2360	*Ccp84Aa*	--------------	--------------	-1,02	4,56E-02	RH13984	U=	
CG3200	*Reg-2*	--------------	--------------	-0,99	8,01E-03	PCR	U-	HDHD3
CG32444	*CG32444*	--------------	--------------	-1,12	4,32E-03	PCR	U-	GALM
CG43114	*CG43114*	--------------	--------------	-0,95	4,23E-02	PCR	U-	
CG6579	*atilla*	--------------	--------------	-0,92	1,18E-02	PCR	N=	
CG8800	*CG8800*	--------------	--------------	-1,01	2,73E-02		U=	DNAL1/LRRC61
				**Df//UAS**				
**Name**	**Symbol**	**Genotype**				**cDNA/PCR**	**In situ**	**Human**
CG10041	*CG10041*	UAS	--------------	--------------	--------------	PCR	U-	
CG10225	*RanBP3*	Df	--------------	--------------	--------------	PCR	U-	RANBP3-3L
CG10382	*wrapper*	UAS	--------------	--------------	--------------	PCR	U-	NEGR1/LSAMP
CG11212	*Ptr*	UAS	--------------	--------------	--------------	PCR	U-	PTCHD4,1
CG11357	*CG11357*	Df	--------------	--------------	--------------	PCR	U-	B3GALT5,1,2
CG11607	*H2.0*	UAS	--------------	--------------	--------------	RE37758	U-	HLX
CG11797	*Obp56a*	UAS	--------------	--------------	--------------	PCR	U-	
CG11883	*CG11883*	UAS	--------------	--------------	--------------	PCR	U-	NT5E
CG12052	*lola*	UAS	--------------	--------------	--------------	PCR	U-	

Spalt -								
**Df//UAS**								
**Name**	**Symbol**	**Genotype**				**cDNA/PCR**	**In situ**	**Human**
CG12256	*CG12256*	UAS	--------------	--------------	--------------	PCR	U-	KLK1-5,7,8,11,12,14
CG12287	*pdm2*	UAS	--------------	--------------	--------------	PCR	U-	HDX/POU2F1-3
CG1273	*CG1273*	UAS	--------------	--------------	--------------	RE03558	P-	FNDC1/ABI3BP
CG12952	*sage*	Df	--------------	--------------	--------------	PCR	U-	MESP1-2/MSGN1
CG13618	*CG13618*	UAS	--------------	--------------	--------------	RE08075	U-	
CG13848	*pinta*	Df//UAS	--------------	--------------	--------------	PCR	U-	TTPAL
CG14590	*CG14590*	UAS	--------------	--------------	--------------	PCR	U-	SMYD1-4
CG14869	*CG6107*	Df	--------------	--------------	--------------	PCR	U-	ADAMTS1-5,8,9,14,15,20
CG15369	*CG15369*	Df	--------------	--------------	--------------	IP05946	U-	
CG15739	*CG15739*	Df	--------------	--------------	--------------	PCR	U-	
CG16705	*SPE*	Df	--------------	--------------	--------------	GH28857	U-	
CG16712	*CG16712*	Df	--------------	--------------	--------------	RH38008	U-	TFPI2/WFDC8
CG16756	*CG16756*	Df	--------------	--------------	--------------	PCR	U-	LYZL1,2,4,6
CG16947	*CG16947*	Df//UAS	--------------	--------------	--------------	PCR	U-	RCHY1
CG1725	*dlg1*	Df	--------------	--------------	--------------	PCR	U-	DLG1-4
CG1763	*nod*	UAS	--------------	--------------	--------------	PCR	U-	KIF11
CG1780	*Idgf4*	Df//UAS	--------------	--------------	--------------	PCR	U-	OVGP1/CHIT1
CG18024	*SoxN*	Df	--------------	--------------	--------------	PCR	U-	SOX1-3,14,15,24
CG18255	*Strn-Mlck*	Df	--------------	--------------	--------------	PCR	U-	NEXN
CG18410	*Ude*	UAS	--------------	--------------	--------------	PCR	U-	
CG18657	*NetA*	UAS	--------------	--------------	--------------	PCR	P-	NTN1,3/MEGF8
CG1869	*Cht7*	Df	--------------	--------------	--------------	LD45559	P-	OVGP1/CHIT1/CHHI3L1,2
CG2056	*spirit*	Df	--------------	--------------	--------------	PCR	U-	
CG2488	*phr6-4*	Df	--------------	--------------	--------------	PCR	U-	
CG2706	*fs(1)Yb*	Df//UAS	--------------	--------------	--------------	PCR	U-	
Spalt -								

Df//UAS

Name	Symbol	Genotype				cDNA/PCR	In situ	Human
CG30069	*CG30069*	Df	--------------	--------------	--------------	LP06813	P-	POLR3A
CG3097	*CG3097*	UAS	--------------	--------------	--------------	RE43153	U-	CPO/CPA2-4,6/CPB1,2
CG3100	*b6*	UAS	--------------	--------------	--------------	PCR	U-	
CG31098	*CG10634*	Df//UAS	--------------	--------------	--------------	PCR	U-	
CG31721	*Trim9*	UAS	--------------	--------------	--------------	RE22018	P-	
CG31764	*vir-1*	Df//UAS	--------------	--------------	--------------	GH02216	P-	
CG31839	*nimB2*	Df	--------------	--------------	--------------	PCR	U-	
CG31884	*Trx-2*	UAS	--------------	--------------	--------------	SD03042	U-	TXNDC8,2/TXN
CG32029	*Cpr66D*	Df//UAS	--------------	--------------	--------------	RE57183	U-	
CG32055	*CG32055*	Df//UAS	--------------	--------------	--------------	PCR	P-	LRRN4CL/LRRN4/ISLR
CG32261	*Gr64a*	UAS	--------------	--------------	--------------	PCR	U-	
CG3240	*Rad1*	Df	--------------	--------------	--------------	PCR	U-	RAD1
CG32491	*mod(mdg4)*	Df	--------------	--------------	--------------	PCR	U-	
CG3289	*Ptpa*	Df	--------------	--------------	--------------	PCR	U-	PPP2R4
CG33197	*mbl*	Df//UAS	--------------	--------------	--------------	RE67734	U-	
CG33960	*Sema-2b*	UAS	--------------	--------------	--------------	PCR	U-	SEMA4A-D,F,G
CG33970	*CG33970*	UAS	--------------	--------------	--------------	GH14066	P-	
CG34406	*CG44002*	UAS	--------------	--------------	--------------	PCR	U-	
CG42614	*scrib*	UAS	--------------	--------------	--------------	PCR	U-	
CG4322	*moody*	UAS	--------------	--------------	--------------	RE06985	U-	
CG4379	*Pka-C1*	UAS	--------------	--------------	--------------	PCR	U-	PRKX/PRKACG,B,A/PRKG1,2
CG4398	*CG4398*	UAS	--------------	--------------	--------------	PCR	U-	
CG4432	*PGRP-LC*	Df	--------------	--------------	--------------	PCR	U-	PGLYRP1-4
CG4565	*CG4565*	UAS	--------------	--------------	--------------	PCR	U-	SETDB1,2/ASH1L/EHMT1
CG5249	*Blimp-1*	UAS	--------------	--------------	--------------	PCR	U-	
CG6134	*spz*	Df	--------------	--------------	--------------	PCR	U-	

Spalt -

Df//UAS								
**Name**	**Symbol**	**Genotype**				**cDNA/PCR**	**In situ**	**Human**
CG6713	*Nos*	Df	--------------	--------------	--------------	PCR	U-	NOS1-3
CG7201	*CG7201*	UAS	--------------	--------------	--------------	PCR	P-	
CG7420	*CG7420*	Df	--------------	--------------	--------------	PCR	U-	
CG7577	*ppk20*	Df//UAS	--------------	--------------	--------------	PCR	U-	ASIC5
CG7734	*shn*	UAS	--------------	--------------	--------------	PCR	U-	HIVEP1-3/ZNF831
CG7897	*gp210*	Df	--------------	--------------	--------------	PCR	U-	
CG7906	*CG7906*	Df	--------------	--------------	--------------	PCR	U-	
CG8084	*ana*	UAS	--------------	--------------	--------------	GH07389	P-	
CG8589	*tej*	UAS	--------------	--------------	--------------	PCR	U-	AKAP1
CG8675	*CG8675*	Df	--------------	--------------	--------------	LD08644	U-	C9orf85
CG8773	*CG8773*	UAS	--------------	--------------	--------------	PCR	U-	ENPEP
CG8780	*tey*	UAS	--------------	--------------	--------------	PCR	U-	RNF220
CG8945	*CG8945*	Df	--------------	--------------	--------------	PCR	U-	CPO/CPA2-4,6/CPB1,2
CG9355	*dy*	UAS	--------------	--------------	--------------	LP04610	U-	
CG9460	*Spn42De*	Df	--------------	--------------	--------------	PCR	U-	SERPINC1/SERPINB1-13
CG9850	*CG11169*	Df//UAS	--------------	--------------	--------------	LP02257	P-	ADAMTS1-5,8,9,14,15,20
