# Supplementary material for: The Spalt Transcription Factors Generate the Transcriptional Landscape of the Drosophila melanogaster Wing Pouch Central Region
Source: PLoS Genet. 2015 Aug 4;11(8):e1005370. doi: 10.1371/journal.pgen.1005370 (PMC4524721; doi:10.1371/journal.pgen.1005370)
Supplement: S5 Table — “Phenotype” indicates the phenotype of nub-Gal4/UAS-RNA-i and sal EPv -Gal4/UAS-RNA-i separated by double dash: + (WT); S (wing size); P (vein pattern); S-P (wing size and vein pattern); V+ and V- (extra-veins and loss of veins); nW (no wing); F (folded); Bs (dorso-ventral wing surface adhesion); N (loss of wing margin structures); L, EPL and PL (lethal, early pupal lethal and pupal lethal); CD (cell death) and Cell. Pig. (Cellular pigmentation). The phenotypes with a small letter “s” or “w” indicate whether the phenotype is strong (s) or weak (w). “Molecular” indicates the molecular nature and simplified molecular class, respectively: D (genes related with the biology of DNA), P (genes related with proteins metabolism), CG (genes without known functional domains or orthology), CS (genes encoding components of signalling pathways), CGh (genes with a known functional domain but not clear orthology relationships), M (genes encoding proteins related to the metabolism of lipids or glucids), CA+Cyt (genes related with cell adhesion or the cytoskeleton), Cut (genes encoding proteins related with structural constituent of cuticle), R (genes related with the biology of the RNA), Tra (genes related with the transport of metabolites across cellular membranes), RedOx (genes encoding proteins related with oxidation-reduction process), CD (genes related with cell death), Neu (genes encoding neurotransmitters), CR (non protein coding gene) and CDiv (genes related with cell division). (PDF) [file pgen.1005370.s020.pdf]

Spalt +					
**Name**	**Symbol**	**RNAi strain**	**Phenotype**	**Molecular**	**Molecular**
				**Age 24h/48h**	
CG1058	*rpk*	1058R-1	+	Ligand-gated sodium channel	Neu
CG10965	*Corp*	102751-KK	wS	Apoptotic signaling pathway in response to DNA damage	CD
CG11086	*Gadd45*	BL-35023	+	JNK cascade	CS
CG11155	*CG11155*	BL-31991	+	Glutamate receptor	Neu
CG11584	*CG11584*	--------------	--------------	--------------	CG
CG11897	*CG11897*	28260-GD	+	ABC transporter	Tra
CG12177	*CG12177*	17018-GD	+	Inosine/uridine-preferring nucleoside hydrolase	M
CG1221	*miple*	1221R-1	+	Pleiotrophin/Midkine heparin-binding growth factor	CS
CG12224	*CG12224*	26497-GD	+	Oxidoreductase	RedOx
CG12367	*Hen1*	31920-GD	+	RNA methylation	R
CG13041	*CG13041*	109222-KK	S-P//S-P	Retinin-like	CGh
CG13679	*CG13679*	49149-GD	V+/S	--------------	CG
CG13937	*CG13937*	3373-GD	+	Carbohydrate sulfotransferase	M
CG14907	*CG14907*	39851-GD	+	--------------	CG
CG1512	*Cul-2*	100540-KK	sS	Ubiquitin protein ligase	P
CG15400	*CG15400*	7261-GD	+	Glucose-6-phosphatase	M
CG15784	*CG15784*	15784R-3	nW/sN//S-P	--------------	CG
CG15892	*CG15892*	BL-42945	+	--------------	CG
CG16857	*CG16857*	BL-31974	+	Fibronectin, type III; Immunoglobulin I-set	CA
CG17104	*CG17104*	17104R-3	+	--------------	CG
CG17530	*GstE6*	17530R-1	+	Glutathione S-transferase	RedOx
CG17533	*GstE8*	26497-GD	PL-nW//S	Glutathione transferase	RedOx
CG17574	*CG17574*	102094-KK	+	--------------	CG
CG17803	*CG17803*	BL-29367	+	C2H2-type/integrase DNA-binding domain	D
CG18188	*Damm*	109520-KK	sF//+	Cysteine-type endopeptidase	P
CG18278	*CG18278*	BL-28520	+	N-acetylglucosamine-6-sulfatase	M

Spalt +					
**Name**	**Symbol**	**RNAi strain**	**Phenotype**	**Molecular**	**Molecular**
			**Age 24h/48h**		
CG18455	*Optix*	18455R-2	S-P	Sequence-specific DNA binding	D
CG1851	*Ady43A*	33133-GD	+	Carbohydrate kinase PfkB	M
CG18522	*CG18522*	100330-KK	+	Oxidoreductase	RedOx
CG18543	*mtrm*	BL-35430	+	Meiotic cell cycle	CDiv
CG2184	*Mlc2*	BL-36694	EPL//S-P	Microfilament motor	Cyt
CG2560	*Cpr11A*	103321-KK	V+	Cuticle	Cut
CG2909	*CG2909*	100165-KK	V+	--------------	CG
CG2914	*Ets21C*	106153-KK	+	DNA binding transcription factor	D
CG3074	*Swim*	BL-36591	+	Wnt-protein binding	CS
CG3081	*CG3081*	102507-KK	sF//+	--------------	CG
CG31332	*Unc-115b*	BL-40839	+	Actin binding	Cyt
CG31875	*CG31875*	104638-KK	+	Chromatin binding	D
CG32475	*mthl8*	4071-GD	+	G-protein coupled receptor signaling	CS
CG32581	*CG32581*	49728-GD	EPL//S-P	Zinc finger, RING-type	CGh
CG32625	*CG32625*	103216-KK	+	CHHC zinc finger	CGh
CG32788	*Crg-1*	BL-46971	+	Sequence-specific DNA binding	D
CG33048	*Mocs1*	--------------	--------------	Mo-molybdopterin cofactor biosynthetic	M
CG33653	*Caps*	BL-31984	+	Phospholipid binding	Neu
CG3397	*CG3397*	BL-25818	+	Oxidoreductase	RedOx
CG34002	*CG34002*	--------------	--------------	V5/Tpx-1-related	CGh
CG3448	*CG3448*	26198-GD	+	DNA repair protein XRCC4	D
CG3568	*CG3568*	18447-GD	+	--------------	CG
CG40300	*ago-03*	35232-GD	+	mRNA poly(A) tail shortening	R
CG40452	*Snap25*	BL-34377	+	Target SNARE	Neu
CG4174	*CG4174*	BL-36845	+	Procollagen-proline 4-dioxygenase	P
CG42516	*CG42516*	--------------	--------------	Transcription factor TFIIIC	D

Spalt +					
**Name**	**Symbol**	**RNAi strain**	**Phenotype**	**Molecular**	**Molecular**
			**Age 24h/48h**		
CG42644	*CG42644*	--------------	--------------	--------------	CG
CG43144	*CG43144*	--------------	--------------	Non protein coding gene	CR
CG43646	*CG43646*	--------------	--------------	--------------	CG
CG43676	*CG43676*	--------------	--------------	--------------	CG
CG4594	*CG4594*	100448-KK	+	Dodecenoyl-CoA delta-isomerase	M
CG4920	*ea*	BL-41961	+	Serine-type endopeptidase	P
CG5022	*CG5022*	BL-33359	+	Ezrin/radixin/moesin like	Cyt
CG5096	*CG5096*	100951-KK	+	Leucine-rich repeat	CGh
CG5229	*chm*	BL-36869	wS	H4 histone acetyltransferase	D
CG5966	*CG5966*	BL-33932	+	Triglyceride lipase	M
CG6658	*Ugt86Di*	104262-KK	+	Glucuronosyltransferase	M
CG6665	*CG6665*	104625-KK	+	NICE-3	CGh
CG6794	*Dif*	30579-GD	+	DNA binding transcription factor	D
CG6912	*CG6912*	102806-KK	+	--------------	CG
CG7035	*Cbp80*	BL-33648	+	RNA interference	R
CG7590	*scyl*	25506-GD	+	Negative regulation of growth; response to DNA damage	CD
CG7787	*CG7787*	BL-32843	+	Guanyl-nucleotide exchange factor	Tra
CG8573	*su(Hw)*	BL-33906	+	Negative regulation of chromatin silencing	D
CG8620	*CG8620*	BL-42638	+	--------------	CG
CG8799	*l(2)03659*	100105-KK	+	ATPase, transporter	Tra
CG9214	*Tob*	108824-KK	+	Anti-proliferative	CS
			**Age 48h**		
CG10160	*ImpL3*	10160R-1	S-P//S-P	Glycolysis	M
CG10245	*Cyp6a20*	3313-GD	+	Electron carrier	RedOx
CG1155	*Osi14*	102979-KK	+	DUF1676	CGh
CG11592	*Amnionless*	BL-41956	+	Amnionless	CGh

Spalt +					
**Name**	**Symbol**	**RNAi strain**	**Phenotype**	**Molecular**	**Molecular**
			**Age 48h**		
CG12242	*GstD5*	BL-42842	+	Glutathione transferase	RedOx
CG12505	*Arc1*	48130-GD	S/V	Nucleic acid binding	D
CG12846	*Tsp42Ed*	105847-KK	+	Tetraspanin	CGh
CG12908	*Ndg*	109625-KK	+	EGF-like calcium-binding	CA
CG1299	*CG1299*	108705-KK	EPL//wV+	Serine-type endopeptidase	P
CG13060	*CG13060*	49608-GD	+	--------------	CG
CG13941	*Arc2*	3464-GD	S/Bs	--------------	CG
CG14059	*Ilp8*	102604-KK	+	Insulin	CS
CG14196	*CG14196*	--------------	--------------	Monocarboxylate transmembrane transporter	Tra
CG14304	*CG14304*	106221-KK	+	Chitin	Cut
CG14545	*CG14545*	BL-35625	+	--------------	CG
CG14695	*CG14695*	14695R-1	+	--------------	CG
CG15209	*CG15209*	5534-GD	+	--------------	CG
CG15347	*CG15347*	107479-KK	+	DUF753	CGh
CG15623	*c-cup*	106603-KK	+	Cytochrome b5-like heme/steroid binding domain	Mit
CG18859	*Or19a*	BL-31892	+	Olfactory receptor	Neu
CG2065	*CG2065*	BL-34098	+	Glucose/ribitol dehydrogenase	M
CG2885	*RabX2*	103311-KK	+	Small GTPase mediated signal transduction	CS
CG30196	*CG30196*	30196R-3	+	LPS-induced tumor necrosis factor alpha factor.	CD
CG31062	*side*	1283-GD	+	CD80-like, immunoglobulin C2-set; Fibronectin, type III	Neu
CG31279	*CG31279*	109930-KK	V+	--------------	CG
CG32825	*Or19b*	109050-KK	sF//+	Olfactory receptor	Neu
CG34031	*CG34031*	--------------	--------------	--------------	CG
CG34165	*CG34165*	109149-KK	sF//+	--------------	CG
CG3767	*JhI-26*	36566-GD	+	CHK kinase	CS
CG4181	*GstD2*	109123-KK	sF//+	Glutathione transferase	RedOx

Spalt +					
**Name**	**Symbol**	**RNAi strain**	**Phenotype**	**Molecular**	**Molecular**
			**Age 48h**		
CG42255	*CG42255*	100425-KK	F/sS	Metalloendopeptidase	P
CG42352	*Ir40a*	100912-KK	+	Ion channel	Neu
CG42363	*CG42363*	--------------	--------------	--------------	CG
CG4373	*Cyp6d2*	101829-KK	+	Electron carrier	RedOx
CG4423	*GstD6*	105805-KK	F/S//Bs	Glutathione transferase	RedOx
CG5955	*CG5955*	102443-KK	sS/F	UDP-glucose 4-epimerase	M
CG6042	*Cyp12a4*	107088-KK	+	Electron carrier	RedOx
CG6142	*CG6142*	100131-KK	+	Glucose-methanol-choline oxidoreductase	M
CG7080	*CG7080*	104928-KK	+	--------------	CG
CG7356	*Tg*	103601-KK	Bs/S	Protein-glutamine gamma-glutamyltransferase	P
CG8927	*CG8927*	14167-GD	+	Cuticle	Cut
CG9521	*CG9521*	106710-KK	+	Oxidation-reduction	RedOx
CG9877	*CG9877*	--------------	--------------	--------------	CG
			**Age 24h**		
CG11486	*CG11486*	BL-42492	+	Protein kinase, catalytic domain	R
CG13316	*Mnt*	BL-42558	EPL/nW//+	Sequence-specific DNA binding	D
CG30059	*CG30059*	BL-28520	+	N-acetylglucosamine-6-sulfatase	M
CG32019	*bt*	BL-31546	+	Fibronectin, type III; Immunoglobulin I-set	CA
CG45050	*CG43674*	BL-31375	+	DNA binding	D
CG6544	*fau*	106560-KK	+	--------------	CG
CG7178	*wupA*	BL-31893	EPL	Tropomyosin binding; actin binding	Cyt
CG8663	*nrv3*	BL-29431	L	Potassium ion transport; sodium ion transport	Tra

Spalt +					
**Name**	**Symbol**	**RNAi strain**	**Phenotype**	**Molecular**	**Molecular**
			**Df//UAS**		
CG10102	*CR10102/Arc3*	--------------	--------------	Non protein coding gene	CR
CG10916	*CG10916*	107518-KK	PL/nW//wS-P	Zinc ion binding	D
CG1303	*agt*	104331-KK	+	DNA methylation	D
CG14121	*l(3)S147910/ver*	14121R-1	+	Telomere capping	D
CG15009	*ImpL2*	15009R-3	+	Insulin-like growth facgtor binding	CS
CG16928	*mre11*	30476-GD	+	Nucelase activity	D
CG18213	*CG18213*	18213R-2	+	Zinc finger	CGh
CG1925	*mus205*	103775-KK	sS/Bs//S	DNA binding	D
CG2999	*unc-13*	33609-GD	+	Intracellular signal transduction	CS
CG3008	*CG3008*	43732-GD	S-P	Protein kinase activity	P
CG31705	*CG31705*	7681-GD	+	--------------	CG
CG32021	*CG32021*	102286-KK	V-/S	--------------	CG
CG5202	*escl*	49982-GD	+	Histone methyltransferase activity	D
CG5247	*Irbp*	5247R-2	sS//S-P	DNA binding	D
CG6272	*CG6272*	101871-KK	EPL	Sequence-specific DNA binding	D

Spalt -					
**Name**	**Symbol**	**RNAi strain**	**Phenotype**	**Molecular**	**Molecular**
				**Age 24h/48h**	
CG10344	*CG10344*	48026-GD	+	Cysteine S-palmitoleyltransferase	P
CG10539	*S6k*	10539-R3	wS	Ribosomal protein S6 kinase	CS
CG10541	*Tektin-C*	100094-KK	EPL//S-P	Microtubule binding	Cyt
CG10576	*CG10576*	BL-34388	+	Aminopeptidase	P
CG11253	*CG11253*	105377-KK	+	Sensory perception of sound	CGh
CG11263	*CG11263*	31112-GD	+	Ribonuclease H-like	R
CG12182	*CG12182*	12182R-1	S-V//S-P	--------------	CG
CG12275	*RpS10a*	104132-KK	EPL//sS-P	Structural constituent of ribosome	R
CG12843	*Tsp42Ei*	101044-KK	sF//+	EC2 domain	CGh
CG13083	*CG13083*	106400-KK	PL/nW//wS-P	--------------	CG
CG1342	*Spn100A*	1342R-1	+	Serpin domain	CGh
CG13890	*CG13890*	108862-KK	Cell Pig./+	Dodecenoyl-CoA delta-isomerase	M
CG13897	*CG13897*	13897R-1	+	DNA binding	D
CG14394	*NijC*	39307-GD	Bs/S/V+	Ninjurin	CA
CG14485	*swi2*	BL-28530	+	Response to ecdysone	CG
CG14715	*CG14715*	104124-KK	+	Peptidyl-prolyl cis-trans isomerase	P
CG15236	*CG15236*	15236R-1	S/Bs	--------------	CG
CG15707	*krimp*	BL-37511	+	Tudor domain	R
CG17077	*pnt*	105390-KK	+	Protein binding	CS
CG2161	*Rga*	20826-GD	S // S/CD	G2/M transition DNA damage checkpoint	D
CG2849	*Rala*	43622-GD	S-P/nW//S-P	Ras	CS
CG30104	*CG30104*	106640-KK	sF//+	5'-nucleotidase	D
CG30488	*CG30488*	100513-KK	wV+/wBs	Allergen V5/Tpx-1-related	CGh
CG31262	*CG31262*	52605-GD	+	Sodium/solute symporter	Tra
CG31436	*CG31436*	21344-GD	S/V-	CHK kinase-like	CGh
CG31809	*CG31809*	BL-29424	+	Steroid dehydrogenase	M

Spalt -					
**Name**	**Symbol**	**RNAi strain**	**Phenotype**	**Molecular**	**Molecular**
			**Age 24h/48h**		
CG31810	*CG31810*	BL-29424	+	Steroid dehydrogenase	M
CG32364	*CG32364*	26045-GD	+	RNA recognition motif domain	R
CG33140	*CG33140*	18688-GD	EPL//S-P	--------------	CG
CG33302	*Cpr31A*	45310-GD	S/Bs	Cuticle	Cut
CG34215	*CG34215*	--------------	--------------	--------------	CG
CG3649	*CG3649*	3649R-1	sS-P//S-P	Phosphate:sodium symporter	Tra
CG40178	*CG40178*	110089-KK	sF//+	Redox homeostasis	Red-Ox
CG4086	*Su(P)*	BL-34955	+	Glutathione S-transferase	Red-Ox
CG41320	*CG41320*	109162-KK	sF//+	Thioredoxin-like fold	Red-Ox
CG42330	*Dscam4*	42882-KK	+	Cell adhesion	CA
CG43079	*nrm*	104295-KK	+	CD80-like, immunoglobulin C2-set	CA
CG43103	*CG43103*	--------------	--------------	--------------	CG
CG43117	*CG43117*	103091-KK	+	--------------	CG
CG4570	*CG4570*	109328-KK	sS	Transposase, Tc1-like	D
CG5671	*Pten*	35731-GD	LS	Phosphoprotein phosphatase activity	CS
CG6013	*CG6013*	6013R-2	+	HMG box domain	D
CG7160	*Cpr78E*	7160R-3	sS	Cuticle	Cut
CG7354	*mRpS26*	100067-KK	EPL//S-P	Ribosomal protein	R
CG7478	*Act79B*	BL-36857	+	Cytoskeleton	Cyt
CG7532	*l(2)34Fc*	7532R-1	+	Serine-type endopeptidase	P
CG7607	*CG7607*	9208-GD	+	Immunoglobulin-like domain	CA
CG7916	*CG7916*	101195-KK	sF//+	Haemolymph juvenile hormone binding	CGh
CG8023	*eIF4E-3*	BL-42804	+	Eukaryotic initiation factor 4G binding	R
CG8768	*CG8768*	8768R-2	sS	NAD-dependent epimerase/dehydratase	M
CG9333	*Oseg5*	100020-KK	S/V+	WD40-repeat	CGh

Spalt -					
**Name**	**Symbol**	**RNAi strain**	**Phenotype**	**Molecular**	**Molecular**
			**Age 48h**		
CG11051	*Nplp2*	15305-GD	+	Neuropeptide hormone	CS
CG14332	*CG14332*	41165-GD	+	--------------	CG
CG17876	*Amy-d*	109082-KK	+	Alpha-amylase	M
CG18363	*dic-04*	8416-GD	+	Mitochondrial carrier	Tra
CG33126	*NLaz*	101321-KK	sS/V+	Lipocalin/cytosolic fatty-acid binding	M
CG42798	*CG42798*	--------------	--------------	--------------	CG
CG42834	*CG42834*	--------------	--------------	--------------	CG
CG9486	*CG9486*	102802-KK	S/Bs	Acyl-CoA N-acyltransferase	M
			**Age 24h**		
CG13053	*CG13053*	9227-GD	+	--------------	CG
CG17134	*CG17134*	BL-42868	+	Aspartic-type endopeptidase	P
CG2360	*Ccp84Aa*	49694-GD	+	Chitin	Cut
CG3200	*Reg-2*	3200R-3	+	Phosphoglycolate phosphatase	M
CG32444	*CG32444*	101230-KK	+	Aldose 1-/Glucose-6-phosphate 1-epimerase	M
CG43114	*CG43114*	--------------	--------------	--------------	CG
CG6579	*atilla*	101803-KK	sF//+	--------------	CG
CG8800	*CG8800*	107835-KK	sF//+	Leucine rich repeat 4	CGh
			**Df//UAS**		
CG10041	*CG10041*	110063-KK	+	Serine-type endopeptidase activity	P
CG10225	*RanBP3*	104432-KK	F//+	Negative regulation of Wnt signaling pathway	CS
CG10382	*wrapper*	10382R-3	+	Immunoglobulin subtype 2	CGh
CG11212	*Ptr*	BL-28973	+	Hedgehog receptor activity	CS
CG11357	*CG11357*	108467-KK	L/Bs	Transferring glycosyl groups	P
CG11607	*H2.0*	15876-GD	L//sS-P	Sequence-specific DNA binding	D
CG11797	*Obp56a*	100850-KK	+	Odorant binding	CS

Spalt -					
**Name**	**Symbol**	**RNAi strain**	**Phenotype**	**Molecular**	**Molecular**
				**Df//UAS**	
CG11883	*CG11883*	38590/GD	S-P//S-P	5'-nucleotidase activity	D
CG12052	*lola*	BL-35721	L/F	Sequence-specific Dna binding transcription factor activity	D
CG12256	*CG12256*	12256R-4	+	Serine-type endopeptidase activity	P
CG12287	*pdm2*	102126-KK	Bs	DNA binding	D
CG1273	*CG1273*	4496-GD	+	--------------	CG
CG12952	*sage*	BL-25980	+	Sequence-specific DNA binding transcription factor activity	D
CG13618	*CG13618*	14714-GD	+	Haemolymph juvenile hormone binding	CGh
CG13848	*pinta*	13848R-4	+	Retinoid binding	M
CG14590	*CG14590*	41536-GD	+	Histone deacetylase binding	D
CG14869	*CG6107*	110157-KK	+	Zinc ion binding	P
CG15369	*CG15369*	15369R-2	+	Cysteine-type endopeptidase inhibitor activity	P
CG15739	*CG15739*	106016-KK	F//S-P	Phosphatase activity	P
CG16705	*SPE*	30972-GD	L//S-P	Serine-type endopeptidase activity	P
CG16712	*CG16712*	109238-KK	+	Serine-type endopeptidase inhibitor activity	P
CG16756	*CG16756*	102908-KK	S	Lysozyme activity	P
CG16947	*CG16947*	108103-KK	+	Zinc finger, RING-type	CGh
CG1725	*dlg1*	1725-R2	L//sS-P	Epidermal growth factor receptor binding	CA
CG1763	*nod*	48150-GD	+	DNA binding	D
CG1780	*Idgf4*	1780R-4	+	Imaginal disc growth factor receptor binding	CS
CG18024	*SoxN*	BL-25996	+	Chromatin DAN binding	D
CG18255	*Strn-Mlck*	--------------	--------------	Protein serine/threonine kinase activity	P
CG18410	*Ude*	18410R-2	+	DNA binding	D
CG18657	*NetA*	18657R-2	+	Netrin domain	CGh
CG1869	*Cht7*	42877-GD	sS-P//sS-P	Chitinase activity	Cut
CG2056	*spirit*	3285-GD	+	Serine-type endopeptidase activity	P
CG2488	*phr6-4*	2488R-3	+	DNA (6-4) photolyase activity	D

Spalt -					
**Name**	**Symbol**	**RNAi strain**	**Phenotype**	**Molecular**	**Molecular**
			**Df//UAS**		
CG2706	*fs(1)Yb*	110056-KK	+	Tudor domain	D
CG30069	*CG30069*	25562-GD	S/Bs	--------------	CG
CG3097	*CG3097*	--------------	--------------	Zinc ion binding	P
CG3100	*b6*	14526-GD	+	Neuronal pentraxin receptor activity	CS
CG31098	*CG10634*	21300-GD	V+	Transferase activity	P
CG31721	*Trim9*	100767-KK	+	Zinc finger, B-box	CGh
CG31764	*vir-1*	102534-KK	+	--------------	CG
CG31839	*nimB2*	105925-KK	+	Insulin-like growth factor binding protein	CS
CG31884	*Trx-2*	36297-GD	+	Disulfide oxidoreductase activity	D
CG32029	*Cpr66D*	112709-KK	S/Bs	Structural constituent of chitin-based cuticle	Cut
CG32055	*CG32055*	6335-GD	wS	Insulin-like growth factor binding	CS
CG32261	*Gr64a*	103342-KK	S/F	Taste receptor activity	CS
CG3240	*Rad1*	12676-GD	+	Exonuclease activity	D
CG32491	*mod(mdg4)*	BL-32995	F	DNA binding	D
CG3289	*Ptpa*	41913-GD	+	Protein tyrosine phosphatase activator activity	CS
CG33197	*mbl*	BL-29585	Bs/P	DNA binding	D
CG33960	*Sema-2b*	BL-28932	+	Sema domain	CS
CG33970	*CG33970*	33090-GD	+	Transporter activity	Tr
CG34406	*CG44002*	43767-GD	+	Zinc ion binding	D
CG42614	*scrib*	BL29552	PL/nW/sS-P/CD	Protein binding	CA
CG4322	*moody*	4322R-1	S-P//S-P	Melatonin receptor activity	CS
CG4379	*Pka-C1*	101524-KK	S-P	Protein binding	CS
CG4398	*CG4398*	4398R-1	+	--------------	CG
CG4432	*PGRP-LC*	4432R-1	+	Protein dimerization activity	D
CG4565	*CG4565*	BL-32893	+	Histone-lysine N-methyltransferase activity	D
CG5249	*Blimp-1*	5249R-2	S-P/F	Nucleic acid binding	D

Spalt -					
**Name**	**Symbol**	**RNAi strain**	**Phenotype**	**Molecular**	**Molecular**
				**Df//UAS**	
CG6134	*spz*	BL-28538	+	Protein homodimerization activity	CS
CG6713	*Nos*	BL-28792	+	Nitric-oxide xynthase activity	D
CG7201	*CG7201*	101198-KK	sF//+	--------------	CG
CG7420	*CG7420*	BL-32510	L	Regulator of chromosome condensation, RCC1	D
CG7577	*ppk20*	36659-GD	S	Sodium channel activity	Tr
CG7734	*shn*	105643-KK	sS-P//sS-P	RNA polymerase II activating transcription factor binding	D
CG7897	*gp210*	--------------	--------------	Protein import into nucleus	CS
CG7906	*CG7906*	BL-30519	+	Kazal domain	P
CG8084	*ana*	BL-27515	S/V+//S-P	Growth factor activity	CS
CG8589	*tej*	103264-KK	+	Tudor domain	D
CG8675	*CG8675*	8675R-1	S-P/nW//S-P	--------------	CG
CG8773	*CG8773*	10203-GD	+	Metallopeptidase activity	P
CG8780	*tey*	106065-KK	Bs/S	Negative regulation of transcription	D
CG8945	*CG8945*	46925-GD	F	Zinc ion binding	P
CG9355	*dy*	9355R-1	L//S-P	Structural constituent of chitin-based cuticle	Cut
CG9460	*Spn42De*	9460R-3	+	Serine-type endopeptidase inhibitor activity	P
CG9850	*CG11169*	107199-KK	sF//+	Metalloendopeptidase activity	P
