# Supplementary material for: The Spalt Transcription Factors Generate the Transcriptional Landscape of the Drosophila melanogaster Wing Pouch Central Region
Source: PLoS Genet. 2015 Aug 4;11(8):e1005370. doi: 10.1371/journal.pgen.1005370 (PMC4524721; doi:10.1371/journal.pgen.1005370)
Supplement: S6 Table — (PDF) [file pgen.1005370.s021.pdf]

Concentration (ngr/μl)	Ratio 260/280	Ratio 260/230	Sample volume (μl)	Sample μgr
sal ^{EPv} -Gal4 UAS-GFP; tub-Gal80 ^{ts} 24h				
256,75	2,09	0,94	20	5,14
327,39	2,08	0,57	12	3,93
312,07	2,08	0,96	10	3,12
256,51	2,10	0,50	15	3,85
238,95	2,10	0,50	10	2,39
sal ^{EPv} -Gal4 UAS-GFP; tub-Gal80 ^{ts} 48h				
341,09	2,08	0,57	12	4,09
336,21	2,05	1,01	15	5,04
331,09	2,09	0,64	15	4,97
371,65	2,09	0,63	12	4,46
sal ^{EPv} -Gal4 UAS-GFP / UAS-salm-i; tub-Gal80 ^{ts} / UAS-salr-i 24h				
353,12	2,08	0,94	10	3,53
265,25	2,08	1,52	15	3,98
286,97	2,09	1,41	15	4,30
322,38	2,09	0,63	15	4,84
sal ^{EPv} -Gal4 UAS-GFP / UAS-salm-i; tub-Gal80 ^{ts} / UAS-salr-i 48h				
236,95	2,00	0,41	10	2,37
325,06	2,08	0,76	12	3,90
314,65	2,09	1,03	12	3,78
235,28	2,10	0,43	15	3,53
