# Supplementary material for: The Spalt Transcription Factors Generate the Transcriptional Landscape of the Drosophila melanogaster Wing Pouch Central Region
Source: PLoS Genet. 2015 Aug 4;11(8):e1005370. doi: 10.1371/journal.pgen.1005370 (PMC4524721; doi:10.1371/journal.pgen.1005370)
Supplement: S7 Table — Each oligonucleotide contains the sequence of the corresponding RNA polymerase (T3: ATTAACCCTCACTAAAGGGA; T7: TAATACGACTCACTATAGGG). (PDF) [file pgen.1005370.s022.pdf]

**Name**	**Symbol**	**Primer AS T3**	**Primer S T7**
CG10041	*CG10041*	TCAAGCGGTTTGAGGAAAAC	CAACTGTGTCGACCACGAAG
CG10225	*RanBP3*	GTTGTCGATGGCCGTTAGTC	CAAGTGCAGGTGAAGACAGC
CG10576	*CG10576*	TGCTTTGCTTTTTCGTTGC	TTAAGCACACGGTTCTGCTC
CG1058	*rpk*	GGCGCTTCAGATTGGTAAAG	TAGGCCCGAAAACGTTAGTG
CG11155	*CG11155*	CTTCGTTTTCGCGGTCTATG	TCGACAATGCTGGACTATGC
CG11212	*Ptr*	CGTCGTCTAAATCGCTCCTC	TCACCGCTCACATCTGCTAC
CG11263	*CG11263*	TAAGAGTTGGAACCCGAAGC	GATGGAAACGAGAGCTTTGC
CG11486	*CG11486*	TGCGTCCATGATCAGGTTAG	GACGCCCAACTTCTATGCAC
CG11584	*CG11584*	TGTAGGTCACCTGCTGTTGC	AATTGCCATTCCAGTTCCTG
CG11897	*CG11897*	AACATAATCCTGGCGCAGAC	GCTCAGCTTTATCCCACTGC
CG12052	*lola*	TGTAGGGGCAGTAGGGACAC	TAGATCCCTCGACCATTTCG
CG12224	*CG12224*	GTTAATCCGCAGCAGCTTTC	GCCAGCTACTTTCGTCAAGG
CG12367	*Hen1*	CAGCGTTCCTCCTCTTGTTC	AAATTCTGCAGGGGAATGTG
CG12952	*sage*	GGAGCGCAGTAAGTCAAAGG	AGTTTTGGTGCCCGTATCAG
CG1303	*agt*	ACTTTTCATCGGCCAGAAGC	AAACACCCAGATTCGTTTGG
CG13083	*CG13083*	TGGTGTGACGGATTTTTGTG	TGAGGATCTGGACTCCATCC
CG13848	*pinta*	CATAGAAATCGGCGTTCTCC	CTGCTGCTAAAGCTCGATCC
CG13890	*CG13890*	CAGCTGTGCGATTCATTCAC	ATATGCTGCCCCTCATCTTG
CG13937	*CG13937*	CGTAGAGCTCCAGCATTTCC	TGCGTGAGGCACAGAACTAC
CG14394	*NijC*	TACAATGTGGCAAGCAAGTG	CATTCTGTTCGTGGTCATCG
CG14485	*swi2*	CACAATCCAAGGAACTCGAC	CGGGACAATCCCCACTAC
CG14590	*CG14590*	CTCCAAGCCAGCTTCGTTAC	CACGAGTCAATGGAATGCAG
CG14869	*CG6107*	ACTGGCCAATGCGATTGTAG	GTGAGGGTGTGGAGAAGAGG
CG15400	*CG15400*	ATATAGTGGCGAGGCCAAAG	TTCTCAGCGTTCATTTGGAG
CG15892	*CG15892*	AGTCATAGCGGGTGCACTTC	ATGTCTCGGATTGGGATGAG
CG17077	*pnt*	CGGTCCACGAAATGAAACTC	ACTGTCAATGGCAGCGGTAG
CG17104	*CG17104*	GGCTGCCTCTTCCTTTACTG	TGCCATTCTGCTACTGATCG
CG17134	*CG17134*	GCCAGGGTGAAGACAGTACC	GCAGTGTTAAGGCCGAGAAG
CG17533	*GstE8*	TTGAGGAGGGTCACCAAATC	GAGGCTAGTCCTCCAGTTCG
CG1780	*Idgf4*	GAACTTGTCGTGGCCAGTG	TCGATGTGCCCGCTATTATC

Name	Symbol	Primer AS T3	Primer S T7
CG17803	*CG17803*	TGTTGCGCGTATAGAGATCG	CTACCGCATCACATTTGCTC
CG18188	*Damm*	CGTTCGACCTCAGCCTTAAC	GCAGTTCCCTCAGGATTCTC
CG18255	*Strn-Mlck*	GCGCTGATAGCGATAGTTCC	TTATCGGGGTGAAAACGAAG
CG18278	*CG18278*	GTGTTGTTCCATGCATCCTG	TGGCGGGAAGTACTTGAATC
CG18410	*Ude*	GTCTTCTTGGCCAGCTTTTC	CGAGATCCGGACTACCAGAG
CG1851	*Ady43A*	TTGTTGTGCGCTATCTGAGG	CTCTCGGTGCCTTACAAACC
CG2056	*spirit*	GAATTCGGGACTGAAACTGG	GAGCCACCCAGTCAGGTG
CG2184	*Mlc2*	TTCATCTTGCCGTGTGTAGC	TGATCGACGACAAGAACCAG
CG2488	*phr6-4*	TCTCCTCGAAAGACGACTCC	GCATGGAGGAATCCCTAAAG
CG2999	*unc-13*	ACCCATAAAGCGCTTGTGAC	GTCAAGGCCTCCAGAGACAG
CG30488	*CG30488*	TGACTGTTCATCATGCATCC	TGCGAGCTTGATACCAGAAG
CG3074	*Swim*	CAACTTCACCGAGTGGAACC	GTGGACCCAATCATCTCCTG
CG3081	*CG3081*	CACCACCAGAACCCTACCAC	CATCCAATCATGTCGCAAAC
CG31098	*CG10634*	TGCTCTCAAACATGGTCAGC	GAACCGGCAGCAAGAACTAC
CG31262	*CG31262*	ACCACAACTGCTGTGACGAC	ATCGGTGGCTTTTCATTCTG
CG31436	*CG31436*	CTTCCAAAGACCCGATTGAC	AGGCAGAGGGACACAAGAAG
CG31839	*nimB2*	CTGCAGATGCAGAAGTTTGG	CAATTCGCGGGATCAGTTC
CG31875	*CG31875*	TTTTTGGTTGGCATCAACAG	CACGCAAGGAGAAACGAGAC
CG3200	*Reg-2*	TCCTCGCCGTATTTCTTGAC	TGCACAACTTCTCCAACCAC
CG32055	*CG32055*	CTTTCGACGATTCCTTGAGC	GGGAATATTTTACGCGTTGC
CG3240	*Rad1*	AATCAGTGTTGAGCAAAGGAA	GCAAGTCTCCGAAGATGGTC
CG32444	*CG32444*	TTGGGGGTTGGACTTTATTG	GGGAGGTGGTACAGAAGTCG
CG32491	*mod(mdg4)*	AGGACTTGGTCGCATTGTTC	GTCTTTCCGTCGAGTTGTGC
CG32625	*CG32625*	TCCATTAATCGCTTGCGTTC	TGAAGTTGACGAAGGGGAAG
CG32788	*Crg-1*	AGTTGTTGCCGCTGCTTTT	TACTTCCTCCCGCTCCTACC
CG33048	*Mocs1*	CTTTGTTGCCAAACAAGCAC	AGCCGAAAAACAAACTCCTG
CG33140	*CG33140*	ATTGGCTTTGGTTGATCTGG	AACGGGTCCGATACCTTTTC
CG33302	*Cpr31A*	AGGCCGTCTTCTTGTTCTCC	CACCTATGCCGGCTATCATC
CG3397	*CG3397*	GTTAATCCGCAGCAGCTTTC	GCCAGCTACTTTCGTCAAGG
CG34002	*CG34002*	TCACCTTCACCACCATCATC	GTCACCAAAGTGCGGAAAAG

Name	Symbol	Primer AS T3	Primer S T7
CG34215	*CG34215*	GGTAATCTGCGTTTGGGTTT	ATTTGAAGGCTCCAAGATGC
CG3649	*CG3649*	TTGGTGATGCTTCTGTTTCG	GCATCGTGTGGTGCCTTAC
CG40178	*CG40178*	AAAACCAGTTGTGGCACTTC	ATGATTACGGTGCCGAAAAG
CG40300	*ago-03*	GTGCTTGAATGTAGGCATCG	TTTTCCTCCCATTGACTCTG
CG40452	*Snap25*	AAACCATGAGGACCGTTTTG	TCTAATGAAGCGCGGATAGC
CG41320	*CG41320*	CCAGTTGTGGCACTTCTCTG	GACATTGCATTTTATCAGAAGTTATCA
CG4174	*CG4174*	AGTTTGGCAGGATTCGATTG	GTTGTAACTGCCTGCCTTGG
CG42516	*CG42516*	TCGGTCTGATCGTCCTCTTC	TGTGCACGCACATTTCTAAC
CG43079	*nrm*	TTTAGGGTTGCCATTTTTGG	TGCTCAAAGACCCAAGGAAC
CG43103	*CG43103*	AAACCGAATGTGTCGTATTTTT	CCAAGCCGGAGGCTAGTAGT
CG43114	*CG43114*	TTCGCCTTTATTCCGATTTG	CTGACAACTCATCAGCATGGA
CG43117	*CG43117*	TGCCCATTATTAGAAATGTGTTTT	CGCACAATGAAGCTAAGCAC
CG43144	*CG43144*	TTGTCTGTTGGTTGTCAGTCG	GATGGCTCAGGCTTTCCTC
CG43646	*CG43646*	CAGGGTTCGGGTATAGCTTG	TTGGCACAGATTTCACTTCG
CG43676	*CG43676*	TTTATTGTCCTGTAATTTCTTTCTGA	ATGCACTCCGCATCACTGTA
CG4379	*Pka-C1*	ACTCCTTGGCACACTTCTCG	GTGTCATGATCGTCCAGCAC
CG45050	*CG43674*	CGTTCCATCTTTGCTCCTTC	TGGACAAGAGCATTGAGCAC
CG4570	*CG4570*	AGCTTGGCTAGCAGAACTCG	CAGCTTGCTCGCCATAAAAC
CG4594	*CG4594*	GCTCCCATAACCTTCTGCAC	TGGATTGCCCTCTATGGAAC
CG5229	*chm*	GCTGGAATCACTTCCCTCAG	GGATCCACCAGTTCGGAAAC
CG5671	*Pten*	ATCTTCCTCGCCATCTTCG	CTGAAGCGTCCGAAAAAGAG
CG6134	*spz*	GCAGCAGGAAGGGATCTTG	CTTTGTCTTCCCCGATTCC
CG6579	*atilla*	TTGCAGGTGTAGCAGCTCTC	CGGCCATCAAGTGTTATCAG
CG6658	*Ugt86Di*	TGGTACTGCCACCAGTTCAG	GTGCCCCACATAATGCTACC
CG6713	*Nos*	GATTACGGCAGCCAAAGAAG	TTCGCTGACTGTGAATACGG
CG7178	*wupA*	TTCATCAAGGCGTTCAGATG	AGAAACCCGACTGGTCCAAG
CG7420	*CG7420*	CATTTTCGGTGCTGTTGTTG	AGCCATCACGTTGACCAAAC
CG7590	*scyl*	CGTATACTCCGGACTGATGG	GGACCTGATGTCCAAGAAGG
CG8023	*eIF4E-3*	CCTCAACGGTGTTGAAGCTG	AAGGTTCGTTTATCGCATTG
CG8573	*su(Hw)*	ATTTGTCAATGCGCAAGATG	ATTATGGAACGCACGGATTG

Name	Symbol	Primer AS T3	Primer S T7
CG8589	*tej*	CACAGCATAAGCTGGAATGG	CAAATGGCATGGATAACTGC
CG8768	*CG8768*	TTTCGGAGGGCGTGATTC	CGCTGTTGTGGGTCATGG
CG8780	*tey*	CAAGGCGTACTTCCTCTTGC	CTGCATAATGCGGGGAAG
CG8800	*CG8800*	GAAAGTTCGCATGGAAAACC	TTGAGAAGATATTCGGCTTATCG
CG9214	*Tob*	TGGCACTTATTGGCCCTATC	CAATGTGGACTGCGGTAGTG

Name	Symbol	Primer AS T7	Primer S T3
CG10102	*CR10102/Arc3*	TACTCCTCGTGCTGCTCCTC	CTCATCCGCGAACACTTCTC
CG10382	*wrapper*	GCTCAACACTTCCGCTTTTC	GATGTGGCTCTGGTGGACTC
CG11357	*CG11357*	TCGACAGAGCATCAGATTGG	AAATCATCGCCGATTTTAGC
CG11797	*Obp56a*	ACTTGGAGGCGGTATCACAC	CCCTTAATCTGAGCGACGAG
CG11883	*CG11883*	AAGGGCAATGATGAGGTCAC	CAGGGAACTCCTGACGAGAC
CG12256	*CG12256*	CAAACCTTTCCAAGGCACAG	CATGAACGGAGTAACTTATTTTGTG
CG12287	*pdm2*	GACCAGAGAGTTTGGCTTGG	TTGAGGACATCGGGAAAGTC
CG14121	*l(3)S147910/ver*	TGCATTGTTTTATGCCTTCG	TGGATTTTAATCAGAGTTTCGAG
CG15739	*CG15739*	TCCGCATAGTAGTCCGGTATG	TCGACGTGGACTTCAATCTG
CG15784	*CG15784*	AGCGCTGCTGTCTTTTCTTC	CACCAAAAGGACCAAGAAGC
CG16756	*CG16756*	CGTTTCGGCAATATCGTTG	TGCTCTCGAATTGTGAGTGG
CG16928	*mre11*	TCGCATTAAACATGCAGGAC	TCCTGTTTCCCTGGAGATTC
CG16947	*CG16947*	GCTTGTAGTGAGCGCATCC	CGCCGAATATCGCTAAATG
CG1725	*dlg1*	GCTCCTCCTCATCCTGACTG	GGATTCGGATACGGACTCG
CG1763	*nod*	ACCTAGCAGCTCCAGCAGAG	CAGCAGCAGCTCAAAGTACG
CG18024	*SoxN*	CAGAGCTGTATTCCCGCTTC	TGCATTGAGCAGCCTAACTG
CG18657	*NetA*	GATCGAAGTGGAATGGCTTG	GCATTCCGGACTTTGTGAAC
CG1925	*mus205*	CACGATCATTGCCCAGTATG	ATTCGGCAAATCCAGATGAG
CG2706	*fs(1)Yb*	CCGAATCAATCCATTTTTGG	TGTTGCTCCAACTGCTGAAC
CG2914	*Ets21C*	GTCGTAGTTTCCCGCAGAAG	AATCCAGCTGTGGCAGTTTC
CG30196	*CG30196*	ATTCTCATTGGAACGCTTCG	CGAGAGTCTGGATGGCAGTG
CG3100	*b6*	AAATTTCATTGCCCACAAGC	TACAGACGCGTGGAAGATTG

Name	Symbol	Primer AS T7	Primer S T3
CG32261	*Gr64a*	GTGCCACTGTCTGTGTGGTC	CGTGCACTTTTGTGTGGAAC
CG32364	*CG32364*	GAAACAGCCCTTTTGTCTGG	CGAGGAATCACAGTTGTTCTCC
CG3289	*Ptpa*	CACTGCCCCAGATGAAGG	GGCAATTGCGAAGAAGGTG
CG33960	*Sema-2b*	ACACCGGCGACTGTACTACC	AGGACGTTCGTGATTTTTCG
CG34406	*CG44002*	GATATAGCCGCGTTCTCGAC	TATGCCCACCATGTGTGAAG
CG42330	*Dscam4*	GCATTATTGTGTGCCGTGAC	TAGGTCCCTTCGTGGAAATG
CG42614	*scrib*	ACTCGGTTTCGTCTTGGATG	CGATGAGGATGATGATGACG
CG4398	*CG4398*	GATTTCGCCTTGAACTCTGC	TGAGACTGGTGCTGCAATTC
CG4432	*PGRP-LC*	AATGCGGGGTTATCCTGTC	TGTTCCAATGCGAAACGTAG
CG4565	*CG4565*	CGCAGCTGTGGTTCAGGTAG	ATGGACGAATCTGAAACAGC
CG5249	*Blimp-1*	ACACATAGGGCCTGTCGTTC	TCCAATCTGGGACAGAATCC
CG7080	*CG7080*	CTTGTAGCAGCCCTCCACTC	CTTTGGTTAGCTGCCAGGAC
CG7080	*CG7080*	CTTGTAGCAGCCCTCCACTC	CTTTGGTTAGCTGCCAGGAC
CG7201	*CG7201*	CCTCCACTACGCCATAGAGC	AAGGTGTTAAGGCGATGCAG
CG7577	*ppk20*	GGCCTGGCAGATACTTACG	ATTCGGTCAAACCGACAGAG
CG7734	*shn*	TGTTTGAGGAGAGCGATGTG	ACGGCCAAGCATTACAAAAC
CG7897	*gp210*	ATCAACAATGGCGAAAAAGG	TTCGCCGAGGAAGTACAAAC
CG7906	*CG7906*	GGCACAAATGGACAGTGATG	ACTACGGAAGCCGCAACTAC
CG8773	*CG8773*	TTCGTCGTACAAAAGCGATG	ACAAGTGAACCCGGCACTAC
CG8799	*l(2)03659*	AGCCAAACTAATGCCACCTG	CAGTCCGAAGAGCTGGGTAG
CG8945	*CG8945*	ATTTGGCCTTGAAGTTGTGC	TTGTGCTCTTGATCGTGGTC
CG9333	*Oseg5*	AGTGGCTTGATGGATATGTGC	CGCGAATCTGCAAGAAAAAC
CG9460	*Spn42De*	GCTGCCTGTAGAGGGACATC	CGTGTTCACTTCTGCTGCTC
